# High‐Throughput Engineering and Modification of Non‐Ribosomal Peptide Synthetases Based on Golden Gate Assembly

**DOI:** 10.1002/anie.202508967

**Published:** 2025-10-11

**Authors:** Adrian Podolski, Timon A. Lindeboom, Leonard Präve, Maryam Dehghan, Hannah A. Minas, Janik Kranz, Daniel Schindler, Helge B. Bode

**Affiliations:** ^1^ Department of Natural Products in Organismic Interactions Max Planck Institute for Terrestrial Microbiology 35043 Marburg Germany; ^2^ Molecular Biotechnology Department of Biosciences Goethe University Frankfurt 60438 Frankfurt am Main Germany; ^3^ MaxGENESYS Biofoundry Max Planck Institute for Terrestrial Microbiology 35043 Marburg Germany; ^4^ Center for Synthetic Microbiology (SYNMIKRO) Phillips University Marburg 35043 Marburg Germany; ^5^ Department of Chemistry Phillips University Marburg 35043 Marburg Germany; ^6^ Myria Biosciences AG Hochbergerstrasse 60 C Basel 4057 Switzerland; ^7^ Present address: Synthetic Genomics, Center for Molecular Biology of Heidelberg University (ZMBH) Heidelberg 69120 Germany

**Keywords:** Engineering, Non‐ribosomal peptide synthetase, NRPS, Peptide, Synthetic biology

## Abstract

Non‐ribosomal peptide synthetases (NRPS) are multimodular enzymes that produce complex peptides with diverse biological activities, potentially being used as clinical drugs. However, the pharmaceutical applications of such natural peptides often require further derivatisation and modification of the peptide backbone, mainly performed by chemical synthesis. A sustainable alternative resembles the in vivo engineering of NRPS to change and modify the enzyme properties rationally and, thus, the produced products. The novel NRPS engineering approach, the eXchange Unit Thiolation domain (XUT) concept, allows the efficient modular assembly of different natural NRPS fragments to form hybrid NRPS that produce defined peptides. In this study, we describe a Golden Gate Assembly (GGA)‐based method for efficient high‐throughput generation of novel and engineered NRPS libraries utilising the XUT concept. This method was applied to generate over 100 novel NRPS with the possibility of changing starter, elongation, and termination modules, respectively. Additionally, we applied this method for targeted modification of the xenoamicin biosynthetic gene cluster (BGC) *xabABCD* from *Xenorhabdus doucetiae*, resulting in the generation of 25 novel xenoamicin derivatives.

## Graphical abstract

A Golden Gate Assembly (GGA)‐based method was developed for the efficient assembly of natural and engineered non‐ribosomal peptide synthetases (NRPS). This method has enabled the creation of NRPS libraries to generate novel peptides in high‐throughput as well as the targeted derivatisation of natural products (NPs).

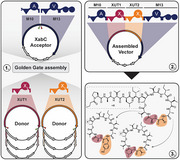

## Introduction

Microbial secondary metabolites or natural products (NPs) are structurally complex molecules with a diverse range of activities and functions. They play an important role in human health as they provide clinically used drugs or precursors for such drugs, including antibiotics or anticancer treatments.^[^ ^1^, ^2^, ^3^ ^]^ NPs often require optimisation of their selectivity, stability, and/or (oral) availability. Although chemical synthesis or medicinal chemistry methods are well established to optimise these parameters,^[^ ^1^, ^4^, ^5^ ^]^ they can be challenging and expensive. Conversely, advances in molecular biology, especially synthetic biology, can facilitate such modifications.^[^ ^6^ ^]^ Many clinically used NPs or NPs with promising bioactivities are derived from non‐ribosomal peptide synthetases (NRPS) or hybrids between NRPS and polyketide synthases (PKS), which are large, multifunctional enzymes with a characteristic modular nature.^[^ ^2^ ^]^ This modularity offers an ideal entry point for modification, engineering, and subsequent compound derivatisation, even at the backbone level.

Each NRPS module is responsible for selecting, activating, and binding a specific amino acid (AA) to the NRPS and connecting the covalently bound AAs, resulting in a growing peptide chain from the N‐ to the C‐terminal part of the NRPS. NRPS modules can be divided into initiation, multiple elongation, and termination modules. Each elongation module contains a condensation (C) domain that catalyses the peptide bond formation between the upstream and downstream AA. An adenylation (A) domain is responsible for selecting and activating a specific AA, using ATP as a co‐substrate. The thiolation (T) domain is posttranslationally activated by a 4′‐phosphopantetheinyl transferase and covalently binds the AA to the NRPS. The initiation or starter modules are responsible for the initiation of peptide synthesis and frequently contain a C_Starter_ domain that facilitates the attachment of fatty acids to the N‐terminus. Termination modules mainly contain a thioesterase (TE), which is positioned after the T domain and catalyses the release of the peptide as a linear or cyclic (depsi) peptide.^[^ ^2^ ^]^ Several other domains add to the structural complexity of non‐ribosomal peptides through additional cyclisation, epimerisation, oxidation, hydrolysis, and reduction. Additionally, epimerisation domains can occur as hybrid condensation/epimerisation domains (C/E), performing both functions.

Since modification of the overall modular structure or domain specificity was the goal after their biochemical principles were elucidated, several methods have been developed during the last 30 years. Beginning with the exchange of individual AAs in the substrate binding pocket of the A domains, the exchange of partial or complete A domains to change the incorporated AA, and the complete exchange of multiple domains and modules.^[^ ^7^, ^8^, ^9^ ^]^ Although several of these methods function well in specific NRPS systems but show low production titers of the desired peptide when applied to other systems, we and others^[^ ^10^, ^11^ ^]^ tried to establish more broadly applicable strategies such as the eXchange units (XU)^[^ ^12^ ^]^ and eXchange Unit Condensation domain concepts (XUC)^[^ ^13^ ^]^ applying fusion sites within the A‐C didomain linker or within the pseudo‐dimeric structure of the C domain. The most recent eXchange Unit approach, the eXchange Unit Thiolation domain (XUT),^[^ ^14^ ^]^ identified fusion sites directly before the T domain, described as XUT^I^ or within the conserved motif FFxxGGxS in loop 1 and the N‐terminus of helix 2 within the T domain, described as XUT^III^ and XUT^IV^.^[^ ^14^, ^15^ ^]^

Despite the notable advances in the novel XUT method, allowing the assembly of NRPS and even PKS parts from various origins (since T domains in NRPS and PKS are structurally similar),^[^ ^14^, ^16^ ^]^ cloning and heterologous expression of natural or engineered NRPS is still time‐consuming and challenging.^[^ ^17^, ^18^, ^19^, ^20^ ^]^ Methods such as transformation‐associated recombination (TAR) cloning^[^ ^21^, ^22^ ^]^ or Gibson Assembly^[^ ^12^, ^13^, ^14^, ^23^ ^]^ have been frequently used for the assembly of NRPS systems. Although these methods are precise and efficient in assembling multiple NRPS‐encoding gene fragments into plasmids, they are time‐consuming, with fragments that are often not reusable, making it difficult for high‐throughput applications. Unlike previous engineering methods, the XUT concept uses a conserved motif within the T domain, allowing the use of alternative cloning approaches.

Golden Gate Assembly (GGA) has become a standard cloning method and provides a baseline for other strategies such as MoClo and GoldenBraid 2.0.^[^ ^22^, ^23^, ^24^ ^]^ GGA utilises Type IIS restriction enzymes, generating DNA fragments with a three‐ or four‐nucleotide overhang that are joined by a T4 ligase in a one‐pot reaction.^[^ ^24^, ^25^, ^26^ ^]^ As the enzyme cleaving site is adjacent to the recognition site, sequence‐independent and thus definable sticky ends can be created, which allow the ordered assembly of such fragments.

Here, we describe a GGA‐based approach that facilitates high‐throughput cloning for engineered NRPS systems and the generation of modular NRPS libraries. This method combines GGA with the XUT approach, harnessing the two conserved glycines from the XUT^IV^ fusion site for predefined overhangs, thereby enabling a scarless assembly. To demonstrate the functionality of this method and its high‐throughput potential for modular‐based NRPS libraries, we present the general construction of an acceptor/donor system, as well as its integration into a model system and subsequent derivatisation at the native xenoamicin‐producing NRPS.

## Results and Discussion

### NRPS Golden Gate Assembly Design and Workflow

In previous works, the construction of many NRPS‐encoding plasmids relies on Gibson Assembly.^[^ ^12^, ^13^, ^14^, ^16^, ^23^, ^27^ ^]^ Although this method efficiently utilises specific overhangs to ensure successful assembly, it lacks high‐throughput potential due to the necessity of designing specific and often unique overhangs for each plasmid assembly. In contrast, a restriction enzyme approach, such as the GGA, obviates the need for such unique overhangs. In order to facilitate a scarless GGA approach, fusion sites used for XU and XUC are located within linker regions.^[^ ^12^, ^13^ ^]^ This necessitates regular alterations of amino acids, resulting in the implementation of scars. The XUT concept, however, provides three potential fusion sites from which XUT^III^ and XUT^IV^ are located within a conserved motif:^[^ ^14^ ^]^ The conserved “FFxxGGxS” motif in loop 1 and the N‐terminus of helix 2 of the T domain,^[^ ^14^, ^15^ ^]^ which provides conserved AAs to create universal overhangs needed for the GGA. Although the high phenylalanines of XUT^III^ are only encoded by two different codons each, we used both conserved glycines from XUT^IV^, which is by far the most abundant sequence in all bacterial T domains at this position (Figure S1). They provide enough codons to create 16 scarless overhangs (Figure S2), which can be used for acceptor and donor plasmid design, with the aim of combining starter, elongation, and termination modules as XUTs. Additionally, XUT^IV^ also showed great overall functionality of engineered NRPS systems^[^ ^14^ ^]^ and was therefore selected for this approach.

**Figure 1:**
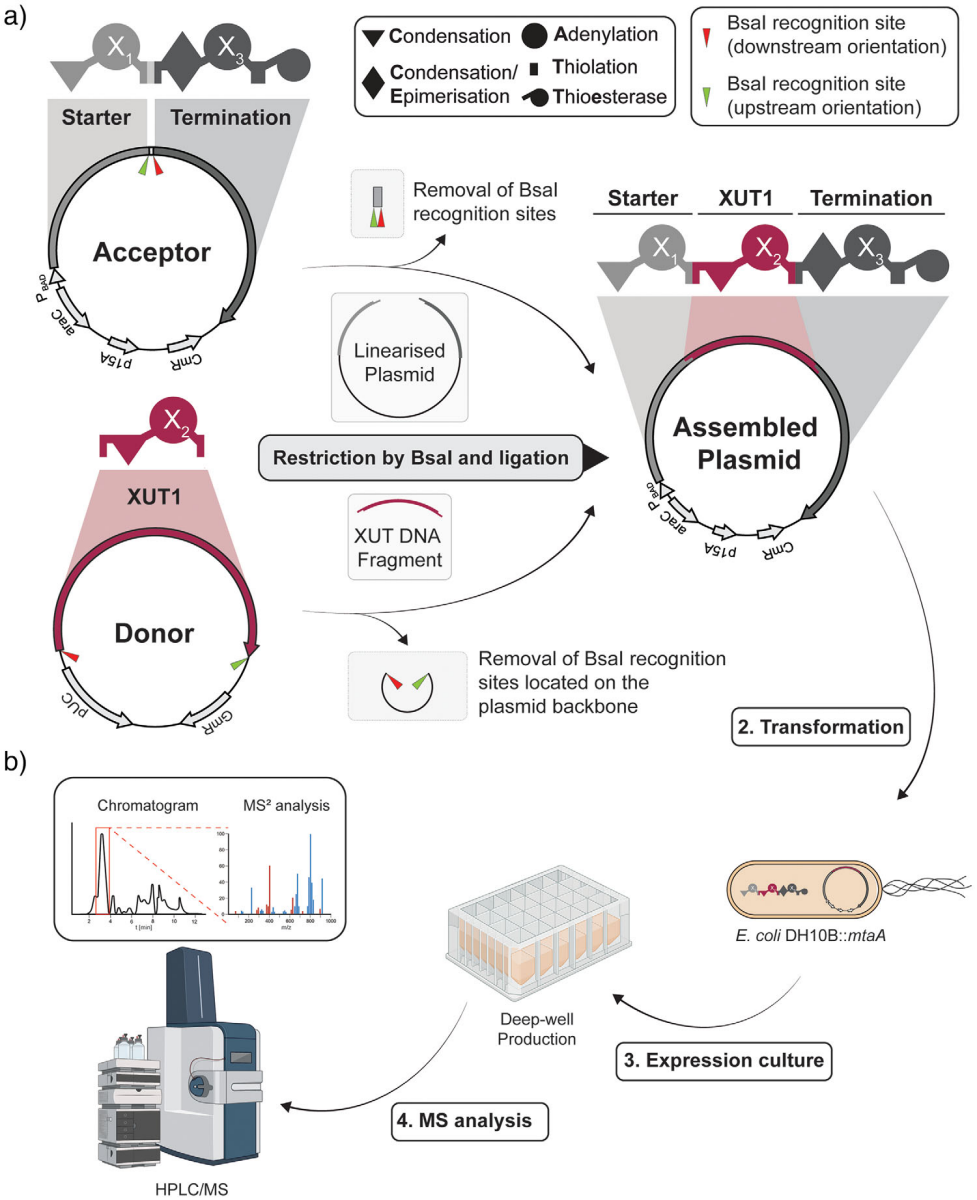
Schematic representation of the Golden Gate Assembly (GGA) acceptor and donor plasmids for XUT‐based NRPS engineering and the high‐throughput workflow. a) Schematic representation of an acceptor and donor plasmid with indications of the NRPS‐encoding regions highlighted with the respective schematic NRPS. Both the acceptor and the donor plasmid contain BsaI recognition sites (red and green arrows) to create two different overhangs. The acceptor plasmid harbours a starter module (Starter; light grey) and a termination module (Termination; dark grey). The donor plasmid contains a single elongation module (XUT1). Restriction results in the linearisation of the acceptor plasmid while the donor plasmid's XUT DNA fragment (bordeaux red) is extracted. The final assembled plasmid containing the assembled *NRPS* is created upon ligation of both fragments. b) After the GGA, the plasmids are transformed into *E. coli* DH10B::*mtaA* for protein expression and peptide production, followed by analysis using HPLC/MS (Figure 2b was created with BioRender).

In initial experiments, acceptor plasmids were constructed that contain a starter and a termination module. For the incorporation of elongation modules, the acceptor plasmid contains two BsaI restriction sites within the XUT^IV^ fusion site, separated by a short spacer sequence (Figure S3), all being part of the *L*‐arabinose inducible expression plasmid pACYC. BsaI restriction creates the specific overhangs A and B (Figures S2B and S3) for incorporating one or more elongation units (Figure 1a). Similarly, donor plasmids contain an elongation module and the BsaI restriction sites at both ends of the XUT sequence (Figure S3), cloned into the high‐copy plasmid pSEVA681.^[^ ^28^, ^29^ ^]^ Additionally, since GGA is a restriction enzyme‐based approach, all native BsaI sites must be removed from all acceptors and donors via the introduction of silent mutations that do not change the amino acid sequence. During the GGA reaction, the acceptor is linearised, while the XUT fragment from the donor is obtained from BsaI digestion. Subsequent ligation results in the insertion of the XUT gene fragment into the acceptor plasmid, thereby creating a single gene encoding for a three‐modular NRPS (Figure 1a). The assembled plasmids were then transformed into the producing strain *Escherichia coli* DH10B::*mtaA*,^[^ ^18^ ^]^ which were cultivated at production conditions with the required inducer(s) (Figure 1b). The resulting products were analysed using high‐performance liquid chromatography mass spectrometry (HPLC/MS).

### Elongation Module Library

Our GGA‐based strategy modifies the central motif with a few silent mutations to create the respective overhangs (Figures S2A and S4B). In order to test the influence of this minor modification on the peptide production, a set of five NRPS systems designated NRPS‐A1‐D1 to ‐D3 and NRPS‐A2_D1 to ‐D2 were cloned with GGA and NRPS‐1—NRPS‐5 with Gibson Assembly (Figure S4). To this end, two acceptor plasmids, NRPS‐A1 and NRPS‐A2, and three donor plasmids, D1, D2, and D3, were created. Both acceptor plasmids contain the NRPS starter module from the xenolindicin synthethase (XldS) from *Xenorhabdus indica* ^[^ ^18^ ^]^ that mainly incorporates a C14 fatty acid at the N‐terminus, followed by a glutamine, providing reasonable ionisation and retention times even for smaller peptides in our HPLC/MS system. The termination modules differ in both systems, whereas NRPS_A1 contains the termination module from the szentiamide synthetase (SzeS) from *X. szentirmaii*,^[^ ^30^ ^]^ incorporating a tryptophan. The second acceptor, NRPS_A2, was designed with the termination module from the GameXPeptide synthetase (GxpS) from *Photorhabdus laumondii* TTO1,^[^ ^31^ ^]^ containing a C/E domain compared to NRPS_A1 and incorporating a leucine. The modules for the first donor subset were chosen from well‐studied BGCs, incorporating AAs that are easy to detect in the downstream analysis.

Both versions of each of the five constructs cloned by Gibson Assembly (*NRPS‐1 *– *NRPS‐5*) and GGA (*NRPS‐A1_D1* to ‐*D3* & *NRPS‐A2_D1* to *‐D2*) (Figure S4A) were transformed and expressed in *E. coli* DH10B::*mtaA* for peptide production. After analysis by HPLC/MS, almost identical production profiles were obtained for both approaches, establishing the viability of GGA for NRPS engineering and the creation of libraries (Tables S7 and S8, Figure S5).

**Figure 2:**
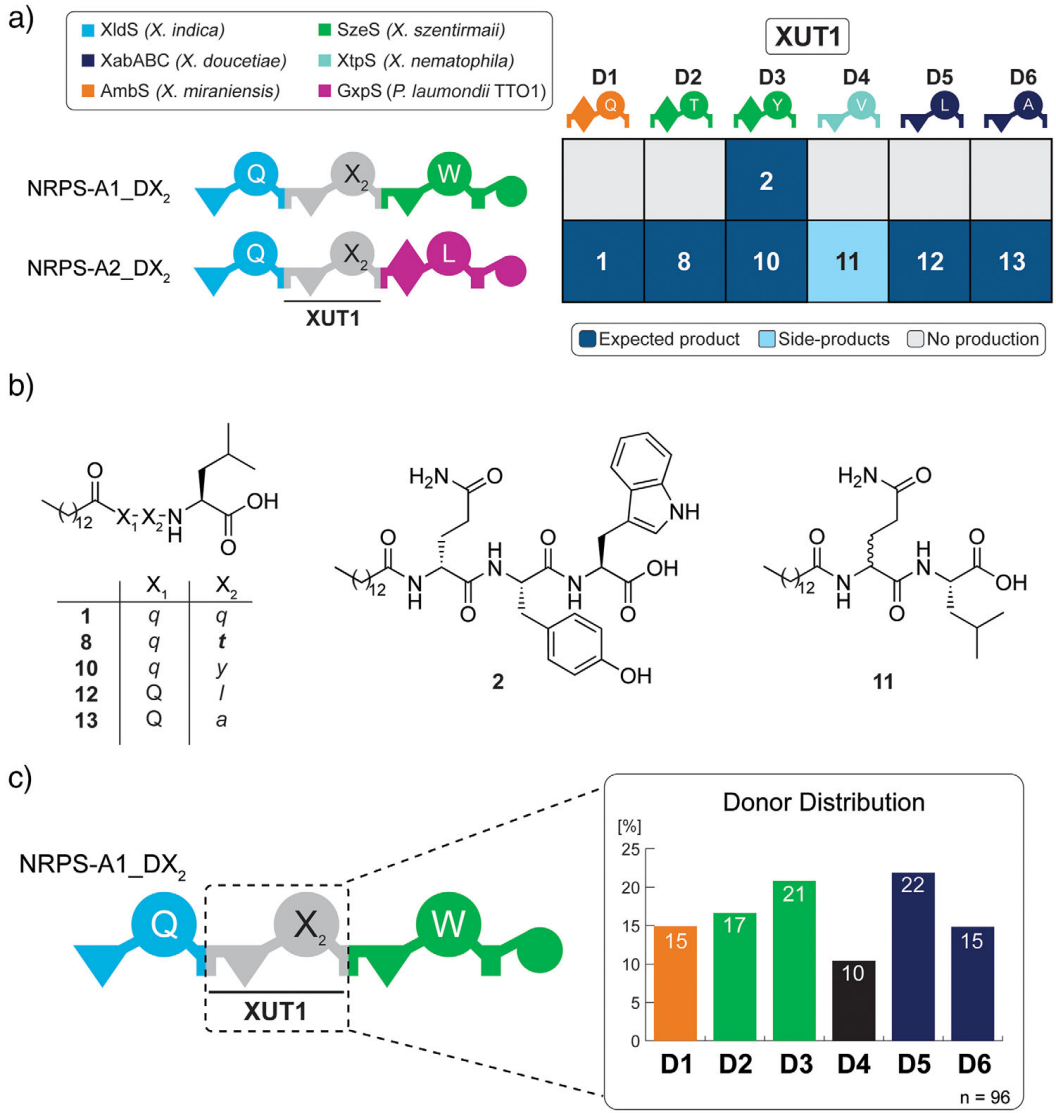
Schematic representation of the results obtained for the initial GGA NRPS module donor and acceptor subset. a) Schematic representation of NRPS encoded by the acceptor plasmids and donor plasmids with the overhangs A/B. The NRPS name and origin of the modules are depicted in a legend. A domain specificities are indicated by the one‐letter AA code. Representation of the production results from the HPLC/MS analysis of the twelve *NRPS* constructs expressed in *E. coli* DH10B::*mtaA*, where dark blue shows the presence of the expected peptide, light blue shows the presence of side product(s), and grey shows no production. Numbers within tiles refer to the produced peptides (see B). b) Chemical structures of the produced peptide sequences from the library are shown in A. AAs are shown in the one‐letter code (X_1_ for glutamine and X_2_ for the incorporated AAs). Small‐italic letters indicate *D*‐amino acids, and small‐bold‐italic letters represent *D*‐*allo*‐amino acids. c) Donor incorporation frequency determined by sequencing the one‐tube GGA approach for the encoded XUTs of the donors D1‐D6 in *NRPS‐A1* in a randomised library approach. For NRPS domain explanations, see Figure 1.

To advance the GGA concept for the assembly of an NRPS library, the number of donors was increased to six (D1‐D6), representing three modules containing C domains and three with C/E domains, and the overhangs A and B (Figure 2a, Figure S2). The HPLC/MS analysis revealed three different results for the obtained extracts from the expression of the different NRPS systems: i) The expected peptide was detected, as shown for NRPS‐A2_D6 producing C14‐Q*a*L **13** (*D*‐amino acids are shown with small letters and in italics; Figure S6). ii) Only other derivatives or peptides resulting from module skipping were detected, as shown for NRPS‐A2_D4, producing the truncated peptide C14‐*q*L **11** (Figure S7). iii) No peptide was detected at all. Most of the NRPS‐A2_DX systems show the production of the expected peptide, while for NRPS‐A1_DX, only NRPS‐A1_D3 produced the expected peptide (Figure 2a,b). In addition, the applicability of the GGA‐based NRPS engineering for the generation of a fully randomised NRPS library was tested with the donor set D1–D6 in combination with NRPS‐A1. When an equal amount of all donor fragments was used for GGA and transformed into *E. coli*, an almost equal distribution of possible NRPS variants was detected (Figure 2c).

**Figure 3:**
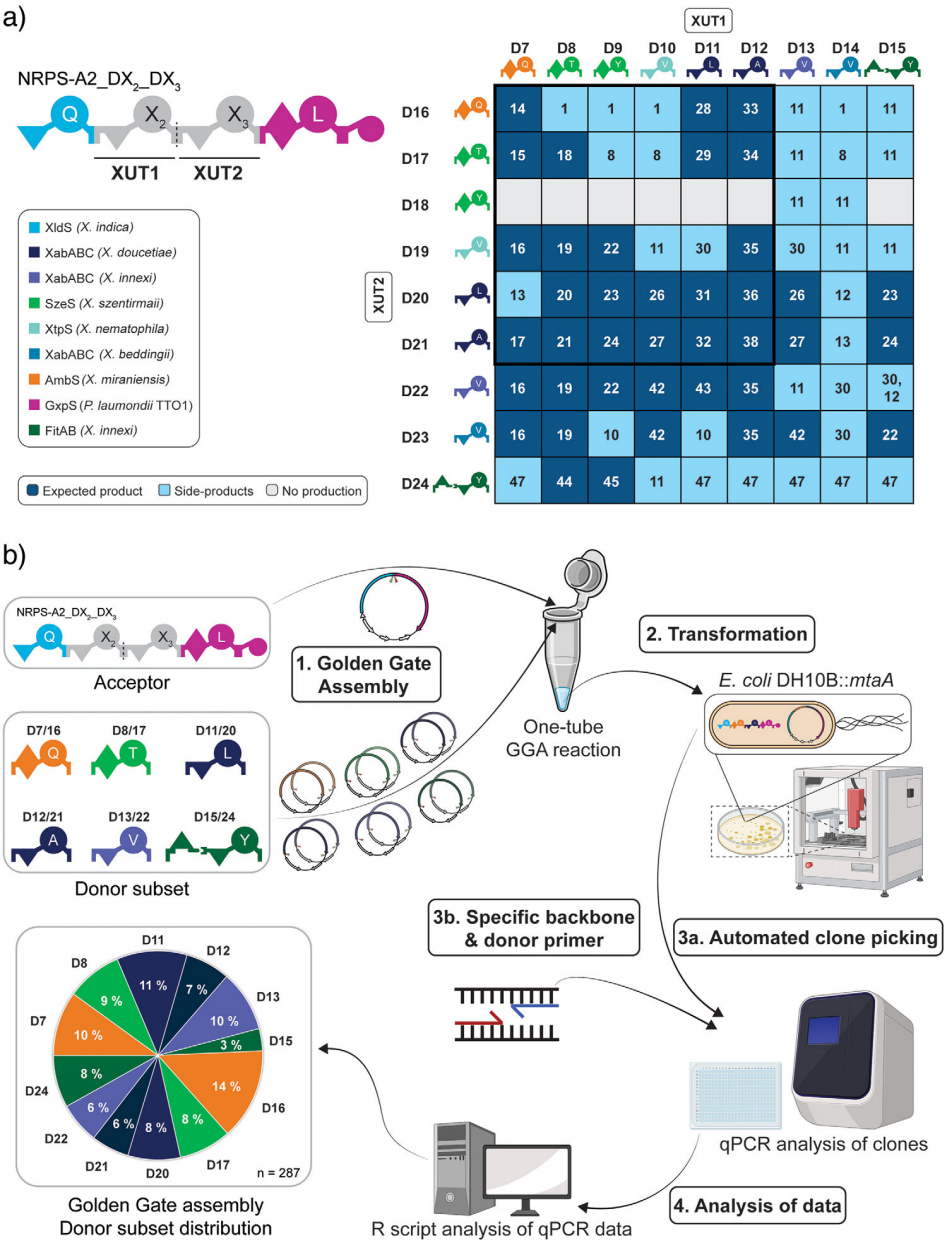
Overview of the lipo tetrapeptide library and the semi‐automatic workflow of the qPCR‐based NRPS‐encoding plasmid validation. a) Peptide production as determined by HPLC/MS analysis of the 81 NRPS constructs of the tetra‐modular library, where XUT1 and XUT2 have been varied by usage of donor plasmid D7‐D15 and D16‐D24, respectively. The black box highlights the first set of 36 constructed using D7‐D12 and D16‐D21. Dark blue, light blue, and grey tiles represent the detection of the expected peptides (see Figure 4), peptide derivatives (see Table S7), and no peptide production, respectively. Numbers within tiles refer to the produced peptides (see Figure 4, Table S7). The origin of the different modules is indicated by colour. b) Workflow of the semi‐automated qPCR‐based NRPS plasmid validation for the distribution analysis of the modified donor subset. For NRPS domain explanations, see Figure 1 (Figure 3b was created with BioRender).

**Figure 4:**
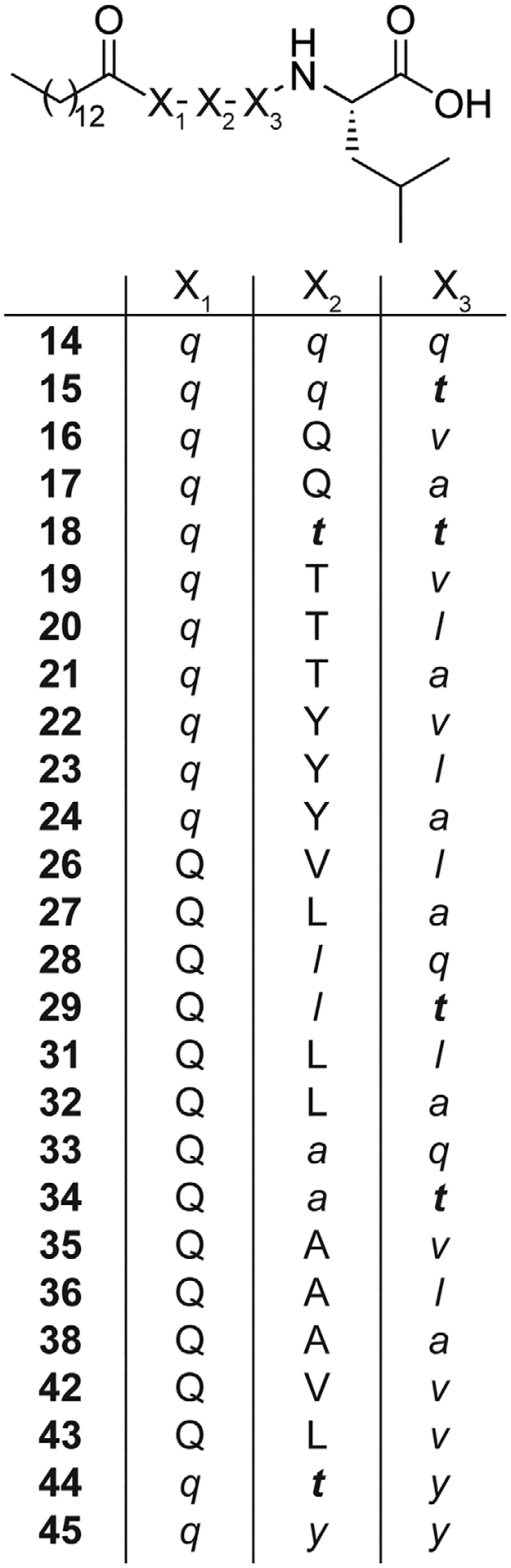
Chemical structures of all identified expected peptides produced by the NRPS systems are shown in Figure 3a. The AA “X_1_” (Gln, Q) can have either *L*‐ and *D*‐configuration, dependent on the C or C/E domain preceding the A domain of the inserted XUT. “X_2_” and “X_3_” describe the AAs in the first and second incorporated XUT. *D*‐amino acids are shown in small and italic letters, and *D*‐*allo*‐Thr is shown in small, italic, and bold letters.

In order to test whether multiple modules can be incorporated at different positions of the NRPS, the subset of donors (Figure 2a) containing the overhangs A and B was cloned as novel donors comprising the overhangs A/C (D7‐D12) and C/B (D16‐21). This approach facilitates the implementation of two donor fragments, thus creating 36 distinct NRPS systems, which were analysed as previously described (Figure 3a, black box). HPLC/MS analysis revealed that 83% of the construct produced functional NRPS, with 61% of them indeed producing the expected peptide (Figures 3a and 4). The expression of some *NRPS*, such as *NRPS‐A2_D7_D21*, produced not only the expected peptide **17** but also truncated versions such as **13** and **11**, originating again from module skipping (Figure S8). As an example of a functional NRPS not producing the expected peptide, NRPS‐A2_D9_D16 only produced the truncated peptides **1** and **11** (Figure S9). Unfortunately, the tyrosine incorporating module from D18 mainly resulted in non‐functional NPRS (Figure 3a). Furthermore, the valine incorporating D10 module primarily yielded NRPS systems where the expected peptide could not be detected, consistent with the earlier findings from the single‐module library (Figures 2a and 3a). Therefore, additional valine and tyrosine modules were used and added as donors with A/C (D13‐D15) and C/B (D22‐D24) overhangs. This resulted in the construction of 81 different NRPS constructs in total, showing an increase in functional NRPS as well as the detection of the expected peptide sequences (Figure 3a).

All results regarding the produced peptides are summarised in Table S7, showing each construct with the detected peptides. Generally, all functional NRPS produced the peptides C14‐QL and C14‐*q*L **11** (Figures S7–S9), which only contained the building blocks from the acceptor plasmid. Therefore, **11** was only mentioned explicitly in Figure 3a when no other derivatives were detected. Furthermore, in some NRPS systems, two peaks with the same *m*/*z* ratio were detected, indicating incomplete epimerisation by the C/E domain of the terminal module as a result of the engineered NRPS (Figures S7 and S9). In addition to HPLC/MS analysis, compounds **13** and **36** were chemically synthesised as examples to confirm their structure (Figures S10 and S11).

Similar to the single‐module library, the distribution of the incorporated modules was again tested to confirm equal distribution within a library approach. The new valine (D13 and D22) and tyrosine (D15 and D24) modules were used in place of the lesser‐performing ones (D9 and D18; D10 and D19) (Figure 3b, Figure S12). In order to simplify the analysis process independent of full plasmid or possible donor sequencing, an end‐point qPCR‐based analysis was developed (Supporting Information Part B) to investigate the incorporated fragment and its position (see Supporting Information D). For this analysis, 287 random clones were selected and tested for their plasmid composition (Figure 3b). Although D16 was slightly overrepresented at 14% and D15 underrepresented at 3.5%, most of the donors used show a distribution of 8%–9%. This shows that this approach can be used to efficiently generate randomised libraries, while specific colonies can be analysed, as shown above.

### Starter Module Library

**Figure 5:**
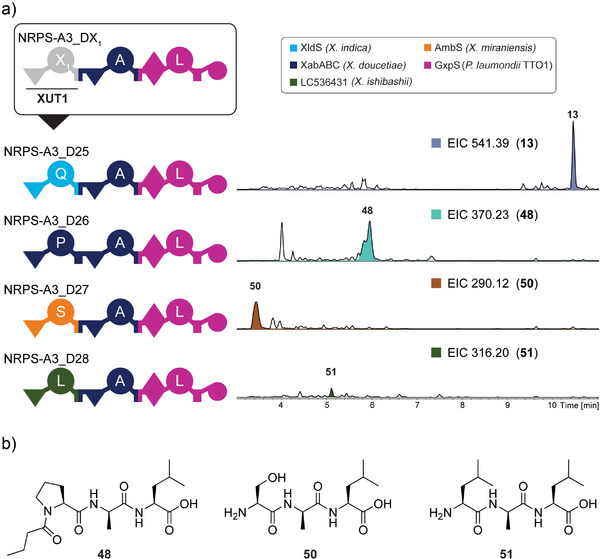
Schematic representation of the starter module system for GGA‐based engineered NRPS. a) Schematic representation of NRPS‐A3 and NRPS‐A3_D25 to D28 with the colour code of the original NRPS. The A domain specificities are indicated by the one‐letter AA code. The final plasmids were assembled via GGA using the acceptor plasmid encoding NRPS‐A3, lacking a starter module, and the donors D25‐D28. HPLC/MS data of compounds **13**, **48**, **50**, and **51** produced in *E. coli* DH10B::*mtaA* expressing *NRPS‐A3_D25* to *_D28*. Base peak chromatograms (BPC, black lines) and the extracted ion chromatogram (EIC) of **13** (*m/z* [M + H]^+^ = 541.39), **48** (*m/z* [M + H]^+^ = 370.23), **50** (*m/z* [M + H]^+^ = 290.12) and **51** (*m/z* [M + H]^+^ = 316.20) are shown. b) Chemical structures of the compounds **48**, **50**, and **51**. For NRPS domain explanations, see Figure 1.

Following the success of utilising GGA to incorporate elongation modules, the method should be expanded to starter modules, allowing the attachment of different N‐termini to the produced peptide. The starter donor was designed to be analogous to the elongation donors, containing the A overhang at the downstream end to ensure compatibility with the elongation module library (e.g., overhangs A/B). The upstream overhang was designed using the start codon “ATG”. Given the requirement of four bases, the nucleotide in front of the start codon (xATG) was utilised in addition to prevent alterations to the subsequent AAs of the expressed *NRPS*. Consequently, the overhang S (“starter”; CATG) (Figure S2B) and overhang A were employed to incorporate starter modules from donors (i.e., overhangs S/A for starter modules), exhibiting a design compatible with the previously described elongation donors (e.g., S/A + A/B or S/A + A/C + C/B). Regarding the new acceptor NRPS‐A3, one of the best‐producing constructs from the elongation library, designated as NRPS‐A2_D6, was used (Figure 5). The starter module was removed, and BsaI restriction sites were introduced in a manner similar to that used for NRPS‐A1 and NRPS‐A2. The D25 starter (C14‐Q) was used as the positive control. The additional starter modules originate from the xenoamicin‐producing NRPS (XabABC) (D26; C4‐P) from *X. doucetiae*,^[^ ^32^ ^]^ the ambactin‐producing NRPS (AmbS) (D27; S) from *X. indica* having a non‐functional C_starter_ domain,^[^ ^18^ ^]^ and a previously uncharacterised starter from an unknown NRPS‐encoding gene cluster from the *X. ishibashii* LC536431 (Figure 5). The extracts of the four expressed *NRPS* (*NRPS‐A3_D25* to ‐*D28*) were analysed using HPLC/MS, showing the production of the expected products (Figure 5b). Both NRPS‐A3_D27 and NRPS‐A3_D28 produced peptides (**50**, **51**) without any acyl group at the N‐terminus, suggesting the C_starter_ domain of LC536431 is also non‐functional in regards to the incorporation of an acyl moiety. For further confirmation of the structure, compound **50** was synthesised chemically, confirming the NRPS‐produced compound (Figure S13). The success of using starter modules for the GGA approach increases the combinatorial possibilities as well as the ability to modify the N‐terminus of peptides.

### Construction of the Xenoamicin Library Using the GGA Approach

**Figure 6:**
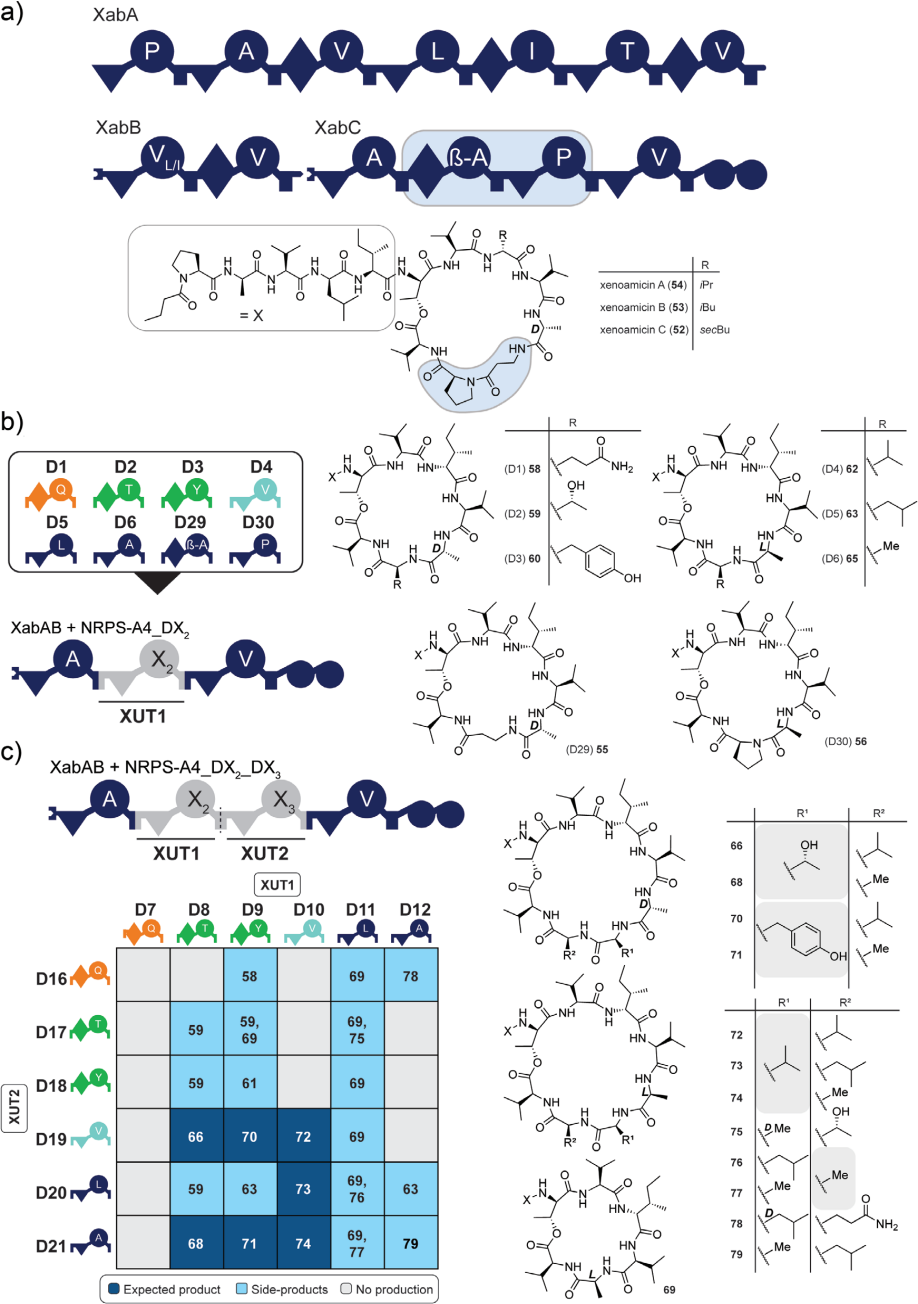
Overview of the xenoamicin library results using replacements of one or two XUTs. a) Overview of the XabABC NRPS architecture and the chemical structure of its main products, xenoamicin A–C (**52**–**54**). The exchanged modules and AAs are marked in light blue within the NPRS and the chemical structure, respectively. b) Incorporation of single modules from the first donor subset (D1‐D6), D29, and D30, all resulting in the production of the expected peptide. The linear N‐terminal region is abbreviated as “X”. c) Summary of the HPLC/MS analysis results from the extracts of 36 NRPS constructs of the xenoamicin library containing two exchanged XUTs. Dark blue, light blue, and grey tiles represent the detection of expected peptides, peptide derivatives, and no peptide production. Numbers within tiles refer to the compounds (Table S9). For the explanation of the NRPS domain origin of the inserted XUTs according to their colour, see Figure 3. For NRPS domain explanations, see Figure 1.

In order to test GGA for the derivatisation of a natural NRPS system, we aimed to modify the NRPS XabABC^[^ ^32^ ^]^ (Figure 6a), producing xenoamicins A‐C (**52**–**54**) as main products in the heterologous host *E. coli* DH10B::*mtaA*. Xenoamicins are 13‐amino acid‐long lipo‐depsipeptides with an acylated linear N‐terminal and a cyclic C‐terminal part, created by an intramolecular cyclisation from the threonine‐8 hydroxyl group to the C‐terminal carboxyl group of valine‐13. The primary objective was to change the AA composition within the ring structure via modification of XabC, which incorporates the last four AAs (Figure 6a). Additionally, *xabD*, encoding for an aspartate decarboxylase that is located downstream of *xabC*, is required for the synthesis of β‐alanine (β‐A). For simplicity reasons, the genes for *xabA* and *xabB* were cloned on a separate pCOLA plasmid for co‐expression. The acceptor NRPS‐A4 contains the first and last module from XabC, lacking the modules for β‐alanine and proline, which can be reintroduced as donor building blocks. BsaI restriction sites, similar to NRPS‐A1 and NRPS‐A2, were added, creating A/B overhangs. In addition to the modified NRPS, *xabD*, coding for the aspartate decarboxylase, was cloned on the acceptor plasmid for the required synthesis of β‐alanine.

In the first experiments, donors D1‐D6, as well as the β‐alanine (D29) and proline donor (D30), were incorporated into NRPS‐A4_DX. The HPLC/MS analysis of the obtained culture extracts showed that all eight NRPS were functional, producing the expected peptides (**55**–**65**) (Figure 6b, Table S9). Since the first XUT might carry a C/E or a C domain, the Ala at position 10 might be *D*‐ (as in the original xenoamicins) or *L*‐configurated, respectively.

Furthermore, the native NRPS produced the two derivatives with the same *m*/*z*, xenoamicin C (**52**) and xenoamicin B (**53**), having an identical mass but differing in the incorporation of isoleucine or leucine at position eight (Figure S14). From a previous work,^[^ ^32^ ^]^ we postulate that the peak eluting later is the isoleucine‐containing main derivative xenoamicin C (**52** Figure S14); therefore, Ile‐8‐containing structures are presented for simplicity (Table S9).

In addition to all constructs being functional, the ring size of the depsipeptide was reduced since two modules from the original NRPS were exchanged against one donor module. Additionally, along with the expected depsipeptides, linear peptides were also detected (Figure S15).

When two modules using D7‐D12 and D16‐D21 were incorporated together, peptides with the original number of AAs should be obtained. As a positive control, the parent *NRPS‐A4_D31_D34*, using D31 and D34, was generated first, showing comparable production titers to those of the native XabABC under these conditions (Figure S14). The structure of compound **71** was additionally synthesised chemically, confirming the expected structure produced by NRPS‐A4_D9_D21 (Figure S16). From the 36 generated xenoamicin synthetase variants, eight showed the production of the expected peptides, while 16 showed skipping of one or both of the newly incorporated modules, respectively, and 13 did not show any production (Figure 6c). Of the total 23 peptides produced, 17 represent new derivatives of xenoamicin, highlighting the power of the GGA for NRPS engineering.

## Conclusion

NRPS are among the largest enzymes known, and their size and modularity are both a difficulty and a chance for manipulation in order to obtain new‐to‐nature peptides.^[^ ^2^, ^33^, ^34^ ^]^ Although the size of the proteins and their associated gene sizes (∼3 kb per module) can be addressed via the introduction of natural^[^ ^35^, ^36^ ^]^ or artificial docking domains,^[^ ^20^, ^37^, ^38^ ^]^ which enables simplified cloning of smaller genes on suitable expression plasmids, the recently described XUT approach allows an efficient exchange, insertion or deletion of NRPS parts within a single given NRPS protein.^[^ ^14^ ^]^

In this work, we successfully establish a new GGA syntax and workflow to leverage the cloning of artificial hybrid NRPS genes via XUT engineering. Compared to the previously applied Gibson Assembly, the new approach allows the rapid assembly and verification of defined artificial NRPS, as well as entire defined and randomised artificial NRPS libraries (Figures 2 and 3). Once generated, donor plasmids can be used for various XUT‐based engineering applications like variation of single or multiple sites within non‐ribosomal peptides, as demonstrated for the xenoamicin in this work (Figure 6). In our opinion, the minor disadvantage of eliminating restriction sites such as BsaI when engineering targeted NRPS‐encoding genes is clearly outweighed by the advantages. However, despite the structural similarity between NRPS and PKS T domains, the conserved FFxxGGxS motif is not as widespread in PKS T domains as it is in NRPS, thus limiting this approach to NRPS T domains at the present time.^[^ ^14^, ^39^, ^40^ ^]^ With respect to the overall outcome, our approach is similar to protein engineering approaches^[^ ^41^ ^]^ where individual AA positions can easily be exchanged or randomised based on DNA‐sequence variation. However, the large variability of available NRPS‐encoded AA building blocks^[^ ^2^, ^42^, ^43^ ^]^ offers access to a much greater chemical space than the 20 proteinogenic AAs. The flexible application of the GGA‐based NRPS engineering comes with greater sustainability in terms of workload and consumables and has the potential to derivatise multiple non‐ribosomal peptides in high‐throughput to identify novel bioactive peptides.

The increased throughput of the presented workflow also allows the acquisition of larger datasets that contain different NRPS hybrids from various genera of bacteria with functional and non‐functional NRPS assemblies. Analysis by machine learning algorithms might result in the identification of rules for acceptor–donor or donor–donor functionality and/or peptide production success, which could be dependent on so far unknown interactions of domains, linkers, or protein interfaces.

In conclusion, the established GGA‐based cloning for XUT engineering represents a significant improvement in developing and applying NRPS engineering in high‐throughput.

## Author Contributions

A.P. performed all experiments except for the qPCR‐based NRPS plasmid validation experiments. A.P., H.M., and L.P. developed the Golden Gate overhangs, and the development of the qPCR method was done by T.A.L., D.S., and A.P. The qPCR‐related experiments were done by A.P. and T.A.L. The custom R script was written by T.A.L. The chemical standards were synthesised by M.D. The analysis of the conserved XUT motif was performed by L.P. H.B.B. supervised the work, and the paper was written by A.P. with contributions from all authors.

## Conflict of Interests

The authors declare no conflict of interest.

## Supporting information



Supporting Information

Supporting Information

## Data Availability

All data and materials can be found within the manuscript, Supporting Information, or requested from the corresponding author.
