# Supplementary material for: High‐Throughput Engineering and Modification of Non‐Ribosomal Peptide Synthetases Based on Golden Gate Assembly
Source: Angew Chem Int Ed Engl. 2025 Oct 11;64(49):e202508967. doi: 10.1002/anie.202508967 (PMC12668312; doi:10.1002/anie.202508967)
Supplement: Supplementary file 1 — Supporting Information [file ANIE-64-e202508967-s001.pdf]

Supporting Information for

High-Throughput Engineering and Modification of Non-ribosomal Peptide Synthetases Based on Golden Gate Assembly

Adrian Podolski[1,2], Timon A. Lindeboom[1,3], Leonard Präve[1,2], Maryam Dehghan[1], Hannah A. Minas[1,6], Janik Kranz[2], Daniel Schindler[1,3,†], Helge B. Bode[1,2,4,5,*]

1. Max Planck Institute for Terrestrial Microbiology, Department of Natural Products in Organismic Interactions, 35043, Marburg, Germany
2. Molecular Biotechnology, Department of Biosciences, Goethe University Frankfurt, 60438, Frankfurt am Main, Germany
3. MaxGENESYS Biofoundry, Max Planck Institute for Terrestrial Microbiology, 35043, Marburg, Germany
4. Center for Synthetic Microbiology (SYNMIKRO), Phillips University Marburg, 35043 Marburg, Germany
5. Department of Chemistry, Phillips University Marburg, 35043 Marburg, Germany
6. Myria Biosciences AG, Hochbergerstrasse 60 C, 4057 Basel, Switzerland

† Current address: Center for Molecular Biology of Heidelberg University (ZMBH),
69120 Heidelberg, Germany

* Corresponding author: helge.bode@mpi-marburg.mpg.de

A. Materials and Methods

1. Cultivation of strains

All *E. coli* DH10B::*mtaA* were cultured on liquid or solid low-salt LB medium (pH 7.5, 10 g/L tryptone, 5 g/L yeast extract and 5 g/L NaCl). As a selection marker, either chloramphenicol (34 µg/mL), kanamycin (50 µg/mL) or gentamicin (20 µg/mL) was added. Solid medium was prepared with 1% agar (w/v). The cells were cultivated at 37 °C. All *Xenorhabdus* strains were cultivated on solid low-salt LB medium (pH 7.5, 10 g/L tryptone, 5 g/L yeast extract and 5 g/L NaCl, 1% agar (w/v)). The cells were cultivated at 28 °C.

2. Cloning of biosynthetic gene clusters and NRPS acceptors and donors

Genomic DNA (gDNA) was isolated via Monarch® Genomic DNA Purification Kit (NEB) from the respective strains depicted in Table S1 for usage as a template for PCR amplification. Alternatively, plasmid DNA was used as a template, especially for the re-amplification of donor plasmid fragments with different overhangs. In this work, Q5® High-Fidelity DNA Polymerase (NEB) or Phusion® Hot Start Flex DNA Polymerase (NEB) was utilised to create all DNA fragments. Primer pair sequences and products are summarised in Table S4. The PCR amplified fragments were digested with DpnI (NEB) (manufacturer instructions) and purified by gel extraction from 1% (w/v) agarose gels using the Monarch® DNA Gel Extraction Kit (NEB).

The acceptor and donor plasmids were cloned using Gibson assembly from the NEBuilder® HiFi DNA Assembly Cloning Kit (NEB). Additionally, native BsaI restriction sites are removed by point mutagenesis. The final constructs were assembled using Golden Gate Assembly from NEBridge® Golden Gate Assembly Kit (NEB) (reaction according to manufacturer's instructions) with the acceptor and donor plasmids, using the protocol for multiple fragments ((5 min 37 °C → 5 min 16 °C) x 30 cycles followed by 5 min 60 °C). Gibson and Golden Gate assembled plasmids were transformed by heat shock into chemical-competent *E. coli* DH10B::*mtaA*. Plasmids were isolated using the Monarch® Plasmid Miniprep Kit (NEB).

3. Heterologous expression of NRPS constructs

After plasmid transformation via heat shock into chemically competent *E. coli* DH10B::*mtaA*, cells were cultivated overnight in LB medium containing the necessary antibiotics. 24-deep well plates with 4 mL XPP medium[1] (each well) containing the

required antibiotics and 0.02% *L*-arabinose (w/v) were inoculated with 1% overnight-grown culture. The cells were cultivated at 22 °C for 72 hours at 200 rpm.

4. Culture extraction and HPLC/MS analysis

The cultures were extracted by mixing the culture in a 1:1 ratio with methanol, followed by a 1 h incubation at room temperature while shaking. The deep-well plates were centrifuged for 30 min at 22 °C and 4400 x g. Extracts in reaction tubes were centrifuged for 20 min at 22 °C and 17000 x g.

Cleared supernatant was further used for high-performance liquid chromatography mass spectrometry (HPLC/MS) and high-performance liquid chromatography high-resolution mass spectrometry (HPLC/HRMS) analysis. For HPLC/MS, the analysis was performed using Agilent Infinity II coupled to an AmaZon speed ETD spectrometer (Bruker) with an ACQUITY UPLC BEH C18 column (130 Å, 2.1 mm × 100 mm, 1.7-µm particle size, Waters) at a flow rate of 0.4 mL/min (5–95% acetonitrile/water with 0.1% formic acid, vol/vol, 16 min, and an electrospray ionisation (ESI) source set to positive ionisation mode. To obtain high-resolution masses, HPLC/HRMS analysis was performed using performed using Bruker Elute coupled to an timsTOF flex MALDI-2 spectrometer (Bruker) with an ACQUITY UPLC BEH C18 column (130 Å, 2.1 mm × 100 mm, 1.7-µm particle size, Waters) at a flow rate of 0.4 mL/min (5–95% acetonitrile/water with 0.1% formic acid, vol/vol, 16 min, and a VIP-HESI (ESI) source set to positive ionisation mode. The MS data was analysed using DataAnalysis 6.1 (Bruker). Additionally, the peptide masses and sequences were calculated using Pep-Calc API[2].

5. Golden Gate overhangs

The described overhangs in this work are all the 16 possible overhangs for this fusion site using the codons of both glycines (Fig. S1). However, not all of them show perfect compatibility with each other, leading to potential mismatches during the GGA. Systems such as NEBridge Ligase Fidelity Viewer[3–5] offer excellent analysis capabilities for viewing fidelities and identifying possible mismatches, allowing for adjustments to the used overhangs accordingly.

6. Determination of the glycine motif conservation within the central motif

The analysis was performed using Python on the previously published dataset of 130870 amino acid sequences of T domains derived from bacteria, which was

extracted from the antiSMASH database.[6] First, all sequences were searched for the conserved FFxxGGxS motif utilising Python with variable search strings (Regular expression module). The amino acid sequence and the central motif were stored in a dataframe (Pandas module). Next, amino acid sequences with fewer than 65 or more than 80 amino acids were excluded from the dataframe. Subsequently, all remaining sequences with no identified central motif were excluded from the analysis, resulting in 127467 sequences suitable for analysis. Finally, the central motif was analysed by counting the occurrences of the motif at positions 5 and 6 for each central motif in the dataframe (using the Numpy and Counter modules). The percentage of the counts was calculated and plotted in a bar plot (Matplotlib module).

7. qPCR analysis for NRPS constructs

Candidates were picked from a 150-mm 2% LB agar Petri dish using the Singer PIXL colony picker onto a 96 SBS-format agar plate. Following an overnight growth period at 37 °C, the colonies were transferred to 50 µL of sterile water. qPCR primers were designed using the Primer3 software[7]. Subsequently, 25 nL of 10 µM premixed primers and 100 nL of the resuspended colonies were transferred to 384 qPCR plates (Sarstedt, 72.1984.202) using the Echo525 acoustic liquid dispenser (Labcyte), and analysis was conducted on the same day. 1 µL of 1X Luna Universal qPCR Master Mix (NEB) was dispensed using a nanoliter dispenser (Cobra, Art Robbins Instruments). Subsequently, the plates were subjected to a brief centrifugation step before being sealed with an optical clear permanent seal (Agilent, 24212-001) using the Plate Loc Thermal Microplate Sealer (Agilent) at 180 °C for 1 s. The Applied Biosystems QuantStudio 5 was then utilised for SYBR Green detection of the prepared plates. Samples were first subjected to a preincubation step at 50 °C (1.6 °C/s) for 2 minutes, followed by a 1-minute incubation at 95 °C (2.57 °C/s). Subsequently, a PCR reaction was performed, comprising 30 cycles each at 95 °C (2.57 °C/s) for 1 s and 67 °C (2 °C/s) for 1 min, with a single acquisition. A melting curve was acquired from 97 °C (0.1 °C/s) with continuous acquisition. The resulting data was exported as an Excel file and subsequently analysed using a custom R script (see **A.8**).

8. Custom R script for the qPCR analysis

Found in a separate SI

9. Chemical peptide synthesis

Peptides **13**, **36**, **50** and **71** were synthesised using a microwave-assisted peptide synthesiser (Liberty Prime 2.0 from CEM) with an HT12 high-throughput loader. The 9-fluorenylmethoxycarbonyl (Fmoc) strategy was used with *N*,*N*'-diisopropylcarbodiimide/ethyl 2-cyano-2-(hydroxyimino) acetate (DIC/Oxyma). The Wang-resin was preloaded and used for a 100 μmol scale. The resin is transported from the HT12 loader to the reactor vessel using dimethylformamide (DMF) and a nitrogen gas flow. Amino acid coupling, deprotection, and washing are performed in a step-wise cycle. For coupling, 1 mL of amino acid solution (0.5 mM in DMF), 1 mL of DIC (2 M in DMF), and 1.5 mL of an Oxyma/diisopropylethylamine (DIPEA) (0.25 M/0.1 M in DMF) solution are incubated at 105 °C and under a nitrogen atmosphere for 1 min. After washing with DMF, the Fmoc protection group is deprotected using 0.75 mL of pyrrolidine (25% v/v in DMF) and incubated at 110 °C for 40 s. This cycle is repeated until all amino acids are attached to the peptide chain. The final peptide is cleaved from the resin using 5 mL of a TFA/H_2O/TIS solution (95:2.5:2.5 v/v), which is incubated for 1 h.

For the cyclisation of peptide **71**, 1 equivalent (eq) (0.05 mmol) was dissolved in 50 mL of dichloromethane (DCM) and kept under a nitrogen atmosphere. The reaction was stirred while 7.5 equivalents (eq) of 2-mesyl-6-nitro-1,2,3-benzothiadiazole (MSNT; 119 mg), 5 eq of *N*-methylimidazole (NMI; 20 μL), and 12 eq of DIPEA (105 μL) were added sequentially. The reaction was stirred at room temperature for 24 h. Thereafter, the solvent was removed under reduced pressure conditions and purified using a preparative HPLC (1260 Infinity II Preparative LC/MSD; Agilent) with a C_{18} column (Eclipse XDB-C18 (21.2 x 250 mm, 7 μm); Agilent).

B. Supplementary method and results: Results of the end-point qPCR-based NRPS plasmid validation used for the tetra-modular NRPS library

During the validation of the libraries, Sanger sequencing confirmed the incorporation of one module during GGA, but it is not suited for sequencing two or more modules. This is due to the fact that NRPS XUTs exceed the size of traditional Sanger sequencing reads (~800 bp) and would, therefore, require multiple repetitions. Alternatively, Nanopore sequencing enables whole plasmid sequencing based on its long-read technology. However, these methods require the cultivation of each strain of the library, as well as the extraction and purification of DNA (i.e. the plasmid). Since

known DNA sequences were used as a predefined set of donors, we developed an end-point quantitative polymerase chain reaction (qPCR) method[8] to validate the GGA-generated NRPS libraries, which utilises *E. coli* colonies as templates and eliminates the need for extensive liquid cultivation, DNA extraction, and purification. Furthermore, in comparison to regular PCR, qPCR eliminates the need for gel electrophoresis and subsequently allows for the minimisation of reactions to 1 µL, saving materials.

For qPCR, two specific primers were designed for each donor, one for the starter and one for the termination module on the acceptor plasmid (Fig. S12 and Table S5). The forward primers are located at the end of the A domain, whereas the reverse primers are located within the T-C linker or at the beginning of the C domain. This results in multiple primer combinations for each possible XUT position within the GG-assembled NRPS constructs. For the detection of the correct XUT and its position, each primer combination was used for each template. Optimally, only two qPCR reactions show a positive result, creating the expected amplicon (~450-600 bp). The primers were first tested on purified plasmid DNA to record the melting temperature (T_m) and the size of each amplicon. Following successful results, the system was applied to *E. coli* cells (see **A.7**), where qPCR amplifications can be easily traced back to the original colony. The tetra-modular library was tested by examining 288 colonies (Fig. 3B) (287 of which contained the library and one of which included an empty plasmid). The C_t cut-off was placed at 18 cycles, and the minimum T_m at 80 °C. This analysis determined that five of the colonies were false negatives, as they failed to produce two signals. Furthermore, nine showed signals for two separate plasmids. Re-evaluation of the original colony agar plates revealed that during colony-picking, double colonies were selected and therefore picked. Of the examined colonies, 96 were cultivated, the plasmids purified and sent for plasmid sequencing. The sequencing results were consistent with those from the qPCR, including the nine candidates that showed signals for two plasmids, confirming that two plasmids were present. With false negatives and the double-picked colonies, the success rate of this method was over 95%. This showed great efficiency in utilising minimal resources and huge time benefits, while maintaining a good success rate for the analysis of the GG-assembled NRPS constructs.

C. Supplemental Tables

Table S1. Strains used in this work.

Strain	Genotype/NRPS	Reference
E. coli DH10B::mtaA	F_mcrA (*mrr-hsd*RMS- *mcr*BC), 80*lac*ZΔ, M15, Δ*lac*X74 *rec*A1 *end*A1 *ara*D 139Δ(*ara*, *leu*)7697 *gal*U *gal*Kλ *rps*L (*Strr*) *nup*G and mtaA from pCK_*mtaA*Δ*entD* / -	[9]
P. laumondii TTO1	WT (*gxpS*[10])	DSMZ
X. indica DSM 17382	WT (*xldS*[9])	DSMZ
X. miraniensis DSM 17902	WT (ambS[9])	DSMZ
X. szentirmaii DSM 16338	WT (*szeS*[11])	DSMZ
X. nematophila ATCC 19061	WT (*xtpS*[12])	DSMZ
X. doucetiae DSM 17909	WT (*xabABCD*[13])	DSMZ
X. innexi DSM 16336	WT (*xabABC*, *fitAB*[6])	DSMZ
X. beddingii DSM 4764	WT (*xabABC*)	DSMZ
X. ishibashii DSM 22670	WT (Locus tag: LC536431)	DSMZ

Table S2. NRPS, corresponding plasmids and genotypes used in this work.

NRPS-	Plasmids	Genotype	Reference
	pCOLA_ara/tacI	ori ColA, kan^R, *araC-*P_{BAD} and *tacI*	[14]
	pACYC_ara/araE _tacI	ori p15A, cm^R, *araC-*P_{BAD}, *tacI* and *araE*	[15]
	pSEVA681	ori pUC, oriT, gm^R	[16,17]
	pAP128	ori ColA, kan^R, *araC-*P_{BAD} *xabAB* and *tacI*	This work
**A1**	pAP38	ori p15A, cm^R, *araC-*P_{BAD} *xldS*_C1A1T1$_{1/2}$_2xBsaI_*szeS*_T5$_{1/2}$C 6A6T6Te *tacI* and *araE*	This work
**A2**	pAP37	ori p15A, cm^R, *araC-*P_{BAD} *xldS*_C1A1T1$_{1/2}$_2xBsaI_*gxpS*_T4$_{1/2}$C /E5A5T5Te *tacI* and *araE*	This work
**A3**	pAP106	ori p15A, cm^R, *araC-*P_{BAD} 2xBsaI_*xabA*_T1$_{1/2}$AC2A2T2$_{1/2}$_*gxpS* _T4$_{1/2}$C/E5A5T5Te *tacI* and *araE*	This work

NRPS-	Plasmids	Genotype	Reference
**A4**	pAP119	ori p15A, cm^R, *araC-P$_{BAD}$* *xabC*_C1A1T1$_{1/2}$_2xBsaI_*xabC*_T3$_{1/2}$ C4A4T4TeTe *tacI* and *araE*	This work
**1**	pAP32	ori p15A, cm^R, *araC-P$_{BAD}$* *xldS*_C1A1T1$_{1/2}$_*szeS*_T1$_{1/2}$C/E2A2T2$_{1/2}$_*szeS*_T5$_{1/2}$C6A6T6Te *tacI* and *araE*	This work
**2**	pAP33	ori p15A, cm^R, *araC-P$_{BAD}$* *xldS*_C1A1T1$_{1/2}$_*ambS*_T1$_{1/2}$C/E2A2T2$_{1/2}$_*szeS*_T5$_{1/2}$C6A6T6Te *tacI* and *araE*	This work
**3**	pAP34	ori p15A, cm^R, *araC-P$_{BAD}$* *xldS*_C1A1T1$_{1/2}$_*szeS*_T1$_{1/2}$C/E2A2T2$_{1/2}$_*gxpS*_T4$_{1/2}$C/E5A5T5Te *tacI* and *araE*	This work
**4**	pAP35	ori p15A, cm^R, *araC-P$_{BAD}$* *xldS*_C1A1T1$_{1/2}$_*ambS*_T1$_{1/2}$C/E2A2T2$_{1/2}$_*gxpS*_T4$_{1/2}$C/E5A5T5Te *tacI* and *araE*	This work
**5**	pAP36	ori p15A, cm^R, *araC-P$_{BAD}$* *xldS*_C1A1T1$_{1/2}$_*szeS*_T4$_{1/2}$C/E5A5T5$_{1/2}$_*szeS*_T5$_{1/2}$C6A6T6Te *tacI* and *araE*	This work
**5 mod**	pAP36b	ori p15A, cm^R, *araC-P$_{BAD}$* *xldS*_C1A1T1$_{1/2}$_*szeS*_T4$_{1/2}$C/E5A5T5$_{1/2}$_*szeS*_T5$_{1/2}$C6A6T6Te, G2093G (GGA>GGT), G2094G (GGA>GGT) *tacI* and *araE*	This work
**6**	pAP111	ori p15A, cm^R, *araC-P$_{BAD}$* *xabCD tacI* and *araE*	This work
**7**	pAP112	ori p15A, cm^R, *araC-P$_{BAD}$* *xabCD*ΔBsaI *tacI* and *araE*	This work
**A2_D1**	pAP37_39	ori p15A, cm^R, *araC-P$_{BAD}$* *xldS*_C1A1T1$_{1/2}$_*ambS*_T1$_{1/2}$C/E2A2T2$_{1/2}$_*gxpS*_T4$_{1/2}$C/E5A5T5Te *tacI* and *araE*	This work
**A2_D2**	pAP37_40	ori p15A, cm^R, *araC-P$_{BAD}$* *xldS*_C1A1T1$_{1/2}$_*szeS*_T1$_{1/2}$C/E2A2T2$_{1/2}$_*gxpS*_T4$_{1/2}$C/E5A5T5Te *tacI* and *araE*	This work
**A2_D3**	pAP37_41	ori p15A, cm^R, *araC-P$_{BAD}$* *xldS*_C1A1T1$_{1/2}$_*szeS*_T4$_{1/2}$C/E5A5T5$_{1/2}$_*gxpS*_T4$_{1/2}$C/E5A5T5Te *tacI* and *araE*	This work
**A2_D4**	pAP37_42	ori p15A, cm^R, *araC-P$_{BAD}$* *xldS*_C1A1T1$_{1/2}$_*xtpS*_T2$_{1/2}$C3A3T3$_{1/2}$_*gxpS*_T4$_{1/2}$C/E5A5T5Te *tacI* and *araE*	This work
**A2_D5**	pAP37_43	ori p15A, cm^R, *araC-P$_{BAD}$* *xldS*_C1A1T1$_{1/2}$_*xabA*_T3$_{1/2}$C4A4T4$_{1/2}$	This work

NRPS-	Plasmids	Genotype	Reference
		_*gxpS*_T4$_{1/2}$C/E5A5T5Te *tacI* and *araE*	
**A2_D6**	pAP37_44	ori p15A, *cm*^{R}, *araC-P*$_{BAD}$ *xldS*_C1A1T1$_{1/2}$_*xabA*_T1$_{1/2}$C2A2T2$_{1/2}$_*gxpS*_T4$_{1/2}$C/E5A5T5Te *tacI* and *araE*	This work
**A1_D1**	pAP38_39	ori p15A, *cm*^{R}, *araC-P*$_{BAD}$ *xldS*_C1A1T1$_{1/2}$_*ambS*_T1$_{1/2}$C/E2A2T2$_{1/2}$_*szeS*_T5$_{1/2}$C6A6T6Te *tacI* and *araE*	This work
**A1_D2**	pAP38_40	ori p15A, *cm*^{R}, *araC-P*$_{BAD}$ *xldS*_C1A1T1$_{1/2}$_*szeS*_T1$_{1/2}$C/E2A2T2$_{1/2}$_*szeS*_T5$_{1/2}$C6A6T6Te *tacI* and *araE*	This work
**A1_D3**	pAP38_41	ori p15A, *cm*^{R}, *araC-P*$_{BAD}$ *xldS*_C1A1T1$_{1/2}$_*szeS*_T4$_{1/2}$C/E5A5T5$_{1/2}$_*szeS*_T5$_{1/2}$C6A6T6Te *tacI* and *araE*	This work
**A1_D4**	pAP38_42	ori p15A, *cm*^{R}, *araC-P*$_{BAD}$ *xldS*_C1A1T1$_{1/2}$_*xtpS*_T2$_{1/2}$C3A3T3$_{1/2}$_*szeS*_T5$_{1/2}$C6A6T6Te *tacI* and *araE*	This work
**A1_D5**	pAP38_43	ori p15A, *cm*^{R}, *araC-P*$_{BAD}$ *xldS*_C1A1T1$_{1/2}$_*xabA*_T3$_{1/2}$C4A4T4$_{1/2}$_*szeS*_T5$_{1/2}$C6A6T6Te *tacI* and *araE*	This work
**A1_D6**	pAP38_44	ori p15A, *cm*^{R}, *araC-P*$_{BAD}$ *xldS*_C1A1T1$_{1/2}$_*xabA*_T1$_{1/2}$C2A2T2$_{1/2}$_*szeS*_T5$_{1/2}$C6A6T6Te *tacI* and *araE*	This work
**A2_D7_D16**	pAP37_45_51	ori p15A, *cm*^{R}, *araC-P*$_{BAD}$ *xldS*_C1A1T1$_{1/2}$_*ambS*_T1$_{1/2}$C/E2A2T2$_{1/2}$_*ambS*_T1$_{1/2}$C/E2A2T2$_{1/2}$_*gxpS*_T4$_{1/2}$C/E5A5T5Te *tacI* and *araE*	This work
**A2_D7_D17**	pAP37_45_52	ori p15A, *cm*^{R}, *araC-P*$_{BAD}$ *xldS*_C1A1T1$_{1/2}$_*ambS*_T1$_{1/2}$C/E2A2T2$_{1/2}$_*szeS*_T1$_{1/2}$C/E2A2T2$_{1/2}$_*gxpS*_T4$_{1/2}$C/E5A5T5Te *tacI* and *araE*	This work
**A2_D7_D18**	pAP37_45_53	ori p15A, *cm*^{R}, *araC-P*$_{BAD}$ *xldS*_C1A1T1$_{1/2}$_*ambS*_T1$_{1/2}$C/E2A2T2$_{1/2}$_*szeS*_T4$_{1/2}$C/E5A5T5$_{1/2}$_*gxpS*_T4$_{1/2}$C/E5A5T5Te *tacI* and *araE*	This work
**A2_D7_D19**	pAP37_45_54	ori p15A, *cm*^{R}, *araC-P*$_{BAD}$ *xldS*_C1A1T1$_{1/2}$_*ambS*_T1$_{1/2}$C/E2A2T2$_{1/2}$_*xtpS*_T2$_{1/2}$C3A3T3$_{1/2}$_*gxpS*_T4$_{1/2}$C/E5A5T5Te *tacI* and *araE*	This work
**A2_D7_D20**	pAP37_45_55	ori p15A, *cm*^{R}, *araC-P*$_{BAD}$ *xldS*_C1A1T1$_{1/2}$_*ambS*_T1$_{1/2}$C/E2A2T2$_{1/2}$_*xabA*_T3$_{1/2}$C4A4T4$_{1/2}$_*gxpS*_T4$_{1/2}$C/E5A5T5Te *tacI* and *araE*	This work

NRPS-	Plasmids	Genotype	Reference
**A2_D7_D21**	pAP37_45_56	ori p15A, *cm*^{R}, *araC-P*$_{BAD}$ *xldS*_C1A1T1$_{1/2}$_*ambS*_T1$_{1/2}$C/E2A2T2$_{1/2}$_*xabA*_T1$_{1/2}$C2A2T2$_{1/2}$_*gxpS*_T4$_{1/2}$C/E5A5T5Te *tacI* and *araE*	This work
**A2_D8_D16**	pAP37_46_51	ori p15A, *cm*^{R}, *araC-P*$_{BAD}$ *xldS*_C1A1T1$_{1/2}$_*szeS*_T1$_{1/2}$C/E2A2T2$_{1/2}$_*ambS*_T1$_{1/2}$C/E2A2T2$_{1/2}$_*gxpS*_T4$_{1/2}$C/E5A5T5Te *tacI* and *araE*	This work
**A2_D8_D17**	pAP37_46_52	ori p15A, *cm*^{R}, *araC-P*$_{BAD}$ *xldS*_C1A1T1$_{1/2}$_*szeS*_T1$_{1/2}$C/E2A2T2$_{1/2}$_*szeS*_T1$_{1/2}$C/E2A2T2$_{1/2}$_*gxpS*_T4$_{1/2}$C/E5A5T5Te *tacI* and *araE*	This work
**A2_D8_D18**	pAP37_46_53	ori p15A, *cm*^{R}, *araC-P*$_{BAD}$ *xldS*_C1A1T1$_{1/2}$_*szeS*_T1$_{1/2}$C/E2A2T2$_{1/2}$_*szeS*_T4$_{1/2}$C/E5A5T5$_{1/2}$_*gxpS*_T4$_{1/2}$C/E5A5T5Te *tacI* and *araE*	This work
**A2_D8_D19**	pAP37_46_54	ori p15A, *cm*^{R}, *araC-P*$_{BAD}$ *xldS*_C1A1T1$_{1/2}$_*szeS*_T1$_{1/2}$C/E2A2T2$_{1/2}$_*xtpS*_T2$_{1/2}$C3A3T3$_{1/2}$_*gxpS*_T4$_{1/2}$C/E5A5T5Te *tacI* and *araE*	This work
**A2_D8_D20**	pAP37_46_55	ori p15A, *cm*^{R}, *araC-P*$_{BAD}$ *xldS*_C1A1T1$_{1/2}$_*szeS*_T1$_{1/2}$C/E2A2T2$_{1/2}$_*xabA*_T3$_{1/2}$C4A4T4$_{1/2}$_*gxpS*_T4$_{1/2}$C/E5A5T5Te *tacI* and *araE*	This work
**A2_D8_D21**	pAP37_46_56	ori p15A, *cm*^{R}, *araC-P*$_{BAD}$ *xldS*_C1A1T1$_{1/2}$_*szeS*_T1$_{1/2}$C/E2A2T2$_{1/2}$_*xabA*_T1$_{1/2}$C2A2T2$_{1/2}$_*gxpS*_T4$_{1/2}$C/E5A5T5Te *tacI* and *araE*	This work
**A2_D9_D16**	pAP37_47_51	ori p15A, *cm*^{R}, *araC-P*$_{BAD}$ *xldS*_C1A1T1$_{1/2}$_*szeS*_T4$_{1/2}$C/E5A5T5$_{1/2}$_*ambS*_T1$_{1/2}$C/E2A2T2$_{1/2}$_*gxpS*_T4$_{1/2}$C/E5A5T5Te *tacI* and *araE*	This work
**A2_D9_D17**	pAP37_47_52	ori p15A, *cm*^{R}, *araC-P*$_{BAD}$ *xldS*_C1A1T1$_{1/2}$_*szeS*_T4$_{1/2}$C/E5A5T5$_{1/2}$_*szeS*_T1$_{1/2}$C/E2A2T2$_{1/2}$_*gxpS*_T4$_{1/2}$C/E5A5T5Te *tacI* and *araE*	This work
**A2_D9_D18**	pAP37_47_53	ori p15A, *cm*^{R}, *araC-P*$_{BAD}$ *xldS*_C1A1T1$_{1/2}$_*szeS*_T4$_{1/2}$C/E5A5T5$_{1/2}$_*szeS*_T4$_{1/2}$C/E5A5T5$_{1/2}$_*gxpS*_T4$_{1/2}$C/E5A5T5Te *tacI* and *araE*	This work
**A2_D9_D19**	pAP37_47_54	ori p15A, *cm*^{R}, *araC-P*$_{BAD}$ *xldS*_C1A1T1$_{1/2}$_*szeS*_T4$_{1/2}$C/E5A5T5$_{1/2}$_*xtpS*_T2$_{1/2}$C3A3T3$_{1/2}$_*gxpS*_T4$_{1/2}$C/E5A5T5Te *tacI* and *araE*	This work
**A2_D9_D20**	pAP37_47_55	ori p15A, *cm*^{R}, *araC-P*$_{BAD}$ *xldS*_C1A1T1$_{1/2}$_*szeS*_T4$_{1/2}$C/E5A5T5$_{1/2}$_*xabA*_T3$_{1/2}$C4A4T4$_{1/2}$_*gxpS*_T4$_{1/2}$C/E5A5T5Te *tacI* and *araE*	This work

NRPS-	Plasmids	Genotype	Reference
**A2_D9_D21**	pAP37_47_56	ori p15A, *cm*^{R}, *araC-P*$_{BAD}$ *xldS*_C1A1T1$_{1/2}$_*szeS*_T4$_{1/2}$C/E5A5T5$_{1/2}$_*xabA*_T1$_{1/2}$C2A2T2$_{1/2}$_*gxpS*_T4$_{1/2}$C/E5A5T5Te *tacI* and *araE*	This work
**A2_D10_D16**	pAP37_48_51	ori p15A, *cm*^{R}, *araC-P*$_{BAD}$ *xldS*_C1A1T1$_{1/2}$_*xtpS*_T2$_{1/2}$C3A3T3$_{1/2}$_*ambS*_T1$_{1/2}$C/E2A2T2$_{1/2}$_*gxpS*_T4$_{1/2}$C/E5A5T5Te *tacI* and *araE*	This work
**A2_D10_D17**	pAP37_48_52	ori p15A, *cm*^{R}, *araC-P*$_{BAD}$ *xldS*_C1A1T1$_{1/2}$_*xtpS*_T2$_{1/2}$C3A3T3$_{1/2}$_*szeS*_T1$_{1/2}$C/E2A2T2$_{1/2}$_*gxpS*_T4$_{1/2}$C/E5A5T5Te *tacI* and *araE*	This work
**A2_D10_D18**	pAP37_48_53	ori p15A, *cm*^{R}, *araC-P*$_{BAD}$ *xldS*_C1A1T1$_{1/2}$_*xtpS*_T2$_{1/2}$C3A3T3$_{1/2}$_*szeS*_T4$_{1/2}$C/E5A5T5$_{1/2}$_*gxpS*_T4$_{1/2}$C/E5A5T5Te *tacI* and *araE*	This work
**A2_D10_D19**	pAP37_48_54	ori p15A, *cm*^{R}, *araC-P*$_{BAD}$ *xldS*_C1A1T1$_{1/2}$_*xtpS*_T2$_{1/2}$C3A3T3$_{1/2}$_*xtpS*_T2$_{1/2}$C3A3T3$_{1/2}$_*gxpS*_T4$_{1/2}$C/E5A5T5Te *tacI* and *araE*	This work
**A2_D10_D20**	pAP37_48_55	ori p15A, *cm*^{R}, *araC-P*$_{BAD}$ *xldS*_C1A1T1$_{1/2}$_*xtpS*_T2$_{1/2}$C3A3T3$_{1/2}$_*xabA*_T3$_{1/2}$C4A4T4$_{1/2}$_*gxpS*_T4$_{1/2}$C/E5A5T5Te *tacI* and *araE*	This work
**A2_D10_D21**	pAP37_48_56	ori p15A, *cm*^{R}, *araC-P*$_{BAD}$ *xldS*_C1A1T1$_{1/2}$_*xtpS*_T2$_{1/2}$C3A3T3$_{1/2}$_*xabA*_T1$_{1/2}$C2A2T2$_{1/2}$_*gxpS*_T4$_{1/2}$C/E5A5T5Te *tacI* and *araE*	This work
**A2_D11_D16**	pAP37_49_51	ori p15A, *cm*^{R}, *araC-P*$_{BAD}$ *xldS*_C1A1T1$_{1/2}$_*xabA*_T3$_{1/2}$C4A4T4$_{1/2}$_*ambS*_T1$_{1/2}$C/E2A2T2$_{1/2}$_*gxpS*_T4$_{1/2}$C/E5A5T5Te *tacI* and *araE*	This work
**A2_D11_D17**	pAP37_49_52	ori p15A, *cm*^{R}, *araC-P*$_{BAD}$ *xldS*_C1A1T1$_{1/2}$_*xabA*_T3$_{1/2}$C4A4T4$_{1/2}$_*szeS*_T1$_{1/2}$C/E2A2T2$_{1/2}$_*gxpS*_T4$_{1/2}$C/E5A5T5Te *tacI* and *araE*	This work
**A2_D11_D18**	pAP37_49_53	ori p15A, *cm*^{R}, *araC-P*$_{BAD}$ *xldS*_C1A1T1$_{1/2}$_*xabA*_T3$_{1/2}$C4A4T4$_{1/2}$_*szeS*_T4$_{1/2}$C/E5A5T5$_{1/2}$_*gxpS*_T4$_{1/2}$C/E5A5T5Te *tacI* and *araE*	This work
**A2_D11_D19**	pAP37_49_54	ori p15A, *cm*^{R}, *araC-P*$_{BAD}$ *xldS*_C1A1T1$_{1/2}$_*xabA*_T3$_{1/2}$C4A4T4$_{1/2}$_*xtpS*_T2$_{1/2}$C3A3T3$_{1/2}$_*gxpS*_T4$_{1/2}$C/E5A5T5Te *tacI* and *araE*	This work
**A2_D11_D20**	pAP37_49_55	ori p15A, *cm*^{R}, *araC-P*$_{BAD}$ *xldS*_C1A1T1$_{1/2}$_*xabA*_T3$_{1/2}$C4A4T4$_{1/2}$_*xabA*_T3$_{1/2}$C4A4T4$_{1/2}$_*gxpS*_T4$_{1/2}$C/E5A5T5Te *tacI* and *araE*	This work

NRPS-	Plasmids	Genotype	Reference
**A2_D11_D21**	pAP37_49_56	ori p15A, cm^R, *araC-P_{BAD}* *xldS*_C1A1T1$_{1/2}$_*xabA*_T3$_{1/2}$C4A4T4$_{1/2}$ _*xabA*_T1$_{1/2}$C2A2T2$_{1/2}$_*gxpS*_T4$_{1/2}$C/ E5A5T5Te *tacI* and *araE*	This work
**A2_D12_D16**	pAP37_50_51	ori p15A, cm^R, *araC-P_{BAD}* *xldS*_C1A1T1$_{1/2}$_*xabA*_T1$_{1/2}$C2A2T2$_{1/2}$ _*ambS*_T1$_{1/2}$C/E2A2T2$_{1/2}$_*gxpS*_T4$_{1/2}$ C/E5A5T5Te *tacI* and *araE*	This work
**A2_D12_D17**	pAP37_50_52	ori p15A, cm^R, *araC-P_{BAD}* *xldS*_C1A1T1$_{1/2}$_*xabA*_T1$_{1/2}$C2A2T2$_{1/2}$ _*szeS*_T1$_{1/2}$C/E2A2T2$_{1/2}$_*gxpS*_T4$_{1/2}$C /E5A5T5Te *tacI* and *araE*	This work
**A2_D12_D18**	pAP37_50_53	ori p15A, cm^R, *araC-P_{BAD}* *xldS*_C1A1T1$_{1/2}$_*xabA*_T1$_{1/2}$C2A2T2$_{1/2}$ _*szeS*_T4$_{1/2}$C/E5A5T5$_{1/2}$_*gxpS*_T4$_{1/2}$C /E5A5T5Te *tacI* and *araE*	This work
**A2_D12_D19**	pAP37_50_54	ori p15A, cm^R, *araC-P_{BAD}* *xldS*_C1A1T1$_{1/2}$_*xabA*_T1$_{1/2}$C2A2T2$_{1/2}$ _*xtpS*_T2$_{1/2}$C3A3T3$_{1/2}$_*gxpS*_T4$_{1/2}$C/E 5A5T5Te *tacI* and *araE*	This work
**A2_D12_D20**	pAP37_50_55	ori p15A, cm^R, *araC-P_{BAD}* *xldS*_C1A1T1$_{1/2}$_*xabA*_T1$_{1/2}$C2A2T2$_{1/2}$ _*xabA*_T3$_{1/2}$C4A4T4$_{1/2}$_*gxpS*_T4$_{1/2}$C/ E5A5T5Te *tacI* and *araE*	This work
**A2_D12_D21**	pAP37_50_56	ori p15A, cm^R, *araC-P_{BAD}* *xldS*_C1A1T1$_{1/2}$_*xabA*_T1$_{1/2}$C2A2T2$_{1/2}$ _*xabA*_T1$_{1/2}$C2A2T2$_{1/2}$_*gxpS*_T4$_{1/2}$C/ E5A5T5Te *tacI* and *araE*	This work
**A2_D13_D16**	pAP37_95_51	ori p15A, cm^R, *araC-P_{BAD}* *xldS*_C1A1T1$_{1/2}$_*xabB*_T7$_{1/2}$C8A8T8$_{1/2}$ _*ambS*_T1$_{1/2}$C/E2A2T2$_{1/2}$_*gxpS*_T4$_{1/2}$ C/E5A5T5Te *tacI* and *araE*	This work
**A2_D13_D17**	pAP37_95_52	ori p15A, cm^R, *araC-P_{BAD}* *xldS*_C1A1T1$_{1/2}$_*xabB*_T7$_{1/2}$C8A8T8$_{1/2}$ _*szeS*_T1$_{1/2}$C/E2A2T2$_{1/2}$_*gxpS*_T4$_{1/2}$C /E5A5T5Te *tacI* and *araE*	This work
**A2_D13_D18**	pAP37_95_53	ori p15A, cm^R, *araC-P_{BAD}* *xldS*_C1A1T1$_{1/2}$_*xabB*_T7$_{1/2}$C8A8T8$_{1/2}$ _*szeS*_T4$_{1/2}$C/E5A5T5$_{1/2}$_*gxpS*_T4$_{1/2}$C /E5A5T5Te *tacI* and *araE*	This work
**A2_D13_D19**	pAP37_95_54	ori p15A, cm^R, *araC-P_{BAD}* *xldS*_C1A1T1$_{1/2}$_*xabB*_T7$_{1/2}$C8A8T8$_{1/2}$ _*xtpS*_T2$_{1/2}$C3A3T3$_{1/2}$_*gxpS*_T4$_{1/2}$C/E 5A5T5Te *tacI* and *araE*	This work
**A2_D13_D20**	pAP37_95_55	ori p15A, cm^R, *araC-P_{BAD}* *xldS*_C1A1T1$_{1/2}$_*xabB*_T7$_{1/2}$C8A8T8$_{1/2}$ _*xabA*_T3$_{1/2}$C4A4T4$_{1/2}$_*gxpS*_T4$_{1/2}$C/ E5A5T5Te *tacI* and *araE*	This work

NRPS-	Plasmids	Genotype	Reference
**A2_D13_D21**	pAP37_95_56	ori p15A, *cm*^{R}, *araC-P*$_{BAD}$ *xldS*_C1A1T1$_{1/2}$_*xabB*_T7$_{1/2}$C8A8T8$_{1/2}$_*xabA*_T1$_{1/2}$C2A2T2$_{1/2}$_*gxpS*_T4$_{1/2}$C/E5A5T5Te *tacI* and *araE*	This work
**A2_D14_D16**	pAP37_96_51	ori p15A, *cm*^{R}, *araC-P*$_{BAD}$ *xldS*_C1A1T1$_{1/2}$_*xabA*_T4$_{1/2}$C5A5T5$_{1/2}$_*ambS*_T1$_{1/2}$C/E2A2T2$_{1/2}$_*gxpS*_T4$_{1/2}$C/E5A5T5Te *tacI* and *araE*	This work
**A2_D14_D17**	pAP37_96_52	ori p15A, *cm*^{R}, *araC-P*$_{BAD}$ *xldS*_C1A1T1$_{1/2}$_*xabA*_T4$_{1/2}$C5A5T5$_{1/2}$_*szeS*_T1$_{1/2}$C/E2A2T2$_{1/2}$_*gxpS*_T4$_{1/2}$C/E5A5T5Te *tacI* and *araE*	This work
**A2_D14_D18**	pAP37_96_53	ori p15A, *cm*^{R}, *araC-P*$_{BAD}$ *xldS*_C1A1T1$_{1/2}$_*xabA*_T4$_{1/2}$C5A5T5$_{1/2}$_*szeS*_T4$_{1/2}$C/E5A5T5$_{1/2}$_*gxpS*_T4$_{1/2}$C/E5A5T5Te *tacI* and *araE*	This work
**A2_D14_D19**	pAP37_96_54	ori p15A, *cm*^{R}, *araC-P*$_{BAD}$ *xldS*_C1A1T1$_{1/2}$_*xabA*_T4$_{1/2}$C5A5T5$_{1/2}$_*xtpS*_T2$_{1/2}$C3A3T3$_{1/2}$_*gxpS*_T4$_{1/2}$C/E5A5T5Te *tacI* and *araE*	This work
**A2_D14_D20**	pAP37_96_55	ori p15A, *cm*^{R}, *araC-P*$_{BAD}$ *xldS*_C1A1T1$_{1/2}$_*xabA*_T4$_{1/2}$C5A5T5$_{1/2}$_*xabA*_T3$_{1/2}$C4A4T4$_{1/2}$_*gxpS*_T4$_{1/2}$C/E5A5T5Te *tacI* and *araE*	This work
**A2_D14_D21**	pAP37_96_56	ori p15A, *cm*^{R}, *araC-P*$_{BAD}$ *xldS*_C1A1T1$_{1/2}$_*xabA*_T4$_{1/2}$C5A5T5$_{1/2}$_*xabA*_T1$_{1/2}$C2A2T2$_{1/2}$_*gxpS*_T4$_{1/2}$C/E5A5T5Te *tacI* and *araE*	This work
**A2_D7_D22**	pAP37_45_98	ori p15A, *cm*^{R}, *araC-P*$_{BAD}$ *xldS*_C1A1T1$_{1/2}$_*ambS*_T1$_{1/2}$C/E2A2T2$_{1/2}$_*xabB*_T7$_{1/2}$C8A8T8$_{1/2}$_*gxpS*_T4$_{1/2}$C/E5A5T5Te *tacI* and *araE*	This work
**A2_D8_D22**	pAP37_46_98	ori p15A, *cm*^{R}, *araC-P*$_{BAD}$ *xldS*_C1A1T1$_{1/2}$_*szeS*_T1$_{1/2}$C/E2A2T2$_{1/2}$_*xabB*_T7$_{1/2}$C8A8T8$_{1/2}$_*gxpS*_T4$_{1/2}$C/E5A5T5Te *tacI* and *araE*	This work
**A2_D9_D22**	pAP37_47_98	ori p15A, *cm*^{R}, *araC-P*$_{BAD}$ *xldS*_C1A1T1$_{1/2}$_*szeS*_T4$_{1/2}$C/E5A5T5$_{1/2}$_*xabB*_T7$_{1/2}$C8A8T8$_{1/2}$_*gxpS*_T4$_{1/2}$C/E5A5T5Te *tacI* and *araE*	This work
**A2_D10_D22**	pAP37_48_98	ori p15A, *cm*^{R}, *araC-P*$_{BAD}$ *xldS*_C1A1T1$_{1/2}$_*xtpS*_T2$_{1/2}$C3A3T3$_{1/2}$_*xabB*_T7$_{1/2}$C8A8T8$_{1/2}$_*gxpS*_T4$_{1/2}$C/E5A5T5Te *tacI* and *araE*	This work
**A2_D11_D22**	pAP37_49_98	ori p15A, *cm*^{R}, *araC-P*$_{BAD}$ *xldS*_C1A1T1$_{1/2}$_*xabA*_T3$_{1/2}$C4A4T4$_{1/2}$_*xabB*_T7$_{1/2}$C8A8T8$_{1/2}$_*gxpS*_T4$_{1/2}$C/E5A5T5Te *tacI* and *araE*	This work

NRPS-	Plasmids	Genotype	Reference
**A2_D12_D22**	pAP37_50_98	ori p15A, *cm*^{R}, *araC-P*$_{BAD}$ *xldS*_C1A1T1$_{1/2}$_*xabA*_T1$_{1/2}$C2A2T2$_{1/2}$_*xabB*_T7$_{1/2}$C8A8T8$_{1/2}$_*gxpS*_T4$_{1/2}$C/E5A5T5Te *tacI* and *araE*	This work
**A2_D7_D23**	pAP37_45_99	ori p15A, *cm*^{R}, *araC-P*$_{BAD}$ *xldS*_C1A1T1$_{1/2}$_*ambS*_T1$_{1/2}$C/E2A2T2$_{1/2}$_*xabA*_T4$_{1/2}$C5A5T5$_{1/2}$_*gxpS*_T4$_{1/2}$C/E5A5T5Te *tacI* and *araE*	This work
**A2_D8_D23**	pAP37_46_99	ori p15A, *cm*^{R}, *araC-P*$_{BAD}$ *xldS*_C1A1T1$_{1/2}$_*szeS*_T1$_{1/2}$C/E2A2T2$_{1/2}$_*xabA*_T4$_{1/2}$C5A5T5$_{1/2}$_*gxpS*_T4$_{1/2}$C/E5A5T5Te *tacI* and *araE*	This work
**A2_D9_D23**	pAP37_47_99	ori p15A, *cm*^{R}, *araC-P*$_{BAD}$ *xldS*_C1A1T1$_{1/2}$_*szeS*_T4$_{1/2}$C/E5A5T5$_{1/2}$_*xabA*_T4$_{1/2}$C5A5T5$_{1/2}$_*gxpS*_T4$_{1/2}$C/E5A5T5Te *tacI* and *araE*	This work
**A2_D10_D23**	pAP37_48_99	ori p15A, *cm*^{R}, *araC-P*$_{BAD}$ *xldS*_C1A1T1$_{1/2}$_*xtpS*_T2$_{1/2}$C3A3T3$_{1/2}$_*xabA*_T4$_{1/2}$C5A5T5$_{1/2}$_*gxpS*_T4$_{1/2}$C/E5A5T5Te *tacI* and *araE*	This work
**A2_D11_D23**	pAP37_49_99	ori p15A, *cm*^{R}, *araC-P*$_{BAD}$ *xldS*_C1A1T1$_{1/2}$_*xabA*_T3$_{1/2}$C4A4T4$_{1/2}$_*xabA*_T4$_{1/2}$C5A5T5$_{1/2}$_*gxpS*_T4$_{1/2}$C/E5A5T5Te *tacI* and *araE*	This work
**A2_D12_D23**	pAP37_50_99	ori p15A, *cm*^{R}, *araC-P*$_{BAD}$ *xldS*_C1A1T1$_{1/2}$_*xabA*_T1$_{1/2}$C2A2T2$_{1/2}$_*xabA*_T4$_{1/2}$C5A5T5$_{1/2}$_*gxpS*_T4$_{1/2}$C/E5A5T5Te *tacI* and *araE*	This work
**A2_D13_D22**	pAP37_95_98	ori p15A, *cm*^{R}, *araC-P*$_{BAD}$ *xldS*_C1A1T1$_{1/2}$_*xabB*_T7$_{1/2}$C8A8T8$_{1/2}$_*xabB_T7*$_{1/2}$*C8A8T8*$_{1/2}$_*gxpS*_T4$_{1/2}$C/E5A5T5Te *tacI* and *araE*	This work
**A2_D13_D23**	pAP37_95_99	ori p15A, *cm*^{R}, *araC-P*$_{BAD}$ *xldS*_C1A1T1$_{1/2}$_*xabB*_T7$_{1/2}$C8A8T8$_{1/2}$_*xabA*_T4$_{1/2}$C5A5T5$_{1/2}$_*gxpS*_T4$_{1/2}$C/E5A5T5Te *tacI* and *araE*	This work
**A2_D14_D22**	pAP37_96_98	ori p15A, *cm*^{R}, *araC-P*$_{BAD}$ *xldS*_C1A1T1$_{1/2}$_*xabA_T4*$_{1/2}$*C5A5T5*$_{1/2}$_*xabB_T7*$_{1/2}$*C8A8T8*$_{1/2}$_*gxpS*_T4$_{1/2}$C/E5A5T5Te *tacI* and *araE*	This work
**A2_D14_D23**	pAP37_96_99	ori p15A, *cm*^{R}, *araC-P*$_{BAD}$ *xldS*_C1A1T1$_{1/2}$_*xabA_T4*$_{1/2}$*C5A5T5*$_{1/2}$_*xabA*_T4$_{1/2}$C5A5T5$_{1/2}$_*gxpS*_T4$_{1/2}$C/E5A5T5Te *tacI* and *araE*	This work
**A2_D15_D16**	pAP37_89_51	ori p15A, *cm*^{R}, *araC-P*$_{BAD}$ *xldS*_C1A1T1$_{1/2}$_*fitAB*_T3$_{1/2}$EC5A5T5$_{1/2}$_*ambS*_T1$_{1/2}$C/E2A2T2$_{1/2}$_*gxpS*_T4$_{1/2}$C/E5A5T5Te *tacI* and *araE*	This work

NRPS-	Plasmids	Genotype	Reference
**A2_D15_D17**	pAP37_89_52	ori p15A, *cm*^{R}, *araC-P*$_{BAD}$ *xldS*_C1A1T1$_{1/2}$_*fitAB*_T3$_{1/2}$EC5A5T5$_{1/2}$_*szeS*_T1$_{1/2}$C/E2A2T2$_{1/2}$_*gxpS*_T4$_{1/2}$C/E5A5T5Te *tacI* and *araE*	This work
**A2_D15_D18**	pAP37_89_53	ori p15A, *cm*^{R}, *araC-P*$_{BAD}$ *xldS*_C1A1T1$_{1/2}$_*fitAB*_T3$_{1/2}$EC5A5T5$_{1/2}$_*szeS*_T4$_{1/2}$C/E5A5T5$_{1/2}$_*gxpS*_T4$_{1/2}$C/E5A5T5Te *tacI* and *araE*	This work
**A2_D15_D19**	pAP37_89_54	ori p15A, *cm*^{R}, *araC-P*$_{BAD}$ *xldS*_C1A1T1$_{1/2}$_*fitAB*_T3$_{1/2}$EC5A5T5$_{1/2}$_*xtpS*_T2$_{1/2}$C3A3T3$_{1/2}$_*gxpS*_T4$_{1/2}$C/E5A5T5Te *tacI* and *araE*	This work
**A2_D15_D20**	pAP37_89_55	ori p15A, *cm*^{R}, *araC-P*$_{BAD}$ *xldS*_C1A1T1$_{1/2}$_*fitAB*_T3$_{1/2}$EC5A5T5$_{1/2}$_*xabA*_T3$_{1/2}$C4A4T4$_{1/2}$_*gxpS*_T4$_{1/2}$C/E5A5T5Te *tacI* and *araE*	This work
**A2_D15_D21**	pAP37_89_56	ori p15A, *cm*^{R}, *araC-P*$_{BAD}$ *xldS*_C1A1T1$_{1/2}$_*fitAB*_T3$_{1/2}$EC5A5T5$_{1/2}$_*xabA*_T1$_{1/2}$C2A2T2$_{1/2}$_*gxpS*_T4$_{1/2}$C/E5A5T5Te *tacI* and *araE*	This work
**A2_D7_D24**	pAP37_45_92	ori p15A, *cm*^{R}, *araC-P*$_{BAD}$ *xldS*_C1A1T1$_{1/2}$_*ambS*_T1$_{1/2}$C/E2A2T2$_{1/2}$_*fitAB*_T3$_{1/2}$EC5A5T5$_{1/2}$_*gxpS*_T4$_{1/2}$C/E5A5T5Te *tacI* and *araE*	This work
**A2_D8_D24**	pAP37_46_92	ori p15A, *cm*^{R}, *araC-P*$_{BAD}$ *xldS*_C1A1T1$_{1/2}$_*szeS*_T1$_{1/2}$C/E2A2T2$_{1/2}$_*fitAB*_T3$_{1/2}$EC5A5T5$_{1/2}$_*gxpS*_T4$_{1/2}$C/E5A5T5Te *tacI* and *araE*	This work
**A2_D9_D24**	pAP37_47_92	ori p15A, *cm*^{R}, *araC-P*$_{BAD}$ *xldS*_C1A1T1$_{1/2}$_*szeS*_T4$_{1/2}$C/E5A5T5$_{1/2}$_*fitAB*_T3$_{1/2}$EC5A5T5$_{1/2}$_*gxpS*_T4$_{1/2}$C/E5A5T5Te *tacI* and *araE*	This work
**A2_D10_D24**	pAP37_48_92	ori p15A, *cm*^{R}, *araC-P*$_{BAD}$ *xldS*_C1A1T1$_{1/2}$_*xtpS*_T2$_{1/2}$C3A3T3$_{1/2}$_*fitAB*_T3$_{1/2}$EC5A5T5$_{1/2}$_*gxpS*_T4$_{1/2}$C/E5A5T5Te *tacI* and *araE*	This work
**A2_D11_D24**	pAP37_49_92	ori p15A, *cm*^{R}, *araC-P*$_{BAD}$ *xldS*_C1A1T1$_{1/2}$_*xabA*_T3$_{1/2}$C4A4T4$_{1/2}$_*fitAB*_T3$_{1/2}$EC5A5T5$_{1/2}$_*gxpS*_T4$_{1/2}$C/E5A5T5Te *tacI* and *araE*	This work
**A2_D12_D24**	pAP37_50_92	ori p15A, *cm*^{R}, *araC-P*$_{BAD}$ *xldS*_C1A1T1$_{1/2}$_*xabA*_T1$_{1/2}$C2A2T2$_{1/2}$_*fitAB*_T3$_{1/2}$EC5A5T5$_{1/2}$_*gxpS*_T4$_{1/2}$C/E5A5T5Te *tacI* and *araE*	This work
**A2_D15_D24**	pAP37_89_92	ori p15A, *cm*^{R}, *araC-P*$_{BAD}$ *xldS*_C1A1T1$_{1/2}$_*fitAB*_T3$_{1/2}$EC5A5T5$_{1/2}$_*fitAB*_T3$_{1/2}$EC5A5T5$_{1/2}$_*gxpS*_T4$_{1/2}$C/E5A5T5Te *tacI* and *araE*	This work

NRPS-	Plasmids	Genotype	Reference
**A2_D15_D22**	pAP37_89_98	ori p15A, *cm*^{R}, *araC-P*$_{BAD}$ *xldS*_C1A1T1$_{1/2}$_*fitAB*_T3$_{1/2}$EC5A5T5$_{1/2}$_*xabB*_T7$_{1/2}$C8A8T8$_{1/2}$_*gxpS*_T4$_{1/2}$C/E5A5T5Te *tacI* and *araE*	This work
**A2_D15_D23**	pAP37_89_99	ori p15A, *cm*^{R}, *araC-P*$_{BAD}$ *xldS*_C1A1T1$_{1/2}$_*fitAB*_T3$_{1/2}$EC5A5T5$_{1/2}$_*xabA*_T4$_{1/2}$C5A5T5$_{1/2}$_*gxpS*_T4$_{1/2}$C/E5A5T5Te *tacI* and *araE*	This work
**A2_D13_D24**	pAP37_95_92	ori p15A, *cm*^{R}, *araC-P*$_{BAD}$ *xldS*_C1A1T1$_{1/2}$_*xabB*_T7$_{1/2}$C8A8T8$_{1/2}$_*fitAB*_T3$_{1/2}$EC5A5T5$_{1/2}$_*gxpS*_T4$_{1/2}$C/E5A5T5Te *tacI* and *araE*	This work
**A2_D14_D24**	pAP37_96_92	ori p15A, *cm*^{R}, *araC-P*$_{BAD}$ *xldS*_C1A1T1$_{1/2}$_*xabA*_T4$_{1/2}$C5A5T5$_{1/2}$_*fitAB*_T3$_{1/2}$EC5A5T5$_{1/2}$_*gxpS*_T4$_{1/2}$C/E5A5T5Te *tacI* and *araE*	This work
**A3_D25**	pAP106_107	ori p15A, *cm*^{R}, *araC-P*$_{BAD}$ *xldS*_C1A1T1$_{1/2}$_*xabA*_T1$_{1/2}$C2A2T2$_{1/2}$_*gxpS*_T4$_{1/2}$C/E5A5T5Te *tacI* and *araE*	This work
**A3_D26**	pAP106_108	ori p15A, *cm*^{R}, *araC-P*$_{BAD}$ *xabA*_C1A1T1$_{1/2}$_*xabA*_T1$_{1/2}$C2A2T2$_{1/2}$_*gxpS*_T4$_{1/2}$C/E5A5T5Te *tacI* and *araE*	This work
**A3_D27**	pAP106_109	ori p15A, *cm*^{R}, *araC-P*$_{BAD}$ *ambS*_C1A1T1$_{1/2}$_*xabA*_T1$_{1/2}$C2A2T2$_{1/2}$_*gxpS*_T4$_{1/2}$C/E5A5T5Te *tacI* and *araE*	This work
**A3_D28**	pAP106_110	ori p15A, *cm*^{R}, *araC-P*$_{BAD}$ XlSHV2_07515_C1A1T1$_{1/2}$_*xabA*_T1$_{1/2}$C2A2T2$_{1/2}$_*gxpS*_T4$_{1/2}$C/E5A5T5Te *tacI* and *araE*	This work
**A4_D29**	pAP119_113	ori p15A, *cm*^{R}, *araC-P*$_{BAD}$ *xabC*_C1A1T1$_{1/2}$_*xabC*_T1$_{1/2}$C/E2A2T2$_{1/2}$_*xabC*_T3$_{1/2}$C4A4T4TeTe *tacI* and *araE*	This work
**A4_D30**	pAP119_114	ori p15A, *cm*^{R}, *araC-P*$_{BAD}$ *xabC*_C1A1T1$_{1/2}$_*xabC*_T2$_{1/2}$C3A3T3$_{1/2}$_*xabC*_T3$_{1/2}$C4A4T4TeTe *tacI* and *araE*	This work
**A4_D31_D33**	pAP119_115_117	ori p15A, *cm*^{R}, *araC-P*$_{BAD}$ *xabC*_C1A1T1$_{1/2}$_*xabC*_T1$_{1/2}$C/E2A2T2$_{1/2}$_*xabC*_T11$_{1/2}$C/E2A2T2$_{1/2}$_*xabC*_T3$_{1/2}$C4A4T4TeTe *tacI* and *araE*	This work
**A4_D31_D34**	pAP119_115_118	ori p15A, *cm*^{R}, *araC-P*$_{BAD}$ *xabC*_C1A1T1$_{1/2}$_*xabC*_T1$_{1/2}$C/E2A2T2$_{1/2}$_*xabC*_T2$_{1/2}$C3A3T3$_{1/2}$_*xabC*_T3$_{1/2}$C4A4T4TeTe *tacI* and *araE*	This work

NRPS-	Plasmids	Genotype	Reference
**A4_D32_D33**	pAP119_116_117	ori p15A, *cm*^{R}, *araC-P*$_{BAD}$ *xabC*_C1A1T1$_{1/2}$_*xabC*_T2$_{1/2}$C/E3A3T3$_{1/2}$_*xabC*_T1$_{1/2}$C/E2A2T2$_{1/2}$_*xabC*_T3$_{1/2}$C4A4T4TeTe *tacI* and *araE*	This work
**A4_D32_D34**	pAP119_116_118	ori p15A, *cm*^{R}, *araC-P*$_{BAD}$ *xabC*_C1A1T1$_{1/2}$_*xabC*_T2$_{1/2}$C3A3T3$_{1/2}$_*xabC*_T2$_{1/2}$C3A3T3$_{1/2}$_*xabC*_T3$_{1/2}$C4A4T4TeTe *tacI* and *araE*	This work
**A4_D1**	pAP119_39	ori p15A, *cm*^{R}, *araC-P*$_{BAD}$ *xabC*_C1A1T1$_{1/2}$_ambS_T1$_{1/2}$C/E2A2T2$_{1/2}$_*xabC*_T3$_{1/2}$C4A4T4TeTe *tacI* and *araE*	This work
**A4_D2**	pAP119_40	ori p15A, *cm*^{R}, *araC-P*$_{BAD}$ *xabC*_C1A1T1$_{1/2}$_szeS_T1$_{1/2}$C/E2A2T2$_{1/2}$_*xabC*_T3$_{1/2}$C4A4T4TeTe *tacI* and *araE*	This work
**A4_D3**	pAP119_41	ori p15A, *cm*^{R}, *araC-P*$_{BAD}$ *xabC*_C1A1T1$_{1/2}$_szeS_T4$_{1/2}$C/E5A5T5$_{1/2}$_*xabC*_T3$_{1/2}$C4A4T4TeTe *tacI* and *araE*	This work
**A4_D4**	pAP119_42	ori p15A, *cm*^{R}, *araC-P*$_{BAD}$ *xabC*_C1A1T1$_{1/2}$_xtpS_T2$_{1/2}$C3A3T3$_{1/2}$_*xabC*_T3$_{1/2}$C4A4T4TeTe *tacI* and *araE*	This work
**A4_D5**	pAP119_43	ori p15A, *cm*^{R}, *araC-P*$_{BAD}$ *xabC*_C1A1T1$_{1/2}$_xabA_T3$_{1/2}$C4A4T4$_{1/2}$_gxpS_*xabC*_T3$_{1/2}$C4A4T4TeTe *tacI* and *araE*	This work
**A4_D6**	pAP119_44	ori p15A, *cm*^{R}, *araC-P*$_{BAD}$ *xabC*_C1A1T1$_{1/2}$_xabA_T1$_{1/2}$C2A2T2$_{1/2}$_gxpS_*xabC*_T3$_{1/2}$C4A4T4TeTe *tacI* and *araE*	This work
**A4_D7_D16**	pAP119_45_51	ori p15A, *cm*^{R}, *araC-P*$_{BAD}$ *xab*C_C1A1T1$_{1/2}$_*ambS*_T1$_{1/2}$C/E2A2T2$_{1/2}$_*ambS*_T1$_{1/2}$C/E2A2T2$_{1/2}$_*xabC*_T3$_{1/2}$C4A4T4TeTe *tacI* and *araE*	This work
**A4_D7_D17**	pAP119_45_52	ori p15A, *cm*^{R}, *araC-P*$_{BAD}$ *xabC*_C1A1T1$_{1/2}$_*ambS*_T1$_{1/2}$C/E2A2T2$_{1/2}$_*szeS*_T1$_{1/2}$C/E2A2T2$_{1/2}$_*xabC*_T3$_{1/2}$C4A4T4TeTe *tacI* and *araE*	This work
**A4_D7_D18**	pAP119_45_53	ori p15A, *cm*^{R}, *araC-P*$_{BAD}$ *xabC*_C1A1T1$_{1/2}$_*ambS*_T1$_{1/2}$C/E2A2T2$_{1/2}$_*szeS*_T4$_{1/2}$C/E5A5T5$_{1/2}$_*xabC*_T3$_{1/2}$C4A4T4TeTe *tacI* and *araE*	This work
**A4_D7_D19**	pAP119_45_54	ori p15A, *cm*^{R}, *araC-P*$_{BAD}$ *xabC*_C1A1T1$_{1/2}$_*ambS*_T1$_{1/2}$C/E2A2T2$_{1/2}$_*xtpS*_T2$_{1/2}$C3A3T3$_{1/2}$_*xabC*_T3$_{1/2}$C4A4T4TeTe *tacI* and *araE*	This work

NRPS-	Plasmids	Genotype	Reference
**A4_D7_D20**	pAP119_45_55	ori p15A, *cm*^{R}, *araC-P*$_{BAD}$ *xab*C_C1A1T1$_{1/2}$_*ambS*_T1$_{1/2}$C/E2A2T2$_{1/2}$_*xabA*_T3$_{1/2}$C4A4T4$_{1/2}$_*xabC*_T3$_{1/2}$C4A4T4TeTe *tacI* and *araE*	This work
**A4_D7_D21**	pAP119_45_56	ori p15A, *cm*^{R}, *araC-P*$_{BAD}$ *xabC*_C1A1T1$_{1/2}$_*ambS*_T1$_{1/2}$C/E2A2T2$_{1/2}$_*xabA*_T1$_{1/2}$C2A2T2$_{1/2}$_*xabC*_T3$_{1/2}$C4A4T4TeTe *tacI* and *araE*	This work
**A4_D8_D16**	pAP119_46_51	ori p15A, *cm*^{R}, *araC-P*$_{BAD}$ *xabC*_C1A1T1$_{1/2}$_*szeS*_T1$_{1/2}$C/E2A2T2$_{1/2}$_*ambS*_T1$_{1/2}$C/E2A2T2$_{1/2}$_*xabC*_T3$_{1/2}$C4A4T4TeTe *tacI* and *araE*	This work
**A4_D8_D17**	pAP119_46_52	ori p15A, *cm*^{R}, *araC-P*$_{BAD}$ *xabC*_C1A1T1$_{1/2}$_*szeS*_T1$_{1/2}$C/E2A2T2$_{1/2}$_*szeS*_T1$_{1/2}$C/E2A2T2$_{1/2}$_*xabC*_T3$_{1/2}$C4A4T4TeTe *tacI* and *araE*	This work
**A4_D8_D18**	pAP119_46_53	ori p15A, *cm*^{R}, *araC-P*$_{BAD}$ *xabC*_C1A1T1$_{1/2}$_*szeS*_T1$_{1/2}$C/E2A2T2$_{1/2}$_*szeS*_T4$_{1/2}$C/E5A5T5$_{1/2}$_*xabC*_T3$_{1/2}$C4A4T4TeTe *tacI* and *araE*	This work
**A4_D8_D19**	pAP119_46_54	ori p15A, *cm*^{R}, *araC-P*$_{BAD}$ *xldS*_C1A1T1$_{1/2}$_*szeS*_T1$_{1/2}$C/E2A2T2$_{1/2}$_*xtpS*_T2$_{1/2}$C3A3T3$_{1/2}$_xabC_T31/2 C4A4T4TeTe *tacI* and *araE*	This work
**A4_D8_D20**	pAP119_46_55	ori p15A, *cm*^{R}, *araC-P*$_{BAD}$ *xabC*_C1A1T1$_{1/2}$_*szeS*_T1$_{1/2}$C/E2A2T2$_{1/2}$_*xabA*_T3$_{1/2}$C4A4T4$_{1/2}$_*xabC*_T3$_{1/2}$ C4A4T4TeTe *tacI* and *araE*	This work
**A4_D8_D21**	pAP119_46_56	ori p15A, *cm*^{R}, *araC-P*$_{BAD}$ *xabC*_C1A1T1$_{1/2}$_*szeS*_T1$_{1/2}$C/E2A2T2$_{1/2}$_*xabA*_T1$_{1/2}$C2A2T2$_{1/2}$_*xabC*_T3$_{1/2}$ C4A4T4TeTe *tacI* and *araE*	This work
**A4_D9_D16**	pAP119_47_51	ori p15A, *cm*^{R}, *araC-P*$_{BAD}$ *xabC_C1A1T11/2_szeS*_T4$_{1/2}$C/E5A5T5$_{1/2}$_*ambS*_T1$_{1/2}$C/E2A2T2$_{1/2}$_xabC_T31/2C4A4T4TeTe *tacI* and *araE*	This work
**A4_D9_D17**	pAP119_47_52	ori p15A, *cm*^{R}, *araC-P*$_{BAD}$ *xabC*_C1A1T1$_{1/2}$_*szeS*_T4$_{1/2}$C/E5A5T5$_{1/2}$_*szeS*_T1$_{1/2}$C/E2A2T2$_{1/2}$_*xabC*_T3$_{1/2}$C4A4T4TeTe *tacI* and *araE*	This work
**A4_D9_D18**	pAP119_47_53	ori p15A, *cm*^{R}, *araC-P*$_{BAD}$ *xabC*_C1A1T1$_{1/2}$_*szeS*_T4$_{1/2}$C/E5A5T5$_{1/2}$_*szeS*_T4$_{1/2}$C/E5A5T5$_{1/2}$_*xabC*_T3$_{1/2}$C4A4T4TeTe *tacI* and *araE*	This work
**A4_D9_D19**	pAP119_47_54	ori p15A, *cm*^{R}, *araC-P*$_{BAD}$ *xabC*_C1A1T1$_{1/2}$_*szeS*_T4$_{1/2}$C/E5A5T5$_{1/2}$_*xtpS*_T2$_{1/2}$C3A3T3$_{1/2}$_*xabC*_T3$_{1/2}$ C4A4T4TeTe *tacI* and *araE*	This work

NRPS-	Plasmids	Genotype	Reference
**A4_D9_D20**	pAP119_47_55	ori p15A, *cm^R*, *araC-P_{BAD}* *xabC*_C1A1T1$_{1/2}$_*szeS*_T4$_{1/2}$C/E5A5T5$_{1/2}$_*xabA*_T3$_{1/2}$C4A4T4$_{1/2}$_*xabC*_T3$_{1/2}$C4A4T4TeTe *tacI* and *araE*	This work
**A4_D9_D21**	pAP119_47_56	ori p15A, *cm^R*, *araC-P_{BAD}* *xabC*_C1A1T1$_{1/2}$_*szeS*_T4$_{1/2}$C/E5A5T5$_{1/2}$_*xabA*_T1$_{1/2}$C2A2T2$_{1/2}$_*xabC*_T3$_{1/2}$C4A4T4TeTe *tacI* and *araE*	This work
**A4_D10_D16**	pAP119_48_51	ori p15A, *cm^R*, *araC-P_{BAD}* *xabC*_C1A1T1$_{1/2}$_*xtpS*_T2$_{1/2}$C3A3T3$_{1/2}$_*ambS*_T1$_{1/2}$C/E2A2T2$_{1/2}$_*xabC*_T3$_{1/2}$C4A4T4TeTe *tacI* and *araE*	This work
**A4_D10_D17**	pAP119_48_52	ori p15A, *cm^R*, *araC-P_{BAD}* *xabC*_C1A1T1$_{1/2}$_*xtpS*_T2$_{1/2}$C3A3T3$_{1/2}$_*szeS*_T1$_{1/2}$C/E2A2T2$_{1/2}$_*xabC*_T3$_{1/2}$C4A4T4TeTe *tacI* and *araE*	This work
**A4_D10_D18**	pAP119_48_53	ori p15A, cmR, araC-PBAD *xabC*_C1A1T1$_{1/2}$_xtpS_T21/2C3A3T31/2_szeS_T41/2C/E5A5T51/2_*xabC*_T3$_{1/2}$C4A4T4TeTe tacI and araE	This work
**A4_D10_D19**	pAP119_48_54	ori p15A, *cm^R*, *araC-P_{BAD}* *xabC*_C1A1T1$_{1/2}$_*xtpS*_T2$_{1/2}$C3A3T3$_{1/2}$_*xtpS*_T2$_{1/2}$C3A3T3$_{1/2}$_*xabC*_T3$_{1/2}$C4A4T4TeTe *tacI* and *araE*	This work
**A4_D10_D20**	pAP119_48_55	ori p15A, *cm^R*, *araC-P_{BAD}* *xabC*_C1A1T1$_{1/2}$_*xtpS*_T2$_{1/2}$C3A3T3$_{1/2}$_*xabA*_T3$_{1/2}$C4A4T4$_{1/2}$_*xabC*_T3$_{1/2}$C4A4T4TeTe *tacI* and *araE*	This work
**A4_D10_D21**	pAP119_48_56	ori p15A, *cm^R*, *araC-P_{BAD}* *xabC*_C1A1T1$_{1/2}$_*xtpS*_T2$_{1/2}$C3A3T3$_{1/2}$_*xabA*_T1$_{1/2}$C2A2T2$_{1/2}$_*xabC*_T3$_{1/2}$C4A4T4TeTe *tacI* and *araE*	This work
**A4_D11_D16**	pAP119_49_51	ori p15A, *cm^R*, *araC-P_{BAD}* *xabC*_C1A1T1$_{1/2}$_*xabA*_T3$_{1/2}$C4A4T4$_{1/2}$_*ambS*_T1$_{1/2}$C/E2A2T2$_{1/2}$_*xabC*_T3$_{1/2}$C4A4T4TeTe *tacI* and *araE*	This work
**A4_D11_D17**	pAP119_49_52	ori p15A, *cm^R*, *araC-P_{BAD}* *xabC*_C1A1T1$_{1/2}$_*xabA*_T3$_{1/2}$C4A4T4$_{1/2}$_*szeS*_T1$_{1/2}$C/E2A2T2$_{1/2}$_*xabC*_T3$_{1/2}$C4A4T4TeTe *tacI* and *araE*	This work
**A4_D11_D18**	pAP119_49_53	ori p15A, *cm^R*, *araC-P_{BAD}* *xabC*_C1A1T1$_{1/2}$_*xabA*_T3$_{1/2}$C4A4T4$_{1/2}$_*szeS*_T4$_{1/2}$C/E5A5T5$_{1/2}$_*xabC*_T3$_{1/2}$C4A4T4TeTe *tacI* and *araE*	This work
**A4_D11_D19**	pAP119_49_54	ori p15A, *cm^R*, *araC-P_{BAD}* *xabC*_C1A1T1$_{1/2}$_*xabA*_T3$_{1/2}$C4A4T4$_{1/2}$_*xtpS*_T2$_{1/2}$C3A3T3$_{1/2}$_*xabC*_T3$_{1/2}$C4A4T4TeTe *tacI* and *araE*	This work

NRPS-	Plasmids	Genotype	Reference
**A4_D11_D20**	pAP119_49_55	ori p15A, *cm*^{R}, *araC-P*$_{BAD}$ *xabC*_C1A1T1$_{1/2}$_*xabA*_T3$_{1/2}$C4A4T4$_{1/2}$_*xabA*_T3$_{1/2}$C4A4T4$_{1/2}$_*xabC*_T3$_{1/2}$C4A4T4TeTe *tacI* and *araE*	This work
**A4_D11_D21**	pAP119_49_56	ori p15A, *cm*^{R}, *araC-P*$_{BAD}$ *xabC*_C1A1T1$_{1/2}$_*xabA*_T3$_{1/2}$C4A4T4$_{1/2}$_*xabA*_T1$_{1/2}$C2A2T2$_{1/2}$_*xabC*_T3$_{1/2}$C4A4T4TeTe *tacI* and *araE*	This work
**A4_D12_D16**	pAP119_50_51	ori p15A, *cm*^{R}, *araC-P*$_{BAD}$ *xabC*_C1A1T1$_{1/2}$_*xabA*_T1$_{1/2}$C2A2T2$_{1/2}$_*ambS*_T1$_{1/2}$C/E2A2T2$_{1/2}$_*xabC*_T3$_{1/2}$C4A4T4TeTe *tacI* and *araE*	This work
**A4_D12_D17**	pAP119_50_52	ori p15A, *cm*^{R}, *araC-P*$_{BAD}$ *xabC*_C1A1T1$_{1/2}$_*xabA*_T1$_{1/2}$C2A2T2$_{1/2}$_*szeS*_T1$_{1/2}$C/E2A2T2$_{1/2}$_*xabC*_T3$_{1/2}$C4A4T4TeTe *tacI* and *araE*	This work
**A4_D12_D18**	pAP119_50_53	ori p15A, *cm*^{R}, *araC-P*$_{BAD}$ *xabC_C1A1T1*$_{1/2}$_*xabA*_T1$_{1/2}$C2A2T2$_{1/2}$_*szeS*_T4$_{1/2}$C/E5A5T5$_{1/2}$_*xabC*_T3$_{1/2}$C4A4T4TeTe *tacI* and *araE*	This work
**A4_D12_D19**	pAP119_50_54	ori p15A, *cm*^{R}, *araC-P*$_{BAD}$ *xabC*_C1A1T1$_{1/2}$_*xabA*_T1$_{1/2}$C2A2T2$_{1/2}$_*xtpS*_T2$_{1/2}$C3A3T3$_{1/2}$_*xabC*_T3$_{1/2}$C4A4T4TeTe *tacI* and *araE*	This work
**A4_D12_D20**	pAP119_50_55	ori p15A, *cm*^{R}, *araC-P*$_{BAD}$ *xabC*_C1A1T1$_{1/2}$_*xabA*_T1$_{1/2}$C2A2T2$_{1/2}$_*xabA*_T3$_{1/2}$C4A4T4$_{1/2}$_*xabC*_T3$_{1/2}$C4A4T4TeTe *tacI* and *araE*	This work
**A4_D12_D21**	pAP119_50_56	ori p15A, *cm*^{R}, *araC-P*$_{BAD}$ *xabC*_C1A1T1$_{1/2}$_*xabA*_T1$_{1/2}$C2A2T2$_{1/2}$_*xabA*_T1$_{1/2}$C2A2T2$_{1/2}$_*xabC*_T3$_{1/2}$C4A4T4TeTe *tacI* and *araE*	This work

Table S3. List of all NRPS donors created in this work, with their respective GGA overhangs, strain origin, NRPS and module number, amino acids and corresponding plasmid.

Donor	Overhang	Origin strain	NRPS & Module Nr.	Amino acid	Plasmid
**D1**	A/B	*X. miraniensis* DSM 17902	AmbS M2	Glu	pAP39
**D2**	A/B	*X. szentirmaii* DSM 16338	SzeS M2	Thr	pAP40
**D3**	A/B	*X. szentirmaii* DSM 16338	SzeS M5	Tyr	pAP41
**D4**	A/B	*X. nematophila* ATCC 19061	XtpS M3	Val	pAP42

Donor	Overhang	Origin strain	NRPS & Module Nr.	Amino acid	Plasmid
**D5**	A/B	*X. doucetiae* DSM 17909	XabABC M4	Leu	pAP43
**D6**	A/B	*X. doucetiae* DSM 17909	XabABC M2	Ala	pAP44
**D7**	A/C	*X. miraniensis* DSM 17902	AmbS M2	Gln	pAP45
**D8**	A/C	*X. szentirmaii* DSM 16338	SzeS M2	Thr	pAP46
**D9**	A/C	*X. szentirmaii* DSM 16338	SzeS M5	Tyr	pAP47
**D10**	A/C	*X. nematophila* ATCC 19061	XtpS M3	Val	pAP48
**D11**	A/C	*X. doucetiae* DSM 17909	XabABC M4	Leu	pAP49
**D12**	A/C	*X. doucetiae* DSM 17909	XabABC M2	Ala	pAP50
**D13**	A/C	*X. innexi* DSM 16336	XabABC M8	Val	pAP95
**D14**	A/C	*X. beddingii* DSM 4764	XabABC M5	Val	pAP96
**D15**	A/C	*X. innexi* DSM 16336	FitAB M4	Tyr	pAP89
**D16**	C/B	*X. miraniensis* DSM 17902	AmbS M2	Gln	pAP51
**D17**	C/B	*X. szentirmaii* DSM 16338	SzeS M2	Thr	pAP52
**D18**	C/B	*X. szentirmaii* DSM 16338	SzeS M5	Tyr	pAP53
**D19**	C/B	*X. nematophila* ATCC 19061	XtpS M3	Val	pAP54
**D20**	C/B	*X. doucetiae* DSM 17909	XabABC M4	Leu	pAP55
**D21**	C/B	*X. doucetiae* DSM 17909	XabABC M2	Ala	pAP56
**D22**	C/B	*X. innexi* DSM 16336	XabABC M8	Val	pAP98
**D23**	C/B	*X. beddingii* DSM 4764	XabABC M5	Val	pAP99
**D24**	C/B	*X. innexi* DSM 16336	FitAB M4	Tyr	pAP92
**D25**	S/A	*X. indica* DSM 17382	XldS M1	FA-Glu	pAP107
**D26**	S/A	*X. doucetiae* DSM 17909	XabABC M1	FA-Pro	pAP108
**D27**	S/A	*X. miraniensis* DSM 17902	AmbS M1	Ser	pAP109
**D28**	S/A	*X. ishibashii* DSM 22670	LC536431 M1	Leu	pAP110
**D29**	A/B	*X. doucetiae* DSM 17909	XabABC M11	β-Ala	pAP113
**D30**	A/B	*X. doucetiae* DSM 17909	XabABC M12	Pro	pAP114

Donor	Overhang	Origin strain	NRPS & Module Nr.	Amino acid	Plasmid
**D31**	A/C	*X. doucetiae* DSM 17909	XabABC M11	β-Ala	pAP115
**D32**	A/C	*X. doucetiae* DSM 17909	XabABC M12	Pro	pAP116
**D33**	C/B	*X. doucetiae* DSM 17909	XabABC M11	β-Ala	pAP117
**D34**	C/B	*X. doucetiae* DSM 17909	XabABC M12	Pro	pAP118

Table S4. Primer and template used in this work to generate the indicated plasmids. The sizes of the PCR products are shown below the template.

**Plasmids**	**Oligo-nucleotide**	**Sequence (5' → 3')**	**Template Product size in bp**
**pAP32**	AP63	TTTTTGGGCTAACAGGAGGAATTCCATG AATATGACACGTAACCATACAT	*X. innexi* gDNA 3091
	AP64.2	TCCTCCAAGCTCAAAGAAATGATCG	
	AP98	CGATCATTTCTTTGAGCTTGGAGGACAC TCCTTGTTGGCGGTAAAG	*X. szentirmaii* gDNA 3343
	AP107	CCGTACCGCCAGCAACGAATGACCACC CAGGGTAAAGAAGTTATCATGC	
	AP105	TGGTCATTCGTTGCTGGCGG	*X. szentirmaii* gDNA 4199
	AP106	GGTGGCAGCAGCCTAGGTTAATTAATTA AATATTATTTACTATATTGTATTCCTCTGT ACC	
	AP61	TTAATTAACCTAGGCTGCTGCCACCGCT GA	pCK_0434 3693
	AP62	GGAATTCCTCCTGTTAGCCCAAAAAAAC GG	
**pAP33**	AP63	TTTTTGGGCTAACAGGAGGAATTCCATG AATATGACACGTAACCATACAT	*X. innexi* gDNA 3091
	AP64.2	TCCTCCAAGCTCAAAGAAATGATCG	
	AP100	CGATCATTTCTTTGAGCTTGGAGGACAC TCGCTATTGATCGTGCAG	*X. miraniensis* gDNA 3271
	AP104	CCGTACCGCCAGCAACGAATGACCACC CAAGGCAAAGAAGCTG	
	AP105	TGGTCATTCGTTGCTGGCGG	*X. szentirmaii* gDNA 4199
	AP106	GGTGGCAGCAGCCTAGGTTAATTAATTA AATATTATTTACTATATTGTATTCCTCTGT ACC	
	AP61	TTAATTAACCTAGGCTGCTGCCACCGCT GA	pCK_0434 3693
	AP62	GGAATTCCTCCTGTTAGCCCAAAAAAAC GG	
**pAP34**	AP63	TTTTTGGGCTAACAGGAGGAATTCCATG AATATGACACGTAACCATACAT	*X. innexi* gDNA 3091
	AP64.2	TCCTCCAAGCTCAAAGAAATGATCG	
	AP98	CGATCATTTCTTTGAGCTTGGAGGACAC TCCTTGTTGGCGGTAAAG	*X. szentirmaii* gDNA 3343
	AP107	CCGTACCGCAAGCAACGAATGACCACC CAGGGTAAAGAAGTTATCATGC	
	AP108	TGGTCATTCGTTGCTTGCGGTACG	*P. laumondii* TTO1 gDNA 4232
	AP109	GGTGGCAGCAGCCTAGGTTAATTAATCA CAGCGCCTCCGC	
	AP61	TTAATTAACCTAGGCTGCTGCCACCGCT GA	pCK_0434 3693

Plasmids	Oligo-nucleotide	Sequence (5' → 3')	Template Product size in bp
	AP62	GGAATTCCTCCTGTTAGCCCAAAAAAACGG	
**pAP35**	AP63	TTTTTGGGCTAACAGGAGGAATTCCATGAATATGACACGTAACCATACAT	*X. innexi* gDNA 3091
	AP64.2	TCCTCCAAGCTCAAAGAAATGATCG	
	AP100	CGATCATTTCTTTGAGCTTGGAGGACACTCGCTATTGATCGTGCAG	*X. miraniensis* gDNA 3271
	AP110	CCGTACCGCAAGCAACGAATGACCACCCAAGGCAAAGAAGCTG	
	AP108	TGGTCATTCGTTGCTTGCGGTACG	*P. laumondii* TTO1 gDNA 4232
	AP109	GGTGGCAGCAGCCTAGGTTAATTAATCACAGCGCCTCCGC	
	AP61	TTAATTAACCTAGGCTGCTGCCACCGCTGA	pCK_0434 3693
	AP62	GGAATTCCTCCTGTTAGCCCAAAAAAACGG	
**pAP36**	AP63	TTTTTGGGCTAACAGGAGGAATTCCATGAATATGACACGTAACCATACAT	*X. innexi* gDNA 3091
	AP64.2	TCCTCCAAGCTCAAAGAAATGATCG	
	AP111	CGATCATTTCTTTGAGCTTGGAGGACATTCCTTGATGGCGGT	*X. szentirmaii* gDNA 7433
	AP106	GGTGGCAGCAGCCTAGGTTAATTAATTAAATATTATTTACTATATTGTATTCCTCTGTACC	
	AP61	TTAATTAACCTAGGCTGCTGCCACCGCTGA	pCK_0434 3693
	AP62	GGAATTCCTCCTGTTAGCCCAAAAAAACGG	
**pAP36b**	AP283	GCCCTGGGTGGTCATTCGTT	pAP36 5168
	AP46	ATGGAGAAAAAAATCACTGGATATACCACC	
	AP45	GGTGGTATATCCAGTGATTTTTTTCTCC	pAP36 9024
	AP284	AACGAATGACCACCCAGGGC	
**pAP37**	AP63	TTTTTGGGCTAACAGGAGGAATTCCATGAATATGACACGTAACCATACAT	*X. indica* gDNA 3120
	AP118	ACCATGAGACCTTGCATTCGTTGGTCTCGTCCTCCAAGCTCAAAGAAATGATCG	
	AP115	AGGACGAGACCAACGAATGCAAGGTCTCATGGTCATTCGTTGCTTGCGG	*P. laumondii* TTO1 gDNA 4261
	AP109	GGTGGCAGCAGCCTAGGTTAATTAATCACAGCGCCTCCGC	
	AP61	TTAATTAACCTAGGCTGCTGCCACCGCTGA	pCK_0434 3693
	AP62	GGAATTCCTCCTGTTAGCCCAAAAAAACGG	

Plasmids	Oligo-nucleotide	Sequence (5’ → 3’)	Template Product size in bp
**pAP38**	AP63	TTTTTGGGCTAACAGGAGGAATTCCATG AATATGACACGTAACCATACAT	*X. indica* gDNA 3120
	AP118	ACCATGAGACCTTGCATTCGTTGGTCTC GTCCTCCAAGCTCAAAGAAATGATCG	
	AP117	AGGACGAGACCAACGAATGCAAGGTCT CATGGTCATTCGTTGCTGGCG	*X. indica* gDNA 4228
	AP106	GGTGGCAGCAGCCTAGGTTAATTAATTA AATATTATTTACTATATTGTATTCCTCTGT ACC	
	AP61	TTAATTAACCTAGGCTGCTGCCACCGCT GA	pCK_0434 3693
	AP62	GGAATTCCTCCTGTTAGCCCAAAAAAAC GG	
**pAP39**	AP121	CACACAGGAAAGAAGGTCTCAAGGACA CTCGCTATTGATCGTGCAGT	*X. miraniensis* gDNA 3271
	AP122	CAGTCACGACCTTTGGTCTCTACCACCC AAGGCAAAGAAGCTGTC	
	AP119	TGGTAGAGACCAAAGGTCGTGACTGGG AAAACCC	pSEVA681 2351
	AP120	TCCTTGAGACCTTCTTTCCTGTGTGAAAT TGTTATCCGCT	
**pAP40**	AP123	CACACAGGAAAGAAGGTCTCAAGGACA CTCCTTGTTGGCGGTAAAG	*X. szentirmaii* gDNA 3343
	AP124	CAGTCACGACCTTTGGTCTCTACCACCC AGGGTAAAGAAGTTATCATGC	
	AP119	TGGTAGAGACCAAAGGTCGTGACTGGG AAAACCC	pSEVA681 2351
	AP120	TCCTTGAGACCTTCTTTCCTGTGTGAAAT TGTTATCCGCT	
**pAP41**	AP125	CACACAGGAAAGAAGGTCTCAAGGACAT TCCTTGATGGCGGT	*X. szentirmaii* gDNA 3259
	AP126	CAGTCACGACCTTTGGTCTCTACCACCC AGGGCAAAGAAATTATC	
	AP119	TGGTAGAGACCAAAGGTCGTGACTGGG AAAACCC	pSEVA681 2351
	AP120	TCCTTGAGACCTTCTTTCCTGTGTGAAAT TGTTATCCGCT	
**pAP42**	AP128	CACACAGGAAAGAAGGTCTCAAGGACAT TCTCTGTTAGCGGTACG	*X. nematophila* gDNA 3229
	AP129	CAGTCACGACCTTTGGTCTCTACCACCC AAAGCAAAGAAACTGTCA	
	AP119	TGGTAGAGACCAAAGGTCGTGACTGGG AAAACCC	pSEVA681 2351
	AP120	TCCTTGAGACCTTCTTTCCTGTGTGAAAT TGTTATCCGCT	
**pAP43**	AP130	CACACAGGAAAGAAGGTCTCAAGGACAT TCATTGCTTGCTGTCCA	

Plasmids	Oligo-nucleotide	Sequence (5’ → 3’)	Template Product size in bp
	AP131	CAGTCACGACCTTTGGTCTCTACCACCC AATTCAAAGAAATGATCATGG	*X. doucetiae* gDNA 3265
	AP119	TGGTAGAGACCAAAGGTCGTGACTGGG AAAACCC	pSEVA681 2351
	AP120	TCCTTGAGACCTTCTTTCCTGTGTGAAAT TGTTATCCGCT	
**pAP44**	AP132	CACACAGGAAAGAAGGTCTCAAGGACA CTCACTGCTGGCTGTC	*X. doucetiae* gDNA 3217
	AP133	CAGTCACGACCTTTGGTCTCTACCACCA AGCTCAAAGAAATGGTCAT	
	AP119	TGGTAGAGACCAAAGGTCGTGACTGGG AAAACCC	pSEVA681 2351
	AP120	TCCTTGAGACCTTCTTTCCTGTGTGAAAT TGTTATCCGCT	
**pAP45**	AP121	CACACAGGAAAGAAGGTCTCAAGGACA CTCGCTATTGATCGTGCAGT	pAP39 3262
	AP154	CCTTTGGTCTCTGCCGCCCAAGGCAAA G	
	AP150	CGGCAGAGACCAAAGGTCGTGACTG	pSEVA681 2351
	AP149	TCCTTGAGACCTTCTTTCCTGTGTG	
**pAP46**	AP123	CACACAGGAAAGAAGGTCTCAAGGACA CTCCTTGTTGGCGGTAAAG	pAP40 3332
	AP162	TTTGGTCTCTGCCGCCCAGGGTAA	
	AP150	CGGCAGAGACCAAAGGTCGTGACTG	pSEVA681 2351
	AP149	TCCTTGAGACCTTCTTTCCTGTGTG	
**pAP47**	AP125	CACACAGGAAAGAAGGTCTCAAGGACAT TCCTTGATGGCGGT	pAP41 3248
	AP164	TTTGGTCTCTGCCGCCCAGGGCAA	
	AP150	CGGCAGAGACCAAAGGTCGTGACTG	pSEVA681 2351
	AP149	TCCTTGAGACCTTCTTTCCTGTGTG	
**pAP48**	AP128	CACACAGGAAAGAAGGTCTCAAGGACAT TCTCTGTTAGCGGTACG	pAP42 3218
	AP166	TTTGGTCTCTGCCGCCCAAAGCAA	
	AP150	CGGCAGAGACCAAAGGTCGTGACTG	pSEVA681 2351
	AP149	TCCTTGAGACCTTCTTTCCTGTGTG	
**pAP49**	AP130	CACACAGGAAAGAAGGTCTCAAGGACAT TCATTGCTTGCTGTCCA	pAP43 3254
	AP168	TTTGGTCTCTGCCGCCGAGTTCAA	
	AP150	CGGCAGAGACCAAAGGTCGTGACTG	pSEVA681 2351
	AP149	TCCTTGAGACCTTCTTTCCTGTGTG	
**pAP50**	AP132	CACACAGGAAAGAAGGTCTCAAGGACA CTCACTGCTGGCTGTC	pAP44 3217
	AP170	TTTGGTCTCTGCCGCCAAGCTCAA	
	AP150	CGGCAGAGACCAAAGGTCGTGACTG	

Plasmids	Oligo-nucleotide	Sequence (5’ → 3’)	Template Product size in bp
	AP149	TCCTTGAGACCTTCTTTCCTGTGTG	pSEVA681 2351
**pAP51**	AP155	AAGAAGGTCTCACGGCCACTCGCTATTG	pAP39 3262
	AP122	CAGTCACGACCTTTGGTCTCTACCACCC AAGGCAAAGAAGCTGTC	
	AP151	TGGTAGAGACCAAAGGTCGTGACTG	pSEVA681 2351
	AP156	CAATAGCGAGTGGCCGTGAGACCTTCTT	
**pAP52**	AP138	CACACAGGAAAGAAGGTCTCACGGC	pAP40 3343
	AP152	CAGTCACGACCTTTGGTCTCTACCA	
	AP151	TGGTAGAGACCAAAGGTCGTGACTG	pAP40 2351
	AP139	GCCGTGAGACCTTCTTTCCTGTGTG	
**pAP53**	AP173	GAAGGTCTCACGGCCATTCCTTGA	pAP41 3248
	AP152	CAGTCACGACCTTTGGTCTCTACCA	
	AP151	TGGTAGAGACCAAAGGTCGTGACTG	pAP41 2361
	AP174	TCAAGGAATGGCCGTGAGACCTTC	
**pAP54**	AP175	GAAGGTCTCACGGCCATTCTCTGT	pAP42 3218
	AP152	CAGTCACGACCTTTGGTCTCTACCA	
	AP151	TGGTAGAGACCAAAGGTCGTGACTG	pAP42 2361
	AP176	ACAGAGAATGGCCGTGAGACCTTC	
**pAP55**	AP177	GAAGGTCTCACGGCCACTCATTGC	pAP43 3254
	AP152	CAGTCACGACCTTTGGTCTCTACCA	
	AP151	TGGTAGAGACCAAAGGTCGTGACTG	pAP43 2361
	AP178	GCAATGAGTGGCCGTGAGACCTTC	
**pAP56**	AP179	GAAGGTCTCACGGCCACTCACTGC	pAP44 3206
	AP152	CAGTCACGACCTTTGGTCTCTACCA	
	AP151	TGGTAGAGACCAAAGGTCGTGACTG	pAP44 2361
	AP180	GCAGTGAGTGGCCGTGAGACCTTC	
**pAP89**	AP226	CACACAGGAAAGAAGGTCTCAAGGAGA CTCCATTACCAGTATCCAATTAGT	*X. innexi* gDNA 819
	AP229	GGGGTCTTGAGTCCACCCGATTG	
	AP228	CAATCGGGTGGACTCAAGACCCC	*X. innexi* gDNA 3453
	AP227	CAGTCACGACCTTTGGTCTCTGCCGCC GATACGGAAGAAATTATCTTCGA	
	AP134	CGGCAGAGACCAAAGGTCGTGACTG	pAP45 2351
	AP149	TCCTTGAGACCTTCTTTCCTGTGTG	
**pAP92**	AP234	CACACAGGAAAGAAGGTCTCACGGCGA CTCCATTACCAGTATCCAATTAGT	pAP89 4249
	AP235	CAGTCACGACCTTTGGTCTCTACCACCG ATACGGAAGAAATTATCTTCGA	
	AP151	TGGTAGAGACCAAAGGTCGTGACTG	pAP51 2351
	AP139	GCCGTGAGACCTTCTTTCCTGTGTG	
**pAP95**	AP242	CACACAGGAAAGAAGGTCTCAAGGACA CTCATTGATGGTGGTCCG	

Plasmids	Oligo-nucleotide	Sequence (5’ → 3’)	Template Product size in bp
	AP243	CAGTCACGACCTTTGGTCTCTGCCGCCC AGTTCAAAGAAATGGTCATG	*X. innexi* gDNA 3489
	AP134	CGGCAGAGACCAAAGGTCGTGACTG	pAP45 2351
	AP149	TCCTTGAGACCTTCTTTCCTGTGTG	
**pAP96**	AP244	CACACAGGAAAGAAGGTCTCAAGGACAT TCCCTGCTGGCTATCC	*X. beddingii* gDNA 3286
	AP245	CAGTCACGACCTTTGGTCTCTGCCGCC GAGTTCAAAGAAATGGTCATG	
	AP134	CGGCAGAGACCAAAGGTCGTGACTG	pAP45 2351
	AP149	TCCTTGAGACCTTCTTTCCTGTGTG	
**pAP98**	AP246	CACACAGGAAAGAAGGTCTCACGGCCA CTCATTGATGGTGGTCCG	*X. innexi* gDNA 3489
	AP247	CAGTCACGACCTTTGGTCTCTACCACCC AGTTCAAAGAAATGGTCATG	
	AP151	TGGTAGAGACCAAAGGTCGTGACTG	pAP51 2351
	AP139	GCCGTGAGACCTTCTTTCCTGTGTG	
**pAP99**	AP248	CACACAGGAAAGAAGGTCTCACGGCCA TTCCCTGCTGGCTATCC	*X. beddingii* gDNA 3286
	AP216	CAGTCACGACCTTTGGTCTCTACCACCG AGTTCAAAGAAATGGTCATG	
	AP151	TGGTAGAGACCAAAGGTCGTGACTG	pAP51 2351
	AP139	GCCGTGAGACCTTCTTTCCTGTGTG	
**pAP106**	AP268	CGAGACCAACGAATGCAAGGTCTCAAG GACACTCACTGCTGGCTGTC	pAP37_44 7428
	AP109	GGTGGCAGCAGCCTAGGTTAATTAATCA CAGCGCCTCCGC	
	AP61	TTAATTAACCTAGGCTGCTGCCACCGCT GA	pAP37_44 3721
	AP269	TGAGACCTTGCATTCGTTGGTCTCGCAT GGAATTCCTCCTGTTAGCCCAAAA	
**pAP107**	AP271	CACACAGGAAAGAAGGTCTCACATGAAT ATGACACGTAACCATACATCCTC	pAP37_44 3107
	AP272	GTCACGACCTTTGGTCTCTTCCTCCAAG CTCAAAGAAATGATCG	
	AP273	AGGAAGAGACCAAAGGTCGTGAC	pAP39 2351
	AP274	CATGTGAGACCTTCTTTCCTGTGTG	
**pAP108**	AP275	CACACAGGAAAGAAGGTCTCACATGCCT ATGTCATGCAATGGTATTAACAACG	*X. doucetiae* gDNA 3236
	AP276	GTCACGACCTTTGGTCTCTTCCTCCAAG CTCAAAGAAATGGTCATGG	
	AP273	AGGAAGAGACCAAAGGTCGTGAC	pAP39 2351
	AP274	CATGTGAGACCTTCTTTCCTGTGTG	
**pAP109**	AP277	CACACAGGAAAGAAGGTCTCACATGAAA AATGATAAGGTGATGACTCTGCCAAC	

Plasmids	Oligo-nucleotide	Sequence (5' → 3')	Template Product size in bp
	AP278	GTCACGACCTTTGGTCTCTTCCTCCGAG CGTAAAGAAGTTATCAAACC	*X. miraniensis* gDNA 2981
	AP273	AGGAAGAGACCAAAGGTCGTGAC	pAP39 2351
	AP274	CATGTGAGACCTTCTTTCCTGTGTG	
**pAP110**	AP279	ACAGGAAAGAAGGTCTCACATGCCTATG TCATGCAATAGTAGCAATAATATTAAATT TCC	*X. ishibashii* gDNA 3104
	AP280	GTCACGACCTTTGGTCTCTTCCTCCGAG TTCGAAGAAATGATCGTAACG	
	AP273	AGGAAGAGACCAAAGGTCGTGAC	pAP39 2351
	AP274	CATGTGAGACCTTCTTTCCTGTGTG	
**pAP111**	AP285	TTTTTGGGCTAACAGGAGGAATTCCATG ATAAGGTCAGAAGACATGAACCTC	*X. doucetiae* gDNA 8180
	AP286	CCGACGGAAAATCCACCTGG	
	AP287	CCAGGTGGATTTTCCGTCGG	*X. doucetiae* gDNA 8373
	AP288	GGTGGCAGCAGCCTAGGTTAATTAATCA ACTAATCACAGCCCAAGCC	
	AP61	TTAATTAACCTAGGCTGCTGCCACCGCT GA	pAP37 3693
	AP62	GGAATTCCTCCTGTTAGCCCAAAAAAAC GG	
**pAP112**	AP289	CACGAGTTGGGACTCTTCTATCG	pAP111 6129
	AP292	CATTAATACAGGCCTCTCAAAGAGTG	
	AP291	CACTCTTTGAGAGGCCTGTATTAATG	pAP111 4516
	AP294	GCCAAATCGGTTTCCGTTTCAATC	
	AP293	GATTGAAACGGAAACCGATTTGGC	pAP111 3364
	AP296	CGGAAAGAGTCCGGCTTTCA	
	AP295	TGAAAGCCGGACTCTTTCCG	pAP111 1001
	AP298	CCCTGTTCAGACACCAGAATGG	
	AP297	CCATTCTGGTGTCTGAACAGGG	pAP111 5281
	AP290	CGATAGAAGAGTCCCAACTCGTG	
**pAP113**	AP299	CACACAGGAAAGAAGGTCTCAAGGACAT TCACTGATGATCGTCAGCCTG	*X. doucetiae* gDNA 3400
	AP300	CAGTCACGACCTTTGGTCTCTACCACCA AGTTCAAAGAAGTGATCATGGC	
	AP119	TGGTAGAGACCAAAGGTCGTGACTGGG AAAACCC	pAP39 2336
	AP120	TCCTTGAGACCTTCTTTCCTGTGTGAAAT TGTTATCCGCT	
**pAP114**	AP301	CACACAGGAAAGAAGGTCTCAAGGACA CTCCCTGCTGGCTGTC	*X. doucetiae* gDNA 121
	AP292	CATTAATACAGGCCTCTCAAAGAGTG	
	AP291	CACTCTTTGAGAGGCCTGTATTAATG	

Plasmids	Oligo-nucleotide	Sequence (5' → 3')	Template Product size in bp
	AP302	CAGTCACGACCTTTGGTCTCTACCACCA AGTTCAAAGAAATGGTCATGGC	*X. doucetiae* gDNA 3182
	AP119	TGGTAGAGACCAAAGGTCGTGACTGGG AAAACCC	pAP39 2336
	AP120	TCCTTGAGACCTTCTTTCCTGTGTGAAAT TGTTATCCGCT	
**pAP115**	AP303	CACACAGGAAAGAAGGTCTCAAGGACAT TCACTGATGATCGTCAGCCT	pAP113 3400
	AP304	CAGTCACGACCTTTGGTCTCTGCCGCCA AGTTCAAAGAAGTGATCATGGC	
	AP150	CGGCAGAGACCAAAGGTCGTGACTG	pAP113 2351
	AP149	TCCTTGAGACCTTCTTTCCTGTGTG	
**pAP116**	AP305	CACACAGGAAAGAAGGTCTCAAGGACA CTCCCTGCTGGCTGTC	pAP114 3277
	AP306	CAGTCACGACCTTTGGTCTCTGCCGCCA AGTTCAAAGAAGTGATCATGGC	
	AP150	CGGCAGAGACCAAAGGTCGTGACTG	pAP114 2351
	AP149	TCCTTGAGACCTTCTTTCCTGTGTG	
**pAP117**	AP307	CACACAGGAAAGAAGGTCTCACGGCCA TTCACTGATGATCGTCAGCCT	pAP113 3400
	AP308	CAGTCACGACCTTTGGTCTCTACCACCA AGTTCAAAGAAGTGATCATGGC	
	AP151	TGGTAGAGACCAAAGGTCGTGACTG	pAP113 2351
	AP139	GCCGTGAGACCTTCTTTCCTGTGTG	
**pAP118**	AP309	CACACAGGAAAGAAGGTCTCACGGCCA CTCCCTGCTGGCTGTC	pAP114 3277
	AP310	CAGTCACGACCTTTGGTCTCTACCACCA AGTTCAAAGAAATGGTCATGGC	
	AP151	TGGTAGAGACCAAAGGTCGTGACTG	pAP114 2351
	AP139	GCCGTGAGACCTTCTTTCCTGTGTG	
**pAP119**	AP312	AGGACGAGACCAACGAATGCAAGGTCT CATGGTCACTCGCTGCTGGCCGTC	pAP112 7662
	AP46	ATGGAGAAAAAAATCACTGGATATACCA CC	
	AP45	GGTGGTATATCCAGTGATTTTTTTCTCC	pAP112 6021
	AP312	ACCATGAGACCTTGCATTCGTTGGTCTC GTCCTCCAAGCTCAAAGAAATGGTCATG CC	
**pAP128**	AP351	TTTTTGGGCTAACAGGAGGAATTCCATG CCTATGTCATGCAATGGTATTAAC	*X. doucetiae* gDNA 14520
	AP323	GTGGCATTGAAATCGACCAGTATTTG	
	AP324	CAAATACTGGTCGATTTCAATGCCAC	*X. doucetiae* gDNA 15183
	AP352	AGCGGTGGCAGCAGCCTAGGTTAATTC ATTGATGACTATCTCCGCCTAAC	

Plasmids	Oligo-nucleotide	Sequence (5’ → 3’)	Template Product size in bp
	AP353	ATTAACCTAGGCTGCTGCCAC	pCOLA_ara/tacI 3108
	AP68	GGAATTCCTCCTGTTAGCCCAAAAA	

Table S5. Primer used in this work for the qPCR analysis of the tera-modular NRPS library.

Oligonucleotide	Sequence (5’ → 3’)	qPCR targeted template
SLo5558	ACTTGCACAACATCTGGCTG	pAP37 backbone fw.
SLo5559	CAGAATCCCATCCTGCAACG	pAP37 backbone rv.
SLo5560	TCAACGGCAACATTTCAGGG	pAP45 rv.
SLo5561	TTTGGGCCAATTCTGACAGC	pAP46 rv.
SLo5562	AGGGGACAACGGGTAAATGT	pAP47 rv.
SLo5563	CAGGGCTGTCGGGATATGAT	pAP48 rv.
SLo5564	ACGGATATGGGCCATGAGTT	pAP49 rv.
SLo5565	ATGAGAGTGGCAAGGGTTGA	pAP50 rv.
SLo5566	CATCTGCGTGTTCACCTGAG	pAP51 fw.
SLo5567	ACAAGGGGAGACAGAAACCA	pAP52 fw.
SLo5568	TTGCCCGTCAGATTTATGCG	pAP53 fw.
SLo5569	CATTAGCGGCTATCTGGTGC	pAP54 fw.
SLo5570	ACTCACCATCTCGCTGAACA	pAP55 fw.
SLo5571	CGTTTGGTCGCTTATCTGCA	pAP56 fw.
SLo5912	TCAGTAGCTTTGCCAGACGA	pAP89 rv.
SLo5913	AACAAAGACAGCTACACCGC	pAP92 fw.
SLo5914	ATGAGTTCGGCCTGTTCTGT	pAP95 rv.
SLo5915	TGGCTCGCGTCTATGAATCT	pAP98 fw.

Table S6. Acceptor and donor plasmids used for the GGA assembled plasmids created and used in this work.

Assembled Plasmid	Acceptor Plasmid	Donor Plasmids
pAP37_39	pAP37	pAP39
pAP37_40	pAP37	pAP40
pAP37_41	pAP37	pAP41
pAP37_42	pAP37	pAP42
pAP37_43	pAP37	pAP43
pAP37_44	pAP37	pAP44

Assembled Plasmid	Acceptor Plasmid	Donor Plasmids
pAP38_39	pAP38	pAP39
pAP38_40	pAP38	pAP40
pAP38_41	pAP38	pAP41
pAP38_42	pAP38	pAP42
pAP38_43	pAP38	pAP43
pAP38_44	pAP38	pAP44
pAP37_45_51	pAP37	pAP45, pAP51
pAP37_45_52	pAP37	pAP45, pAP52
pAP37_45_53	pAP37	pAP45, pAP53
pAP37_45_54	pAP37	pAP45, pAP54
pAP37_45_55	pAP37	pAP45, pAP55
pAP37_45_56	pAP37	pAP45, pAP56
pAP37_46_51	pAP37	pAP46, pAP51
pAP37_46_52	pAP37	pAP46, pAP52
pAP37_46_53	pAP37	pAP46, pAP53
pAP37_46_54	pAP37	pAP46, pAP54
pAP37_46_55	pAP37	pAP46, pAP55
pAP37_46_56	pAP37	pAP46, pAP56
pAP37_47_51	pAP37	pAP47, pAP51
pAP37_47_52	pAP37	pAP47, pAP52
pAP37_47_53	pAP37	pAP47, pAP53
pAP37_47_54	pAP37	pAP47, pAP54
pAP37_47_55	pAP37	pAP47, pAP55
pAP37_47_56	pAP37	pAP47, pAP56
pAP37_48_51	pAP37	pAP48, pAP51
pAP37_48_52	pAP37	pAP48, pAP52
pAP37_48_53	pAP37	pAP48, pAP53
pAP37_48_54	pAP37	pAP48, pAP54
pAP37_48_55	pAP37	pAP48, pAP55
pAP37_48_56	pAP37	pAP48, pAP56
pAP37_49_51	pAP37	pAP49, pAP51
pAP37_49_52	pAP37	pAP49, pAP52
pAP37_49_53	pAP37	pAP49, pAP53
pAP37_49_54	pAP37	pAP49, pAP54
pAP37_49_55	pAP37	pAP49, pAP55
pAP37_49_56	pAP37	pAP49, pAP56
pAP37_50_51	pAP37	pAP50, pAP51
pAP37_50_52	pAP37	pAP50, pAP52
pAP37_50_53	pAP37	pAP50, pAP53
pAP37_50_54	pAP37	pAP50, pAP54
pAP37_50_55	pAP37	pAP50, pAP55
pAP37_50_56	pAP37	pAP50, pAP56
pAP37_95_51	pAP37	pAP95, pAP51

Assembled Plasmid	Acceptor Plasmid	Donor Plasmids
pAP37_95_52	pAP37	pAP95, pAP52
pAP37_95_53	pAP37	pAP95, pAP53
pAP37_95_54	pAP37	pAP95, pAP54
pAP37_95_55	pAP37	pAP95, pAP55
pAP37_95_56	pAP37	pAP95, pAP56
pAP37_96_51	pAP37	pAP96, pAP51
pAP37_96_52	pAP37	pAP96, pAP52
pAP37_96_53	pAP37	pAP96, pAP53
pAP37_96_54	pAP37	pAP96, pAP54
pAP37_96_55	pAP37	pAP96, pAP55
pAP37_96_56	pAP37	pAP96, pAP56
pAP37_45_98	pAP37	pAP45, pAP98
pAP37_46_98	pAP37	pAP46, pAP98
pAP37_47_98	pAP37	pAP47, pAP98
pAP37_48_98	pAP37	pAP48, pAP98
pAP37_49_98	pAP37	pAP49, pAP98
pAP37_50_98	pAP37	pAP50, pAP98
pAP37_45_99	pAP37	pAP45, pAP99
pAP37_46_99	pAP37	pAP46, pAP99
pAP37_47_99	pAP37	pAP47, pAP99
pAP37_48_99	pAP37	pAP48, pAP99
pAP37_49_99	pAP37	pAP49, pAP99
pAP37_50_99	pAP37	pAP50, pAP99
pAP37_95_98	pAP37	pAP95, pAP98
pAP37_95_99	pAP37	pAP95, pAP99
pAP37_96_98	pAP37	pAP96, pAP98
pAP37_96_99	pAP37	pAP96, pAP99
pAP37_89_51	pAP37	pAP89, pAP51
pAP37_89_52	pAP37	pAP89, pAP52
pAP37_89_53	pAP37	pAP89, pAP53
pAP37_89_54	pAP37	pAP89, pAP54
pAP37_89_55	pAP37	pAP89, pAP55
pAP37_89_56	pAP37	pAP89, pAP56
pAP37_45_92	pAP37	pAP45, pAP92
pAP37_46_92	pAP37	pAP46, pAP92
pAP37_47_92	pAP37	pAP47, pAP92
pAP37_48_92	pAP37	pAP48, pAP92
pAP37_49_92	pAP37	pAP49, pAP92
pAP37_50_92	pAP37	pAP50, pAP92
pAP37_89_92	pAP37	pAP89, pAP92
pAP37_89_98	pAP37	pAP89, pAP98
pAP37_89_99	pAP37	pAP89, pAP99
pAP37_95_92	pAP37	pAP95, pAP92

Assembled Plasmid	Acceptor Plasmid	Donor Plasmids
pAP37_96_92	pAP37	pAP96, pAP92
pAP106_107	pAP106	pAP107
pAP106_108	pAP106	pAP108
pAP106_109	pAP106	pAP109
pAP106_110	pAP106	pAP110
pAP119_113	pAP119	pAP113
pAP119_114	pAP119	pAP114
pAP119_115_117	pAP119	pAP115, pAP117
pAP119_115_118	pAP119	pAP115, pAP118
pAP119_116_117	pAP119	pAP116, pAP117
pAP119_116_118	pAP119	pAP116, pAP118
pAP119_39	pAP119	pAP39
pAP119_40	pAP119	pAP40
pAP119_41	pAP119	pAP41
pAP119_42	pAP119	pAP42
pAP119_43	pAP119	pAP43
pAP119_44	pAP119	pAP44
pAP119_45_51	pAP119	pAP45, pAP51
pAP119_45_52	pAP119	pAP45, pAP52
pAP119_45_53	pAP119	pAP45, pAP53
pAP119_45_54	pAP119	pAP45, pAP54
pAP119_45_55	pAP119	pAP45, pAP55
pAP119_45_56	pAP119	pAP45, pAP56
pAP119_46_51	pAP119	pAP46, pAP51
pAP119_46_52	pAP119	pAP46, pAP52
pAP119_46_53	pAP119	pAP46, pAP53
pAP119_46_54	pAP119	pAP46, pAP54
pAP119_46_55	pAP119	pAP46, pAP55
pAP119_46_56	pAP119	pAP46, pAP56
pAP119_47_51	pAP119	pAP47, pAP51
pAP119_47_52	pAP119	pAP47, pAP52
pAP119_47_53	pAP119	pAP47, pAP53
pAP119_47_54	pAP119	pAP47, pAP54
pAP119_47_55	pAP119	pAP47, pAP55
pAP119_47_56	pAP119	pAP47, pAP56
pAP119_48_51	pAP119	pAP48, pAP51
pAP119_48_52	pAP119	pAP48, pAP52
pAP119_48_53	pAP119	pAP48, pAP53
pAP119_48_54	pAP119	pAP48, pAP54
pAP119_48_55	pAP119	pAP48, pAP55
pAP119_48_56	pAP119	pAP48, pAP56
pAP119_49_51	pAP119	pAP49, pAP51
pAP119_49_52	pAP119	pAP49, pAP52

Assembled Plasmid	Acceptor Plasmid	Donor Plasmids
pAP119_49_53	pAP119	pAP49, pAP53
pAP119_49_54	pAP119	pAP49, pAP54
pAP119_49_55	pAP119	pAP49, pAP55
pAP119_49_56	pAP119	pAP49, pAP56
pAP119_50_51	pAP119	pAP50, pAP51
pAP119_50_52	pAP119	pAP50, pAP52
pAP119_50_53	pAP119	pAP50, pAP53
pAP119_50_54	pAP119	pAP50, pAP54
pAP119_50_55	pAP119	pAP50, pAP55
pAP119_50_56	pAP119	pAP50, pAP56

Table S7. List of all compounds produced by NRPS related to the hybrid system (NRPS-A1, NRPS-A2 and NRPS-A3) for the first and second donor libraries and the starter library. Expected compounds that were not detected are marked as ND (not detected). Small italic letters indicate *D*- amino acids and small italic bold letters indicate *D*-*allo*-amino acids, respectively.

**NRPS-**	**Compound**	**Peptide sequence**	**MS calculated [M+H]⁺**	**MS detected [M+H]⁺**	**Ion Formula**	**Δppm**
**1**		C14-*q*TW	644.4023	ND		
**2**		C14-*q*QW	671.4127	ND		
**3**		C14-*q****t***L	571.4066	ND		
**4**	**1**	C14-*qq*L	598.4174	598.4166	$C_{30}H_{56}N_5O_7$	1.3
**5**	**2**	C14-*q*YW	706.4174	706.4179	$C_{39}H_{56}N_5O_7$	0.7
	**3**	C12-*q*YW	678.3861	678.3858	$C_{37}H_{52}N_5O_7$	0.3
	**4**	C14-*q*YF	667.4066	667.4065	$C_{37}H_{55}N_4O_7$	0.1
	**5**	C12-*q*YF	639.3752	639.3747	$C_{35}H_{51}N_4O_7$	0.7
	**6**	C14-*q*FW	690.4225	690.4221	$C_{39}H_{56}N_5O_6$	0.5
	**7**	C14-*q*FF	651.4116	651.4114	$C_{37}H_{55}N_4O_6$	0.4
**A2_D1**	**1**	C14-*qq*L	598.4174	598.4169	$C_{30}H_{56}N_5O_7$	0.9
**A2_D2**	**8**	C14-*q****t***L	571.4066	571.4054	$C_{29}H_{54}N_4O_7$	2.0
	**9**	C12-*q****t***L	543.3752	543.3744	$C_{27}H_{51}N_4O_7$	0.9
**A2_D3**	**10**	C14-*qy*L	633.4222	633.4206	$C_{34}H_{57}N_4O_7$	2.5
**A2_D4**		C14-Q*v*L	569.4273	ND		
	**11**	C14-*q*L	470.3589	470.3587	$C_{25}H_{48}N_3O_5$	0.1
**A2_D5**	**12**	C14-Q*l*L	583.4429	583.4429	$C_{31}H_{59}N_4O_6$	0
**A2_D6**	**13**	C14-Q*a*L	541.396	541.3961	$C_{28}H_{53}N_4O_6$	0.3

NRPS-	Compound	Peptide sequence	MS calculated [M+H]$^+$	MS detected [M+H]$^+$	Ion Formula	Δppm
**A1_D1**		C14-*q*QW	671.4127	ND		
**A1_D2**		C14-*q*TW	644.4018	ND		
**A1_D3**	**2**	C14-*q*YW	706.4174	706.4175	$C_{39}H_{56}N_5O_7$	0.1
	**3**	C12-*q*YW	678.3861	678.3858	$C_{37}H_{52}N_5O_7$	0.3
	**4**	C14-*q*YF	667.4066	667.4065	$C_{37}H_{55}N_4O_7$	0.1
	**5**	C12-*q*YF	639.3752	639.3745	$C_{35}H_{51}N_4O_7$	1.2
	**6**	C14-*q*FW	690.4225	690.4219	$C_{39}H_{56}N_5O_6$	0.9
	**7**	C14-*q*FF	651.4116	651.411	$C_{37}H_{55}N_4O_6$	1
**A1_D4**		C14-QVW	642.4225	ND		
**A1_D5**		C14-QLW	656.4382	ND		
**A1_D6**		C14-QAW	614.3912	ND		
**A2_D7_D16**	**14**	C14-*qqq*L	726.476	726.4761	$C_{35}H_{64}N_7O_9$	0.1
**A2_D7_D17**	**15**	C14-*qq****t***L	699.4651	699.4642	$C_{35}H_{64}N_7O_{10}$	1.2
	**8**	C14-*q****t***L	571.4066	571.4059	$C_{29}H_{55}N_4O_7$	1.1
**A2_D7_D18**		C14-*qqy*L	761.4808	ND		
**A2_D7_D19**	**16**	C14-*q*Q*v*L	697.4859	697.4862	$C_{35}H_{65}N_6O_8$	0.5
**A2_D7_D20**		C14-*q*Q*l*L	711.5015	ND		
	**13**	C14-Q*a*L	541.396	541.3957	$C_{28}H_{53}N_4O_6$	0.5
**A2_D7_D21**	**17**	C14-*q*Q*a*L	669.4546	669.4539	$C_{33}H_{61}N_6O_8$	0.9
	**13**	C14-Q*a*L	541.396	541.3958	$C_{28}H_{53}N_4O_6$	0.4
**A2_D8_D16**		C14-*q****t****q*L	699.4651	ND		
	**1**	C14-*qq*L	598.4174	598.4165	$C_{30}H_{56}N_5O_7$	1.6
**A2_D8_D17**	**18**	C14-*q****tt***L	672.4542	672.4535	$C_{33}H_{62}N_5O_9$	1

NRPS-	Compound	Peptide sequence	MS calculated $[M+H]^+$	MS detected $[M+H]^+$	Ion Formula	Δppm
**A2_D8_D18**		C14-*q*__*t*__*y*L	734.4699	ND		
**A2_D8_D19**	**19**	C14-*q*T*v*L	670.475	670.4734	$C_{34}H_{64}N_5O_8$	2.3
**A2_D8_D20**	**20**	C14-*q*T*l*L	684.4906	684.4901	$C_{35}H_{66}N_5O_8$	0.7
**A2_D8_D21**	**21**	C14-*q*T*a*L	642.4437	642.4428	$C_{32}H_{60}N_5O_8$	1.4
**A2_D9_D16**		C14-*qyq*L	761.4808	ND		
	**1**	C14-*qq*L	598.4174	598.4161	$C_{30}H_{56}N_5O_7$	1.7
**A2_D9_D17**		C14-*qy*__*t*__L	734.4699	ND		
	**8**	C14-*q*__*t*__L	571.4066	571.4061	$C_{29}H_{54}N_4O_7$	0.7
**A2_D9_D18**		C14-*qyy*L	796.4855	ND		
**A2_D9_D19**	**22**	C14-*q*Y*v*L	732.4906	732.491	$C_{39}H_{66}N_5O_8$	0.5
**A2_D9_D20**	**23**	C14-*q*Y*l*L	746.5063	746.5056	$C_{40}H_{68}N_5O_8$	0.9
**A2_D9_D21**	**24**	C14-*q*Y*a*L	704.4593	704.459	$C_{37}H_{62}N_5O_8$	0.4
	**25**	C12-*q*Y*a*L	676.428	676.4281	$C_{35}H_{58}N_5O_8$	0.2
**A2_D10_D16**		C14-Q*vq*L	697.4859	ND		
	**1**	C14-*qq*L	598.4174	598.4172	$C_{30}H_{56}N_5O_7$	0.2
**A2_D10_D17**		C14-Q*v*__*t*__L	670.475	ND		
	**8**	C14-*q*__*t*__L	571.4066	571.4051	$C_{29}H_{55}N_4O_7$	2.5
**A2_D10_D18**		C14-Q*vy*L	732.4906	ND		
**A2_D10_D19**		C14-QV*v*L	668.4957	ND		
	**11**	C14-*q*L	470.3589	470.3587	$C_{25}H_{48}N_3O_5$	0.3
**A2_D10_D20**	**26**	C14-QV*l*L	682.5113	682.511	$C_{36}H_{68}N_5O_7$	0.4
	**12**	C14-Q*l*L	583.4429	583.4421	$C_{31}H_{59}N_4O_6$	1.4
**A2_D10_D21**	**27**	C14-QVaL	640.4644	640.4635	$C_{33}H_{62}N_5O_7$	1.4

NRPS-	Compound	Peptide sequence	MS calculated $[M+H]^+$	MS detected $[M+H]^+$	Ion Formula	Δppm
**A2_D11_D16**	**28**	C14-Q*lq*L	711.5015	711.501	$C_{36}H_{67}N_6O_8$	0.6
	**12**	C14-Q*l*L	583.4429	583.4423	$C_{31}H_{59}N_4O_6$	1.1
**A2_D11_D17**	**29**	C14-Q*lt*L	684.4906	684.4898	$C_{35}H_{66}N_5O_8$	1.1
**A2_D11_D18**		C14-Q*ly*L	746.5063	ND		
**A2_D11_D19**		C14-QL*v*L	682.5113	ND		
	**30**	C14Q*v*L	569.4273	569.4258	$C_{30}H_{57}N_4O_6$	2.6
**A2_D11_D20**	**31**	C14-QL*l*L	696.527	696.5269	$C_{37}H_{70}N_5O_7$	0.1
**A2_D11_D21**	**32**	C14-QL*a*L	654.48	654.4797	$C_{34}H_{64}N_5O_7$	0.4
**A2_D12_D16**	**33**	C14-Q*aq*L	669.4546	669.454	$C_{33}H_{61}N_6O_8$	0.8
	**13**	C14-Q*a*L	541.396	541.3954	$C_{28}H_{53}N_4O_6$	1.1
**A2_D12_D17**	**34**	C14-Q*at*L	642.4437	642.4438	$C_{32}H_{60}N_5O_8$	1.3
**A2_D12_D18**		C14-Q*ay*L	704.4593	ND		
**A2_D12_D19**	**35**	C14-QA*v*L	640.4644	640.464	$C_{33}H_{62}N_5O_7$	0.6
	**30**	C14-Q*v*L	569.4273	569.4266	$C_{30}H_{57}N_4O_6$	0.7
**A2_D12_D20**	**36**	C14-QA*l*L	654.48	654.4806	$C_{34}H_{64}N_5O_7$	0.8
	**37**	C12-QA*l*L	626.4487	626.4485	$C_{32}H_{60}N_5O_7$	0.2
**A2_D12_D21**	**38**	C14-QA*a*L	612.4331	612.4326	$C_{31}H_{58}N_5O_7$	0.9
**A2_D13_D16**		C14-Q*vq*L	697.4859	ND		
	**11**	C14-*q*L	470.3589	470.3587	$C_{25}H_{48}N_3O_5$	0.3
**A2_D13_D17**		C14-Q*vt*L	670.475	ND		
	**11**	C14-*q*L	470.3589	470.3585	$C_{25}H_{48}N_3O_5$	0.7
**A2_D13_D18**		C14-Q*vy*L	732.4906	ND		
	**11**	C14-*q*L	470.3589	470.3587	$C_{25}H_{48}N_3O_5$	0.3

NRPS-	Compound	Peptide sequence	MS calculated [M+H]$^+$	MS detected [M+H]$^+$	Ion Formula	Δppm
**A2_D13_D19**		C14-QV*v*L	668.4957	ND		
	**30**	C14-Q*v*L	569.4273	569.4267	$C_{30}H_{57}N_4O_6$	1.1
**A2_D13_D20**	**26**	C14-QV*l*L	682.5113	682.5109	$C_{36}H_{68}N_5O_7$	0.6
	**31**	C14-QL*l*L	696.527	696.5266	$C_{37}H_{70}N_5O_7$	0.6
**A2_D13_D21**	**27**	C14-QV*a*L	640.4644	640.4645	$C_{33}H_{62}N_5O_7$	0.2
	**32**	C14-QL*a*L	654.48	654.4797	$C_{34}H_{64}N_5O_7$	0.6
**A2_D14_D16**		C14-Q*vq*L	697.4859	ND		
	**1**	C14-*qq*L	598.4174	598.417	$C_{30}H_{56}N_5O_7$	0.7
**A2_D14_D17**		C14-Q*vt*L	670.475	ND		
	**8**	C14-*qt*L	571.4066	571.4059	$C_{29}H_{55}N_4O_7$	1.2
**A2_D14_D18**		C14-Q*vy*L	732.4906	ND		
	**11**	C14-*q*L	470.3589	470.3586	$C_{25}H_{48}N_3O_5$	0.6
**A2_D14_D19**		C14-QV*v*L	668.4957	ND		
	**11**	C14-*v*L	470.3589	470.3585	$C_{25}H_{48}N_3O_5$	0.5
**A2_D14_D20**		C14-QV*l*L	682.5113	ND		
	**12**	C14-Q*l*L	583.4429	583.4429	$C_{31}H_{59}N_4O_6$	0.1
**A2_D14_D21**		C14-Q-V*l*L	640.4644	ND		
	**13**	C14-Q*l*L	541.396	541.3956	$C_{28}H_{53}N_4O_6$	0.7
**A2_D7_D22**	**16**	C14-*q*-Q*l*L	697.4859	697.4855	$C_{35}H_{65}N_6O_8$	0.5
	**39**	C14-*q*-Q*l*L	711.5015	711.5011	$C_{36}H_{67}N_6O_8$	0.6
	**30**	C14-Q*v*L	569.4273	569.4266	$C_{30}H_{57}N_4O_6$	1.1
	**12**	C14-Q*l*L	583.4429	583.4418	$C_{31}H_{59}N_4O_6$	1.9
**A2_D8_D22**	**19**	C14-*q*T*v*L	670.475	670.475	$C_{34}H_{64}N_5O_8$	0.1

NRPS-	Compound	Peptide sequence	MS calculated $[M+H]^+$	MS detected $[M+H]^+$	Ion Formula	Δppm
	**20**	C14-*q*T*l*L	684.4906	684.4908	$C_{35}H_{66}N_5O_8$	0.3
	**40**	C12-*q*T*l*L	656.4593	656.4589	$C_{33}H_{62}N_5O_8$	0.6
	**41**	C12-*q*T*v*L	642.4437	642.4432	$C_{32}H_{60}N_5O_8$	0.6
**A2_D9_D22**	**22**	C14-*q*Y*v*L	732.4906	732.4906	$C_{39}H_{66}N_5O_8$	0
	**23**	C14-*q*Y*l*L	746.5063	746.506	$C_{40}H_{68}N_5O_8$	0.3
	**12**	C14-Q*l*L	583.4429	583.4423	$C_{31}H_{59}N_4O_6$	1.3
	**10**	C14-*qy*L	633.4222	633.4217	$C_{34}H_{57}N_4O_7$	0.8
**A2_D10_D22**	**42**	C14-QV*v*L	668.4957	668.4957	$C_{35}H_{66}N_5O_7$	0.1
	**30**	C14-Q*v*L	569.4273	569.4268	$C_{30}H_{57}N_4O_6$	0.9
	**12**	C14-Q*l*L	583.4429	583.4428	$C_{31}H_{59}N_4O_6$	0.2
**A2_D11_D22**	**43**	C14-QL*v*L	682.5113	682.5111	$C_{36}H_{68}N_5O_7$	0.4
	**31**	C14-QL*l*L	696.527	696.5266	$C_{37}H_{70}N_5O_7$	0.5
**A2_D12_D22**	**35**	C14-QA*v*L	640.4644	640.4637	$C_{33}H_{62}N_5O_7$	1.1
	**36**	C14-QA*l*L	654.48	654.4788	$C_{34}H_{64}N_5O_7$	1.9
**A2_D7_D23**	**16**	C14-*q*Q*v*L	697.4859	697.4851	$C_{35}H_{65}N_6O_8$	1
	**30**	C14-Q*v*L	569.4273	569.4265	$C_{30}H_{57}N_4O_6$	1.3
**A2_D8_D23**	**19**	C14-*q*T*v*L	670.475	670.4742	$C_{35}H_{65}N_6O_8$	1.2
**A2_D9_D23**		C14-*q*Y*v*L	732.4906	ND		
**A2_D9_D23**	**10**	C14-*qy*L	633.4222	644.4216	$C_{34}H_{57}N_4O_7$	0.9
**A2_D10_D23**	**42**	C14-QV*v*L	668.4957	668.4954	$C_{35}H_{66}N_5O_7$	0.5
**A2_D11_D23**		C14-QL*v*L	682.5113	ND		
	**10**	C14-*qy*L	633.4222	633.4219	$C_{34}H_{57}N_4O_7$	0.5

NRPS-	Compound	Peptide sequence	MS calculated [M+H]$^+$	MS detected [M+H]$^+$	Ion Formula	Δppm
**A2_D12_D23**	**35**	C14-QA*v*L	640.4644	640.4646	$C_{33}H_{62}N_5O_7$	0.4
	**30**	C14-Q*v*L	569.4273	569.4268	$C_{30}H_{57}N_4O_6$	0.8
**A2_D13_D22**		C14-QV*v*L	668.4957	ND		
	**11**	C14-*q*L	470.3589	470.3579	$C_{25}H_{48}N_3O_5$	2
**A2_D13_D23**	**42**	C14-QV*v*L	668.4957	668.4956	$C_{35}H_{66}N_5O_7$	0.1
	**43**	C14-QL*v*L	682.5113	682.5106	$C_{36}H_{68}N_5O_7$	1
**A2_D14_D22**		C14-QV*v*L	668.4957	ND		
	**30**	C14-Q*v*L	569.4273	569.4272	$C_{30}H_{57}N_4O_6$	0.1
**A2_D14_D23**		C14-QV*v*L	668.4957	ND		
	**30**	C14-Q*v*L	569.4273	569.4273	$C_{30}H_{57}N_4O_6$	0
**A2_D15_D16**		C14-*qyq*L	761.4808	ND	NA	ND
	**11**	C14-*q*L	470.3589	470.3584	$C_{25}H_{48}N_3O_5$	1.1
**A2_D15_D17**		C14-*qyt*L	734.4699	ND		
	**11**	C14-*q*L	470.3589	470.3586	$C_{25}H_{48}N_3O_5$	0.6
**A2_D15_D18**		C14-*qyy*L	796.4855	ND		
**A2_D15_D19**		C14-*q*Y*v*L	732.4906	ND		
	**11**	C14-*q*L	470.3589	470.3585	$C_{25}H_{48}N_3O_5$	0.8
**A2_D15_D20**	**23**	C14-*q*Y*l*L	746.5063	746.5068	$C_{40}H_{68}N_5O_8$	0.8
	**12**	C14-Q*l*L	583.4429	583.4428	$C_{31}H_{59}N_4O_6$	0.3
**A2_D15_D21**	**24**	C14-*q*Y*a*L	704.4593	704.4587	$C_{37}H_{62}N_5O_8$	0.9
	**13**	C14-Q*a*L	541.396	541.3956	$C_{28}H_{53}N_4O_6$	0.7
**A2_D7_D24**		C14-*qqy*L	761.4808	ND		
	**47**	C14-*qy*L	633.4222	633.4217	$C_{34}H_{57}N_4O_7$	0.7

NRPS-	Compound	Peptide sequence	MS calculated [M+H]$^+$	MS detected [M+H]$^+$	Ion Formula	Δppm
**A2_D8_D24**	**44**	C14-*q**t**y*L	734.4699	734.4696	$C_{38}H_{64}N_5O_9$	0.4
	**10**	C14-*qy*L	633.4222	633.4212	$C_{34}H_{57}N_4O_7$	1.5
**A2_D9_D24**	**45**	C14-*qyy*L	796.4855	796.4862	$C_{43}H_{66}N_5O_9$	0.8
	**47**	C14-*qy*L	633.4222	633.4221	$C_{34}H_{57}N_4O_7$	0.2
**A2_D10_D24**		C14-Q*vy*L	732.4906	ND		
	**11**	C14-*q*L	470.3589	470.3583	$C_{25}H_{48}N_3O_5$	1.3
**A2_D11_D24**		C14-Q*ly*L	746.5063	ND		
	**47**	C14-*qy*L	633.4222	633.4213	$C_{34}H_{57}N_4O_7$	1.4
**A2_D12_D24**		C14-Q*ay*L	704.4593	ND		
	**47**	C14-*qy*L	633.4222	633.4213	$C_{34}H_{57}N_4O_7$	1.4
**A2_D15_D24**		C14-*qyy*L	796.4855	ND		
	**47**	C14-*qy*L	633.4222	633.4212	$C_{34}H_{57}N_4O_7$	1.6
	**22**	C14-*q*Y*v*L	732.4906	ND		
**A2_D15_D22**	**30**	C14-Q*v*L	569.4273	569.4261	$C_{30}H_{57}N_4O_6$	2
	**12**	C14-Q*l*L	583.4429	583.4423	$C_{31}H_{59}N_4O_6$	1.1
**A2_D15_D23**	**22**	C14-*q*Y*v*L	732.4906	732.4907	$C_{39}H_{66}N_5O_8$	0.1
	**30**	C14-Q*v*L	569.4273	569.4267	$C_{30}H_{57}N_4O_6$	0.9
	**46**	C14-QVL*v*L	781.5798	781.5797	$C_{41}H_{77}N_6O_8$	0
**A2_D13_D24**		C14-Q*vy*L	732.4906	ND		
	**47**	C14-Q*y*L	633.4222	633.422	$C_{34}H_{57}N_4O_7$	0.2
**A2_D14_D24**		C14-Q*vy*L	732.4906	ND		
	**47**	C14-*qy*L	633.4222	633.4225	$C_{34}H_{57}N_4O_7$	0.4
**A3_D25**	**13**	C14-Q*a*L	541.396	541.3959	$C_{28}H_{53}N_4O_6$	0.2

NRPS-	Compound	Peptide sequence	MS calculated [M+H]$^+$	MS detected [M+H]$^+$	Ion Formula	Δppm
**A3_D26**	**48**	C4-P*a*L	370.2337	370.233	$C_{18}H_{32}N_3O_5$	1.6
	**49**	P*a*L	300.1918	300.1916	$C_{14}H_{26}N_3O_4$	0.7
**A3_D27**	**50**	S*a*L	290.1711	290.1712	$C_{12}H_{24}N_3O_5$	0.4
**A3_D28**	**51**	L*a*L	316.2231	316.2232	$C_{15}H_{30}N_3O_4$	0.5

Table S8. Comparison of the relative production of the compounds 2 – 7 from the NRPS-5, NRPS-5 mod and NRPS-A1_D3. The three constructs produce the same NRPS, while *NRPS-5* was constructed with Gibson assembly, *NRPS-5 mod* is *NRPS-5* after mutagenesis to contain the DNA changes applied with GGA and *NRPS-A1_D3* was constructed using GGA with acceptor and donor plasmids. The titers of compounds **2** – **7** from NRPS-5 (by Gibson assembly) are set to 100 %, to which the titers of the compounds from NRPS-5 mod and NRPS-A1_D3 are compared, respectively.

Compound	Peptide sequence	MS calculated $[M+H]^+$	NRPS-5	NRPS-5 mod	NRPS-A1_D3
**2**	C14-*q*YW	706.4174	100%	105%	97%
**3**	C12-*q*YW	678.3861	100%	115%	99%
**4**	C14-*q*YF	667.4066	100%	103%	80%
**5**	C12-*q*YF	639.3752	100%	95%	82%
**6**	C14-*q*FW	690.4225	100%	121%	91%
**7**	C14-*q*FF	651.4116	100%	102%	82%

Table S9. List of all compounds produced by and related to the xenoamicin library. Expected compounds that were not detected are marked as ND (not detected). The N-terminal linear part C4-P*a*V*I*I is abbreviated as "X". The ring is always formed between Thr-8 and the C-terminal Val-13. Linear products are marked with an "*". The relative production is calculated using the EIC's peak area divided by the area of xenoamicin C (**52**) (NRPS-6; wild type in *E. coli*). Small italic letters indicate *D*-amino acids for the peptide sequence, and small italic bold letters indicate D-*allo*-amino acids.

XabAB+NRPS-	Compound	Peptide sequence	MS calculated $[M+2H]^{2+}$	MS detected $[M+2H]^{2+}$	Ion Formula	Δppm	Rel. Prod.
**6**	**52**	X***t***V***i***V*a*βAPV	657.9235	657.9229	$C_{65}H_{113}N_{13}O_{15}$	0.7	100%
	**53**	X***t***V*I*V*a*βAPV	657.9235	657.9231	$C_{65}H_{113}N_{13}O_{15}$	0.5	7.0%
	**54**	X***t***V*v*V*a*βAPV	650.9156	650.9154	$C_{64}H_{111}N_{13}O_{15}$	0.3	6.3%
**7**	**52**	X***t***V***i***V*a*βAPV	657.9235	657.9233	$C_{65}H_{113}N_{13}O_{15}$	0.2	111%
	**53**	X***t***V*I*V*a*βAPV	657.9235	657.9235	$C_{65}H_{113}N_{13}O_{15}$	0.1	13%
	**54**	X***t***V*v*V*a*βAPV	650.9156	650.9157	$C_{64}H_{111}N_{13}O_{15}$	0.1	6.2%
**A4_D29**	**55**	X***t***V***i***V*a*βAV	609.3971	609.397	$C_{60}H_{106}N_{12}O_{14}$	0.1	6.9%
**A4_D30**	**56**	X***t***V***i***VAPV	622.4049	622.4049	$C_{62}H_{108}N_{12}O_{14}$	0.1	19%
	**57**	X***t***V***i***VAPV*	631.4102	631.4098	$C_{62}H_{110}N_{12}O_{15}$	0.5	10%
**A4_D31_D33**		X***t***V***i***VAAAV	644.9156	ND			
**A4_D31_D34**	**52**	X***t***V***i***V*a*βAPV	657.9235	657.9236	$C_{65}H_{113}N_{13}O_{15}$	0.2	54%
	**53**	X***t***V*I*V*a*βAPV	657.9235	657.9241	$C_{65}H_{113}N_{13}O_{15}$	2	6.1%
	**54**	X***t***V*v*V*a*βAPV	650.9156	650.9158	$C_{64}H_{111}N_{13}O_{15}$	0.3	3.7%
**A4_D32_D33**		X***t***V***i***VA*p*βAV	657.9235	ND			
**A4_D32_D34**		X***t***V***i***VAPPV	670.9313	ND			
**A4_D1**	**58**	X***t***V***i***V*a*QV	637.9078	637.907	$C_{62}H_{109}N_{13}O_{15}$	0.7	0.35%

XabAB+NRPS-	Compound	Peptide sequence	MS calculated $[M+2H]^{2+}$	MS detected $[M+2H]^{2+}$	Ion Formula	Δppm	Rel. Prod.
**A4_D2**	**59**	X***t***V***i***V*a*TV	624.4024	624.4021	$C_{61}H_{108}N_{12}O_{15}$	0.3	6.4%
	**60**	X***t***V***i***V*a*TV*	633.4077	633.4082	$C_{61}H_{110}N_{12}O_{16}$	1	1.8%
**A4_D3**	**61**	X***t***V***i***V*a*YV	655.4102	655.4104	$C_{66}H_{110}N_{12}O_{15}$	0.4	15%
**A4_D4**	**62**	X***t***V***i***VAVV	623.4127	623.4126	$C_{62}H_{110}N_{12}O_{14}$	0.2	4.1%
**A4_D5**	**63**	X***t***V***i***VALV	630.4206	630.4208	$C_{63}H_{112}N_{12}O_{14}$	0.5	17%
	**64**	X***t***V***i***VALV*	639.4258	639.4255	$C_{63}H_{114}N_{12}O_{15}$	0.5	0.69%
**A4_D6**	**65**	X***t***V***i***VAAV	609.3971	609.3973	$C_{60}H_{106}N_{12}O_{14}$	0.5	49%
**A4_D7_D16**		X***t***V***i***V*aq*QV	701.9371	ND			
**A4_D7_D17**		X***t***V***i***V*aq*TV	688.4317	ND			
**A4_D7_D18**		X***t***V***i***V*aq*YV	719.4395	ND			
**A4_D7_D19**		X***t***V***i***V*a*QVV	687.4420	ND			
**A4_D7_D20**		X***t***V***i***V*a*QLV	694.4498	ND			
**A4_D7_D21**		X***t***V***i***V*a*QAV	673.4264	ND			
**A4_D8_D16**		X***t***V***i***V*a****t***QV	688.4317	ND			
**A4_D8_D17**		X***t***V***i***V*a****t***TV	674.9262	ND			
	**59**	X***t***V***i***V*a*TV	624.4024	624.402	$C_{61}H_{108}N_{12}O_{15}$	0.5	2.0%
**A4_D8_D18**		X***t***V***i***V*a****t***YV	705.9340	ND			
	**59**	X***t***V***i***V*a*TV	624.4024	624.402	$C_{61}H_{108}N_{12}O_{15}$	0.5	1.3%
**A4_D8_D19**	**66**	X***t***V***i***V*a*TVV	673.9366	673.9364	$C_{66}H_{117}N_{13}O_{16}$	0.2	3.2%
**A4_D8_D20**	**67**	X***t***V***i***V*a*TLV	680.9444	ND			
	**59**	X***t***V***i***V*a*TV	624.4024	624.4014	$C_{61}H_{108}N_{12}O_{15}$	1.5	0.60%
**A4_D8_D21**	**68**	X***t***V***i***V*a*TAV	659.9209	659.9216	$C_{64}H_{113}N_{13}O_{16}$	1.1	1.1%
	**59**	X***t***V***i***V*a*TV	624.4024	624.403	$C_{61}H_{108}N_{12}O_{15}$	1.2	0.62%

XabAB+NRPS-	Compound	Peptide sequence	MS calculated $[M+2H]^{2+}$	MS detected $[M+2H]^{2+}$	Ion Formula	Δppm	Rel. Prod.
**A4_D9_D16**		X***t***V***i***V*ay*QV	719.4395	ND			
	**58**	X***t***V***i***V*a*QV	637.9078	637.9076	$C_{62}H_{109}N_{13}O_{15}$	0.2	5.0%
**A4_D9_D17**		X***t***V***i***V*ay*TV	705.9340	ND			
	**60**	X***t***V***i***V*a*TV	624.4024	624.4024	$C_{61}H_{108}N_{12}O_{15}$	0.2	6.9%
	**69**	X***t***V***i***VAV	573.8785	573.8785	$C_{57}H_{101}N_{11}O_{13}$	0	3.7%
**A4_D9_D18**		X***t***V***i***V*ay*YV	736.9419	ND			
	**61**	X***t***V***i***V*a*YV	655.4102	655.4102	$C_{66}H_{110}N_{12}O_{15}$	0.1	1.1%
**A4_D9_D19**	**70**	X***t***V***i***V*a*YVV	704.9444	704.9444	$C_{71}H_{119}N_{13}O_{16}$	0.2	1.6%
	**62**	X***t***V***i***VAVV	623.4127	623.412	$C_{62}H_{110}N_{12}O_{14}$	1.1	2.9%
**A4_D9_D20**		X***t***V***i***V*a*YLV	711.9522	ND			
	**63**	X***t***V***i***VALV	630.4206	630.4209	$C_{63}H_{112}N_{12}O_{14}$	0.6	29%
**A4_D9_D21**	**71**	X***t***V***i***V*a*YAV	690.9287	690.9288	$C_{69}H_{115}N_{13}O_{16}$	0.2	4.6%
	**65**	X***t***V***i***VAAV	609.3971	609.3971	$C_{60}H_{106}N_{12}O_{14}$	0.1	1.4%
	**69**	X***t***V***i***VAV	573.8785	573.8787	$C_{57}H_{101}N_{11}O_{13}$	0.4	1.3%
**A4_D10_D16**		X***t***V***i***VA*v*QV	687.4420	ND			
**A4_D10_D17**		X***t***V***i***VA*v*TV	673.9366	ND			
**A4_D10_D18**		X***t***V***i***VA*v*YV	704.9444	ND			
**A4_D10_D19**	**72**	X***t***V***i***VAVVV	672.9469	672.946	$C_{67}H_{119}N_{13}O_{15}$	1.4	0.2%
**A4_D10_D20**	**73**	X***t***V***i***VAVLV	679.9548	679.9545	$C_{68}H_{121}N_{13}O_{15}$	0.2	0.4%
**A4_D10_D21**	**74**	X***t***V***i***VAVAV	658.9313	658.9313	$C_{65}H_{115}N_{13}O_{15}$	0.2	1.5%
**A4_D11_D16**		X***t***V***i***VA*l*QV	694.4498	ND			
	**69**	X***t***V***i***VAV	573.8785	573.8787	$C_{57}H_{101}N_{11}O_{13}$	0.5	3.9%
**A4_D11_D17**		X***t***V***i***VA*l*TV	680.9444	ND			

XabAB+NRPS-	Compound	Peptide sequence	MS calculated $[M+2H]^{2+}$	MS detected $[M+2H]^{2+}$	Ion Formula	Δppm	Rel. Prod.
	**75**	X***t***V***i***VA*a*TV	659.9209	659.921	$C_{64}H_{113}N_{13}O_{16}$	0.2	50%
	**69**	X***t***V***i***VAV	573.8785	573.878	$C_{57}H_{101}N_{11}O_{13}$	0.7	4.4%
**A4_D11_D18**		X***t***V***i***VA*l*YV	711.9522	ND			
	**69**	X***t***V***i***VAV	573.8785	573.8785	$C_{57}H_{101}N_{11}O_{13}$	0.1	6.3%
**A4_D11_D19**		X***t***V***i***VALVV	679.9548	ND			
	**69**	X***t***V***i***VAV	573.8785	573.8785	$C_{57}H_{101}N_{11}O_{13}$	0.1	3.0%
**A4_D11_D20**		X***t***V***i***VALLV	686.9626	ND			
	**76**	X***t***V***i***VALAV	665.9391	665.9391	$C_{66}H_{117}N_{13}O_{15}$	0.1	1.3%
	**69**	X***t***V***i***VAV	573.8785	573.8786	$C_{57}H_{101}N_{11}O_{13}$	0.2	4.1%
**A4_D11_D21**		X***t***V***i***VALAV	665.9391	ND			
	**77**	X***t***V***i***VAAAV	644.9156	644.9157	$C_{63}H_{111}N_{13}O_{15}$	0.2	8.6%
	**69**	X***t***V***i***VAV	573.8785	573.8787	$C_{57}H_{101}N_{11}O_{13}$	0.4	3.7%
**A4_D12_D16**		X***t***V***i***VA*a*QV	673.4264	ND			
	**78**	X***t***V***i***VA*l*QV	694.4498	694.4502	$C_{68}H_{120}N_{14}O_{16}$	0.6	0.68%
**A4_D12_D17**		X***t***V***i***VA*a*TV	659.9209	ND			
**A4_D12_D18**		X***t***V***i***VA*a*YV	690.9287	ND			
**A4_D12_D19**		X***t***V***i***VAAVV	658.9313	ND			
**A4_D12_D20**		X***t***V***i***VAALV	665.9391	ND			
	**63**	X***t***V***i***VALV	630.4206	630.4213	$C_{63}H_{112}N_{12}O_{14}$	1.3	1.3%
**A4_D12_D21**		X***t***V***i***VAAAV	644.9156	ND			
**A4_D12_D21**	**79**	X***t***V***i***VAALV	665.9391	665.9388	$C_{66}H_{117}N_{13}O_{15}$	0.3	1.1%

D. Supplementary Figures

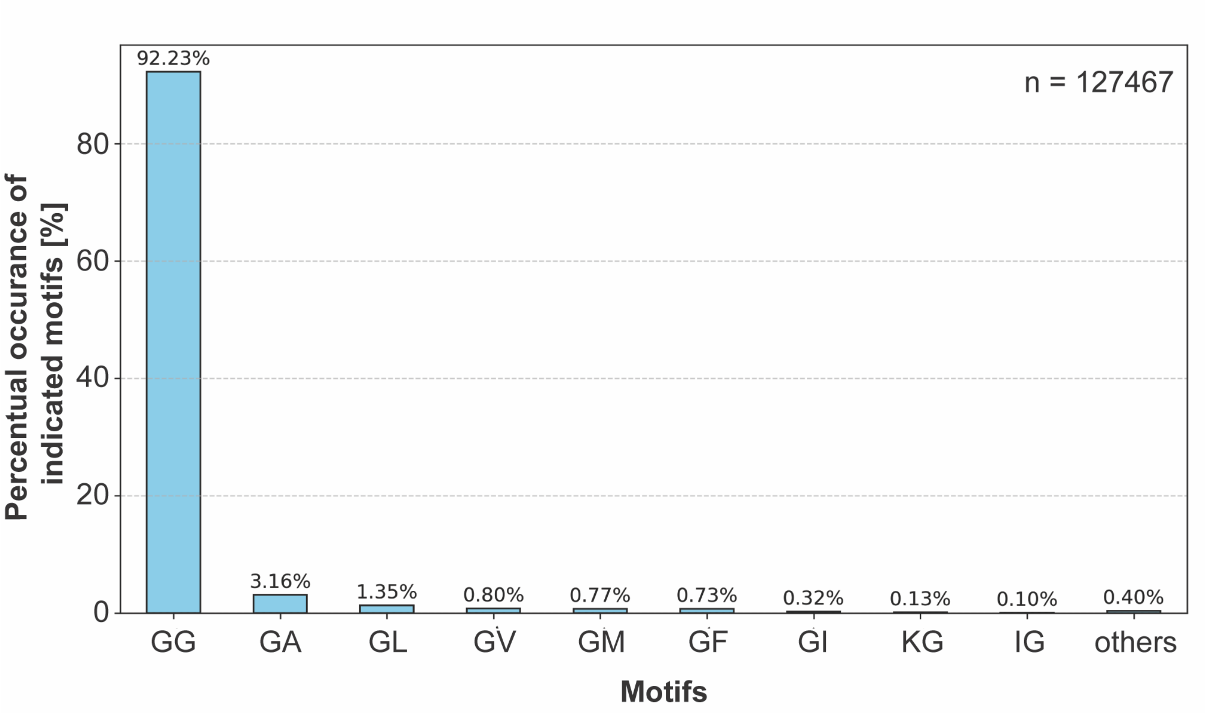

Figure S1. Percentage of occurrence of different motifs identified in the FFxx*GG*xS motif of bacteria. Bar plot of different XX amino acid pairs identified within the conserved FFxxXXxS motif at the position of the GGA targeted conserved glycines. The group "others" comprises all other motifs with an occurrence lower than 0.1%.

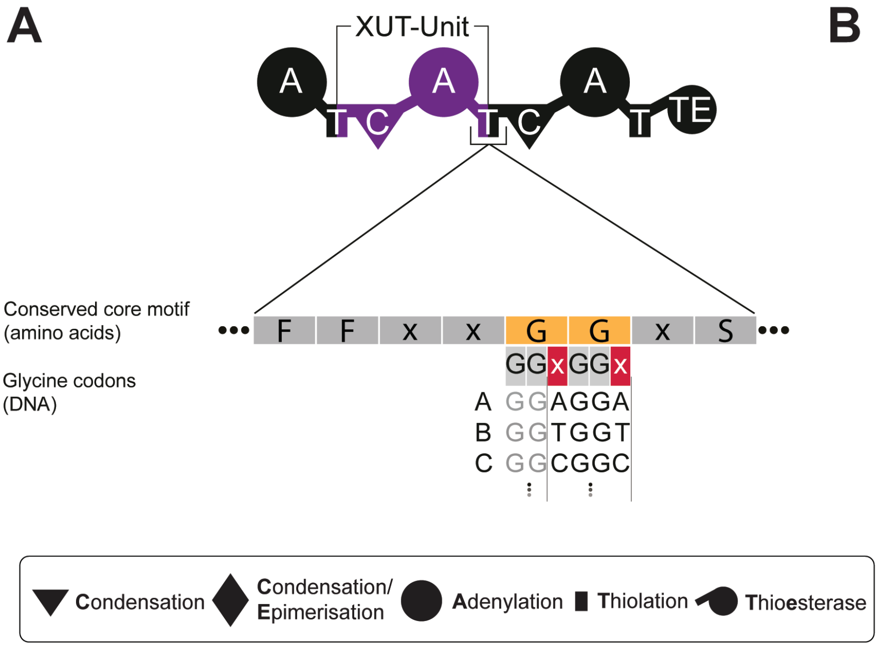

B

Name	Overhang
A	AGGA
B	TGGT
C	CGGC
D	GGGG
E	AGGT
F	TGGC
G	CGGG
H	AGGC
I	TGGG
J	GGGA
K	AGGG
L	CGGA
M	GGGT
N	TGGA
O	CGGT
P	GGGC
S	CATG
T	TGAT

Figure S2. Schematic representation of a basic NRPS showing its architecture as well as the conserved motif for the XUT^{IV} fusions side with its possible GGA overhangs. A) Basic NRPS architecture and the conserved motif for the XUT^{IV} fusion site. Both glycines are marked in orange, providing the codons for the overhangs. The two variable positions for the overhangs are marked in red. **B**) Table for all sixteen possible overhangs, using the flexibility of the third base from the glycine codon. The starter (S) and termination (T) overhangs for the starter and termination modules are also attached.

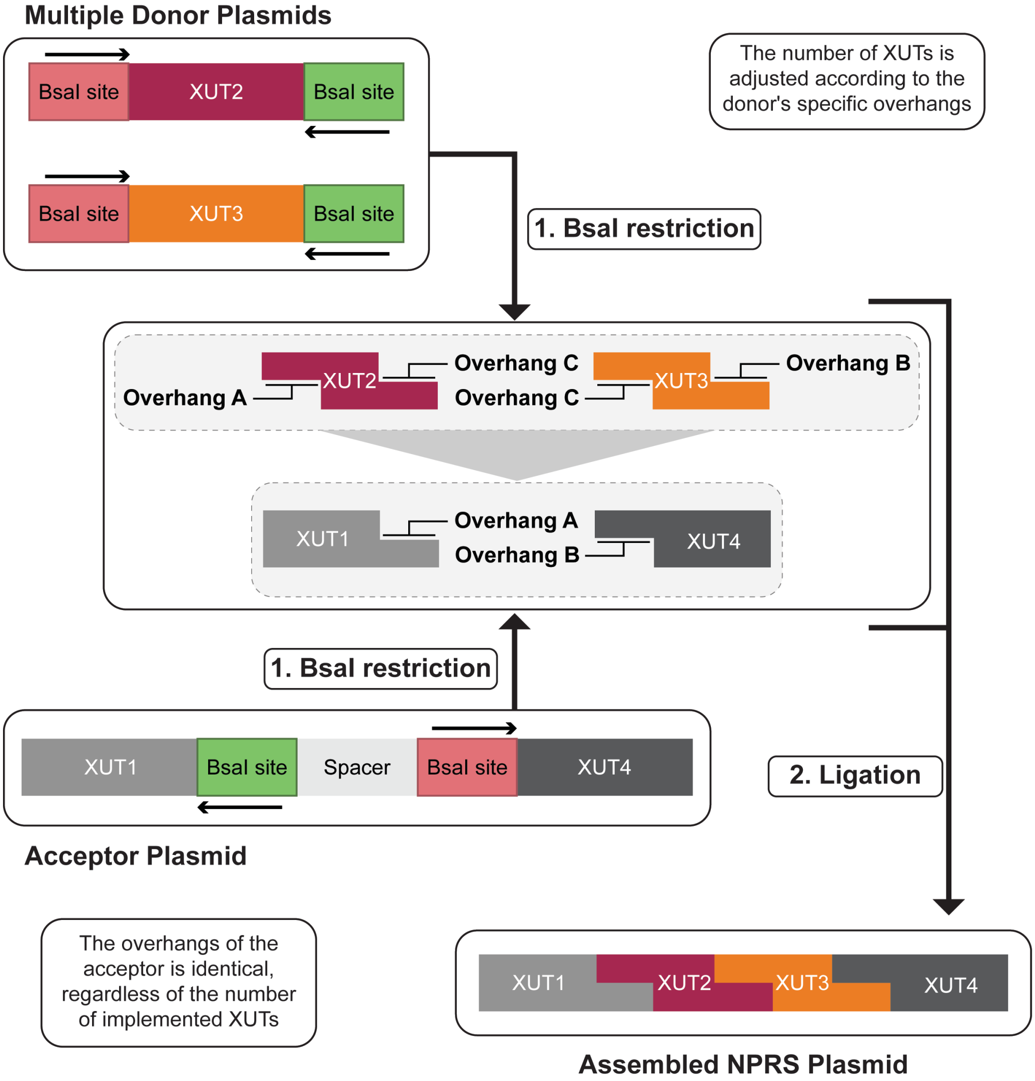

Figure S3. Schematic GGA acceptor and donor system design for XUT-based NRPS engineering on the DNA level. The example refers to implementing two XUT modules. The Donor plasmid sequence design with XUT2 & XUT3 on two plasmids contains two flanking BsaI restriction sites (red and green). The acceptor plasmid sequence design contains XUT1 (light grey) and XUT4 (dark grey), with a spacer sequence between both modules. The BsaI sites are highlighted in green and red, (according to their orientation). The linearised acceptor with overhangs A and B indicated after BsaI restriction, and the cleaved XUT2 & XUT3 DNA fragment with the indicated overhangs A/C and C/B are assembled between XUT1 and XUT4, creating a four-modular NRPS.

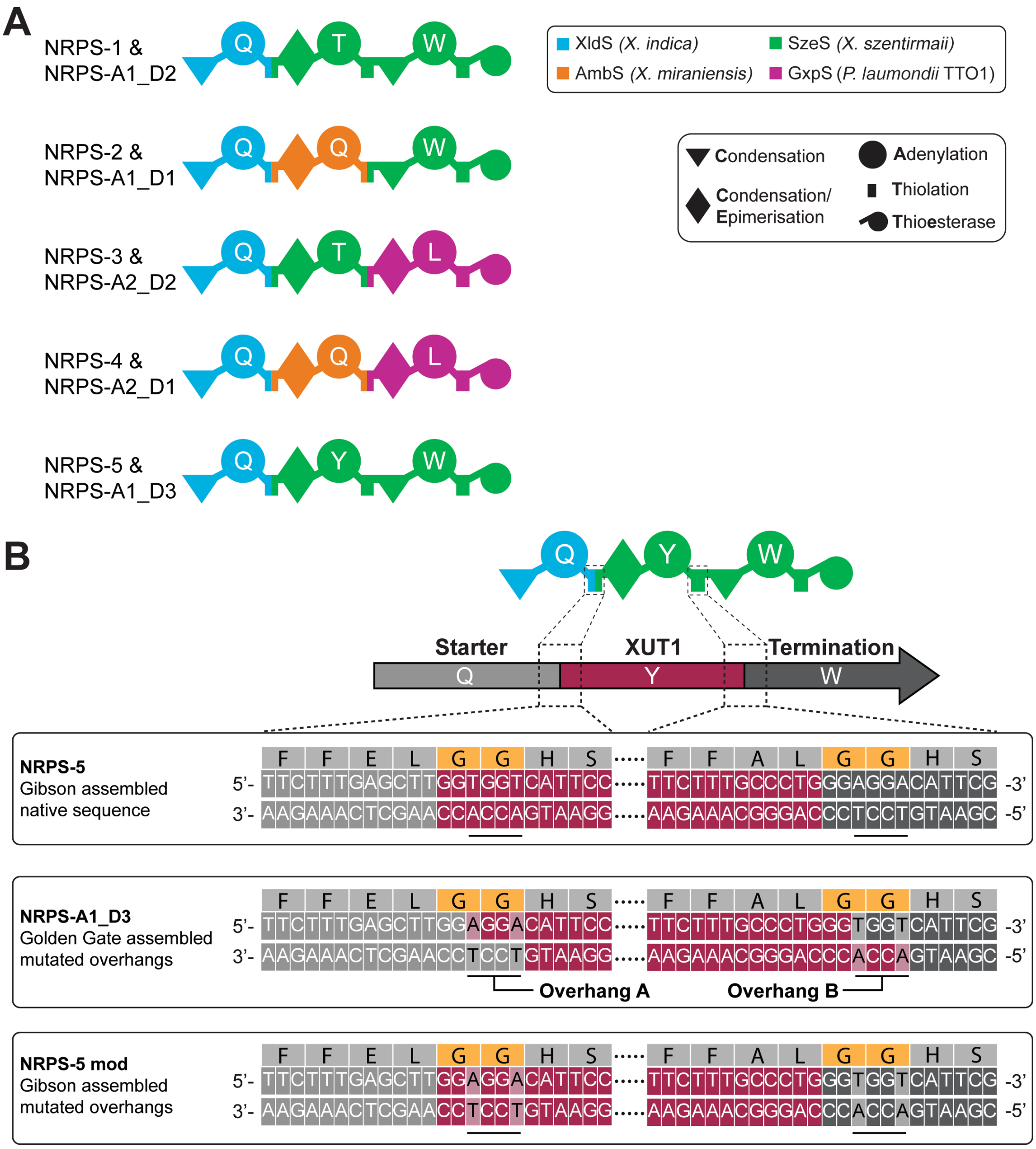

Figure S4. Overview of the controls NRPS-1 to NRPS-5, as well as NRPS-A1_D1 to NRPS-A1_D3 and NRPS-A2_D1 to NRPS-A2_D2, with the sequence comparison of the motif FFxxGGxS for NRPS-5, NRPS-A1_D3 and NRPS-5 mod. **A**) Schematic representation of the *NRPS* constructs NRPS-1 to NRPS-5, created with Gibson assembly, and their respective counterparts, NRPS-A1_D1 to NRPS-A1_D3 and NRPS-A2_D1 to NRPS-A2_D2, generated using GGA. **B**) Sequence comparison of the constructs *NRPS-5*, *NRPS-A1_D3* and *NRPS-5 mod* with focus on the two glycines (orange) of the conserved FFxxGGxS motif. The colour code was adapted from the NRPS colour code to the schematic one shown in Fig. 1A, with a focus on XUT1 (bordeaux red). The overhangs A and B are marked for *NPRS-A1_D3* with the modified sequences highlighted for the GGA construct, as well as the *NRPS-5 mod*.

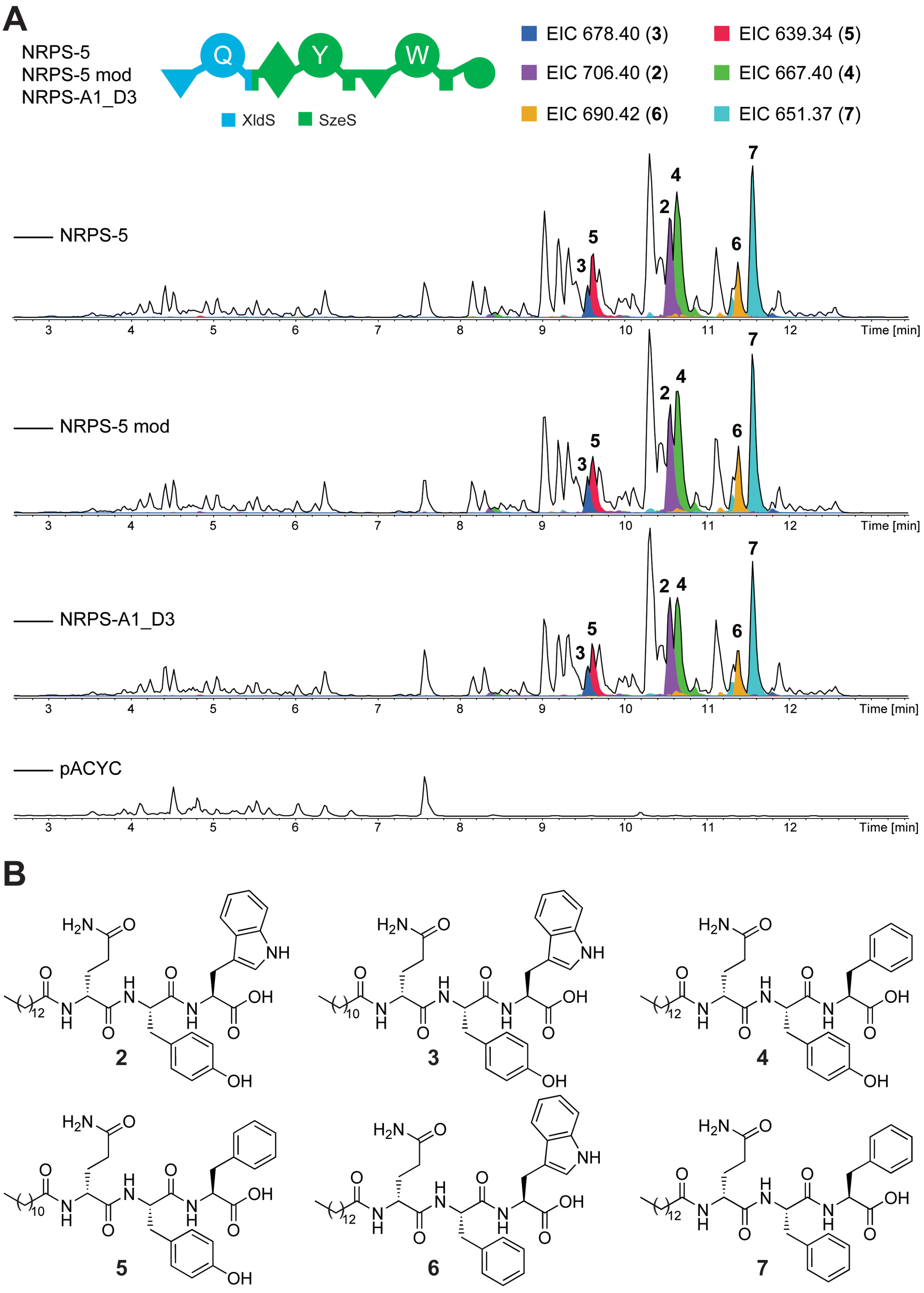

Figure S5. HPLC/MS analysis of the produced NRPS-5, NRPS-5 mod and NRPS-A1_D3 in *E. coli* DH10B::*mtaA*. A) Schematic representation of NRPS-5,

NRPS-5 modified and NRPS-A1_D3 with the colour code of the original NRPS. The A domain specificities are indicated with Q = glutamine, Y = tyrosine and W = tryptophan. All three NRPS produce the same compounds. *NRPS-5* was assembled using Gibson assembly, while *NRPS-A1_D3* was assembled with GGA. *NRPS-5 mod* is a mutated version of *NRPS-5*, where the glycine codons for both fusion sides were changed to represent the ones after GGA (see Fig. S4). HPLC/MS data of compounds **2** – **7** produced in *E. coli* DH10B::*mtaA* expressing *NRPS-5*, *NRPS-5 mod* and *NRPS-A1_D3*. Base peak chromatograms (BPC, black lines) and the extracted ion chromatograms (EIC, below with colours according to the depicted legend) of **2** (*m/z* $[M+H]^+$ = 706.40), **3** (*m/z* $[M+H]^+$ = 678.40), **4** (*m/z* $[M+H]^+$ = 667.40), **5** (*m/z* $[M+H]^+$ = 639.34), **6** (*m/z* $[M+H]^+$ = 690.42), and **7** (*m/z* $[M+H]^+$ = 651.37). The pACYC expression was used as a negative control (dotted line). **B**) Chemical structure of the peptides **2** – **7**.

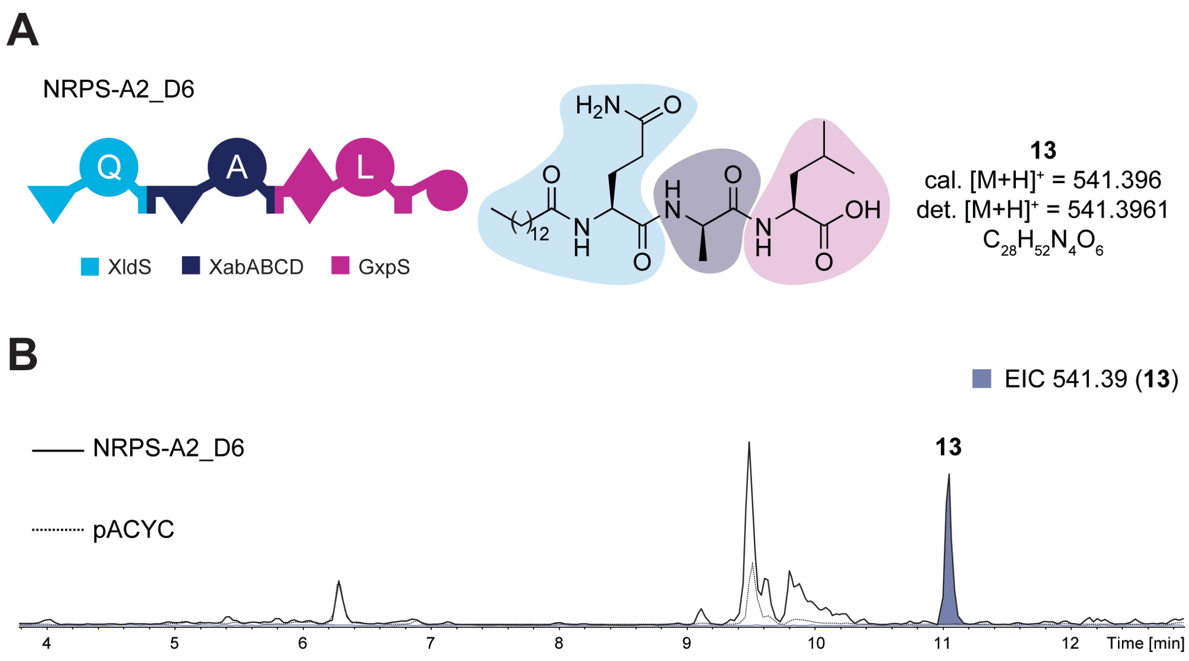

Figure S6. HPLC/MS analysis of the produced NRPS-A2_D6 in *E. coli* DH10B*::mtaA*. A) Schematic representation of NRPS-A2_D6 with the colour code of the original NRPS. The A domain specificities are indicated with Q = glutamine, A = alanine and L = leucine. Chemical structure of the peptide **13** with its molecular formula, the calculated mass and the measured high-resolution mass $[M+H]^+$. **B**) HPLC/MS data of compound **13** produced in *E. coli* DH10B::*mtaA* expressing *NRPS-A2_D6*. Base peak chromatogram (BPC, black line) and the extracted ion chromatogram (EIC, below with colours according to the depicted legend) of **13** (*m/z* $[M+H]^+$ = 541.39). The pACYC expression was used as a negative control (dotted line).

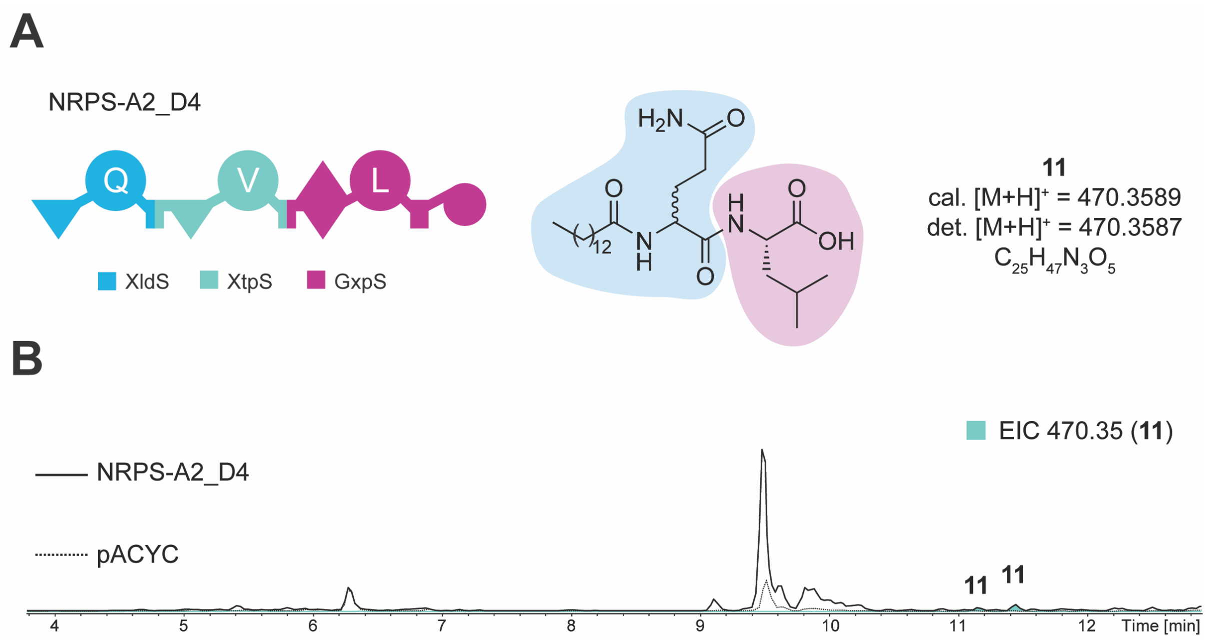

Figure S7. HPLC/MS analysis of the produced NRPS-A2_D4 in *E. coli* DH10B*::mtaA*. A) Schematic representation of NRPS-A2_D4 with the colour code of the original NRPS. The A domain specificities are indicated with Q = glutamine, V = valine and L = leucine. Chemical structure of the peptide **11** with its molecular formula, the calculated mass and the measured high-resolution mass $[M+H]^+$. **B**) HPLC/MS data of compound **11** produced in *E. coli* DH10B::*mtaA* expressing *NRPS-A2_D4*. Base peak chromatogram (BPC, black line) and the extracted ion chromatogram (EIC, below with colours according to the depicted legend) of **11** (*m/z* $[M+H]^+$ = 470.35). The pACYC expression was used as a negative control (dotted line).

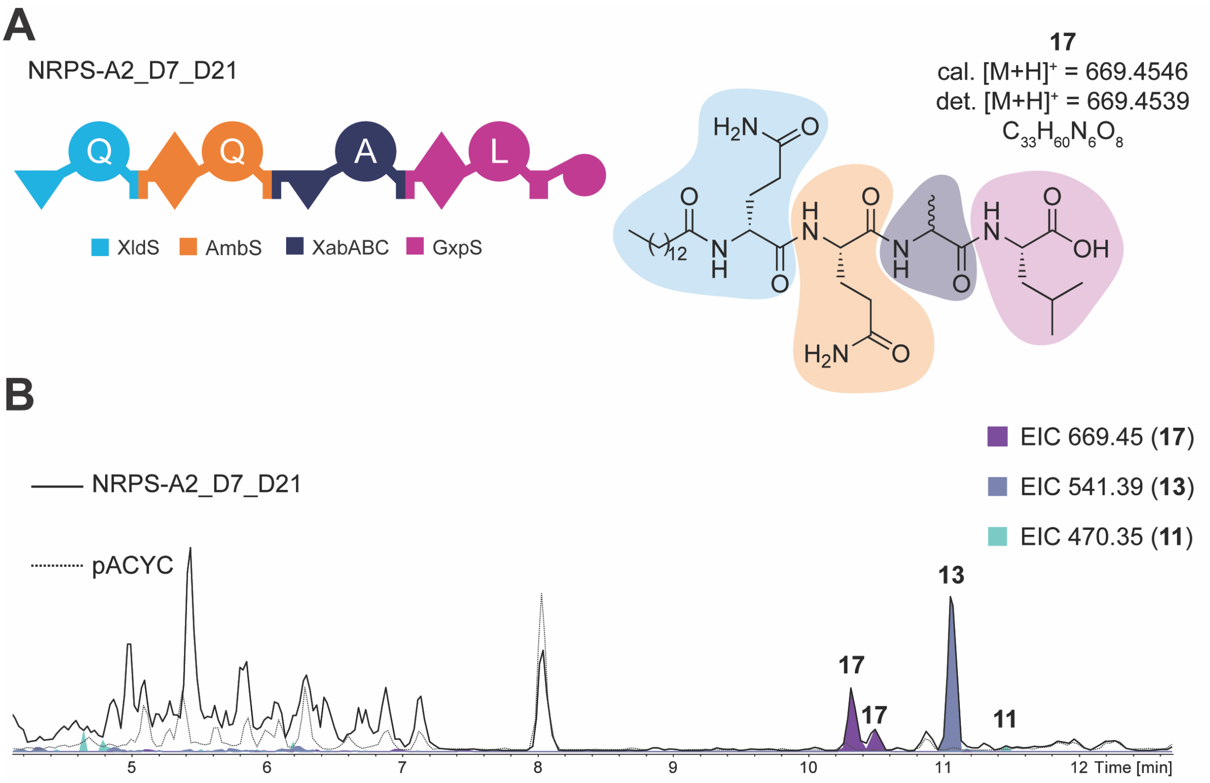

Figure S8. HPLC/MS analysis of the produced NRPS-A2_D7_D21 in *E. coli* DH10B*::mtaA*. A) Schematic representation of NRPS-A2_D7_D21 with the colour code of the original NRPS. The A domain specificities are indicated with Q = glutamine, A = alanine and L = leucine. Chemical structure of the peptide **17** with its molecular formula, the calculated mass and the measured high-resolution mass $[M+H]^+$. **B**) HPLC/MS data of compounds **17**, **13** (see Fig. S4) and **11** (see Fig. S5) produced in *E. coli* DH10B::*mtaA* expressing *NRPS-A2_D7_D21*. Base peak chromatogram (BPC, black line) and the extracted ion chromatograms (EIC, below with colours according to the depicted legend) of **17** (*m/z* $[M+H]^+$ = 669.45), **13** (*m/z* $[M+H]^+$ = 541.39) and **11** (*m/z* $[M+H]^+$ = 470.35). The pACYC expression was used as a negative control (dotted line).

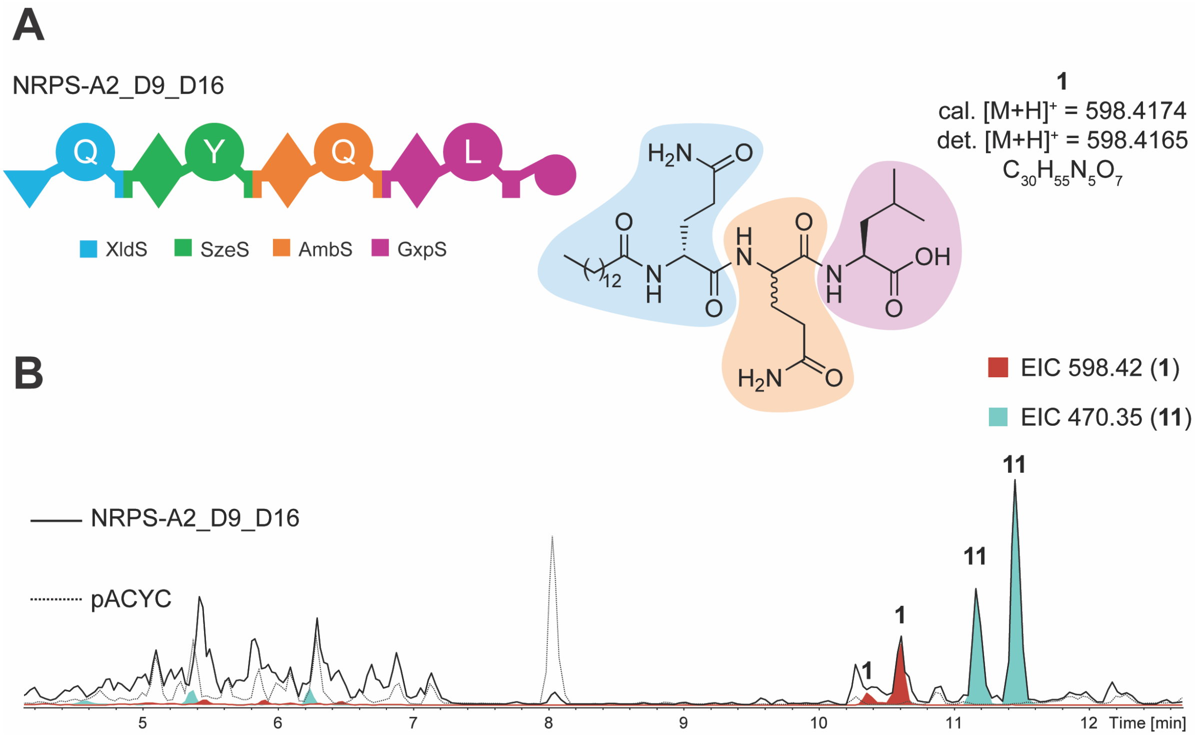

Figure S9. HPLC/MS analysis of the produced NRPS-A2_D9_D16 in *E. coli* DH10B*::mtaA*. A) Schematic representation of NRPS-A2_D9_D16 with the colour code of the original NRPS. The A domain specificities are indicated with Q = glutamine, A = alanine and L = leucine. Chemical structure of the peptide **1** with its molecular formula, the calculated mass and the measured high-resolution mass $[M+H]^+$. **B**) HPLC/MS data of compounds **1** and **11** (see Fig. S5) produced in *E. coli* DH10B::*mtaA* expressing *NRPS-A2_D7_D21*. Base peak chromatogram (BPC, black line) and the extracted ion chromatogram (EIC, below with colours according to the depicted legend) of **1** (*m/z* $[M+H]^+$ = 598.42) and **11** (*m/z* $[M+H]^+$ = 470.35). The pACYC expression was used as a negative control (dotted line).

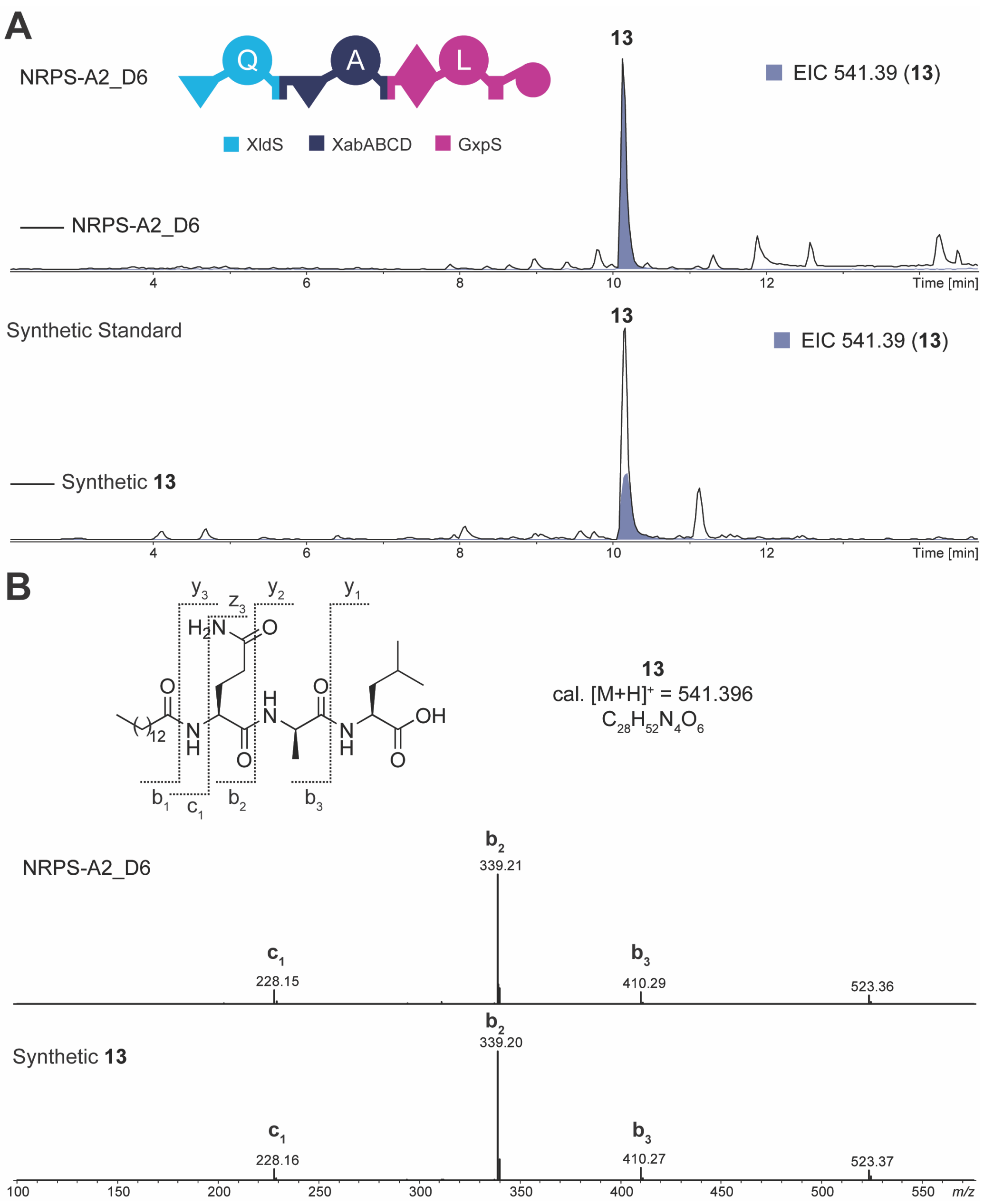

Figure S10. Comparison of the HPLC/MS analysis between compound 13 produced by NRPS-A2_D6 and the synthetic 13. A) Schematic representation of NRPS-A2_D6 with the colour code of the original NRPS. The A domain specificities are indicated with Q = glutamine, A = alanine and L = leucine. HPLC/MS data of compounds **13** and produced in *E. coli* DH10B::*mtaA* expressing *NRPS-A2_D6* and the synthetic standard **13**. Base peak chromatograms (BPC, black liness) and the extracted ion chromatogram (EIC, below with colours according to the depicted legend) of **13** (*m/z* $[M+H]^+$ = 541.39). **B**) Comparison of MS^2 spectra. The compound **13** fragmentation was compared between the NRPS-produced one and the synthetic standard. Fragments are indicated in the MS^2 spectra and the chemical structure.

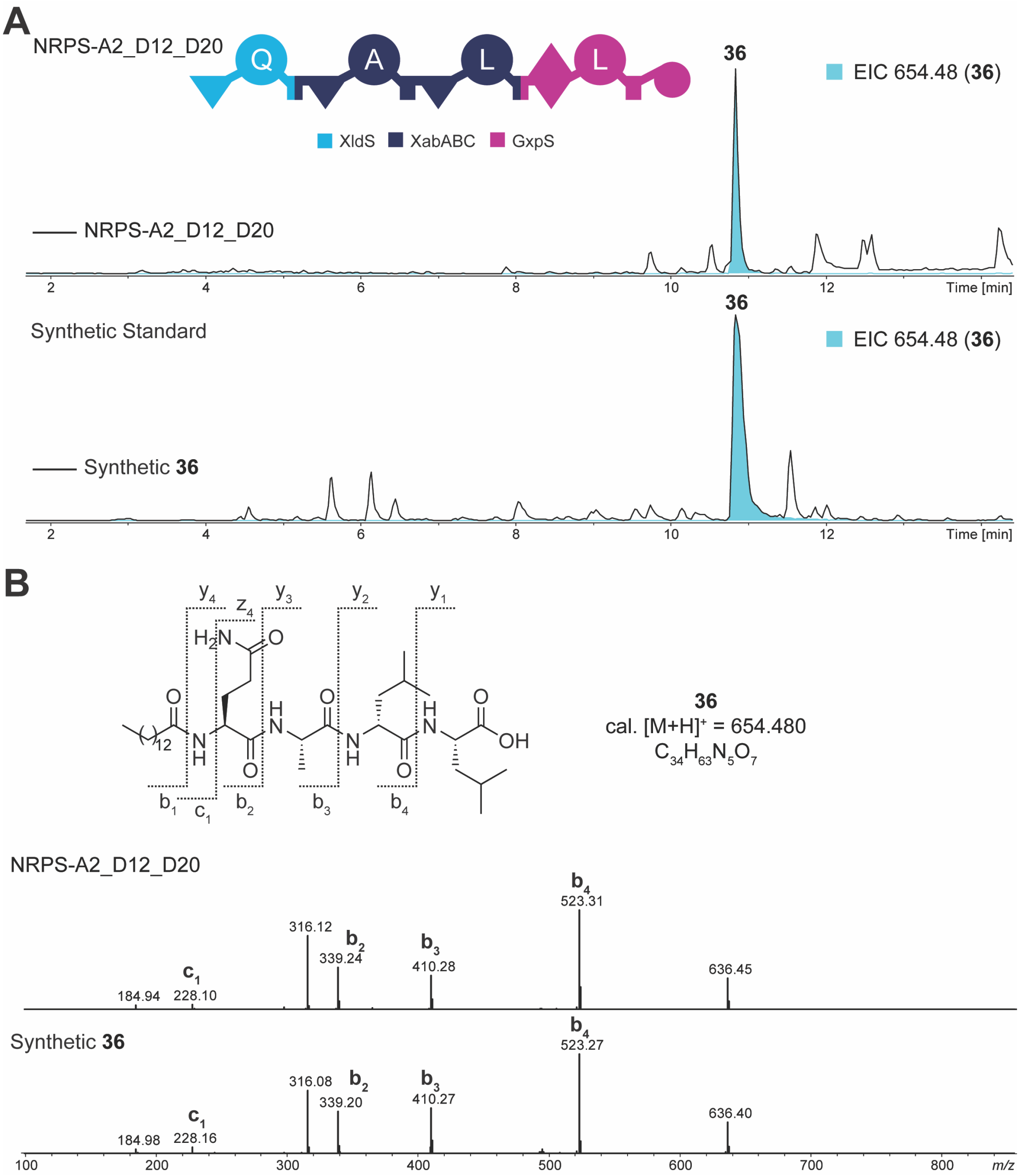

Figure S11. Comparison of the HPLC/MS analysis between compound 36 produced by NRPS-A2_D12_D20 and the synthetic 36. A) Schematic representation of NRPS-A2_D12_D20 with the colour code of the original NRPS. The A domain specificities are indicated with Q = glutamine, A = alanine and L = leucine. HPLC/MS data of compounds **36** and produced in *E. coli* DH10B::*mtaA* expressing *NRPS-A2_D12_D20* and the synthetic standard **36**. Base peak chromatogram (BPC, black lines) and the extracted ion chromatogram (EIC, below with colours according to the depicted legend) of **36** (*m/z* $[M+H]^+$ = 654.48). **B**) Comparison of MS^2 spectra. The compound **36** fragmentation was compared between the NRPS-produced one and the synthetic standard. Fragments are indicated in the MS^2 spectra and the chemical structure.

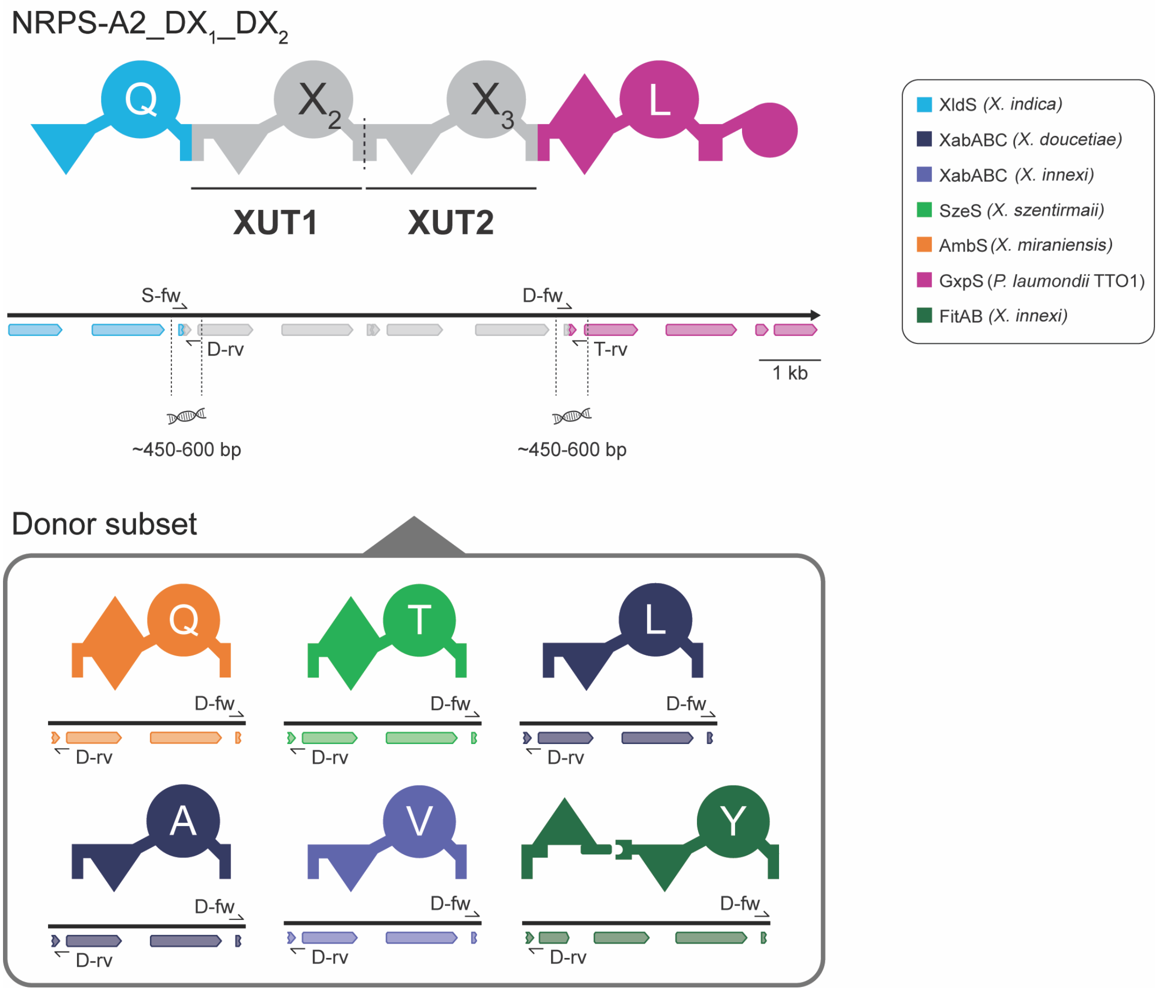

Figure S12. Schematic overview of the primer allocation of the qPCR analysis for NRPS plasmid validation. Domain annotations are included below the XUT. Acceptor plasmids contain a forward primer in the coding region of the starter module (S-fw) and a reverse primer in the coding region of the termination module (T-rv). Donors have both primers designed and allocated at the gene regions of the end of the A domain (D-fw) and within the T-C linker or the start of the C domain (D-rv). During qPCR, amplicons of ~450 – 600 bp are created.

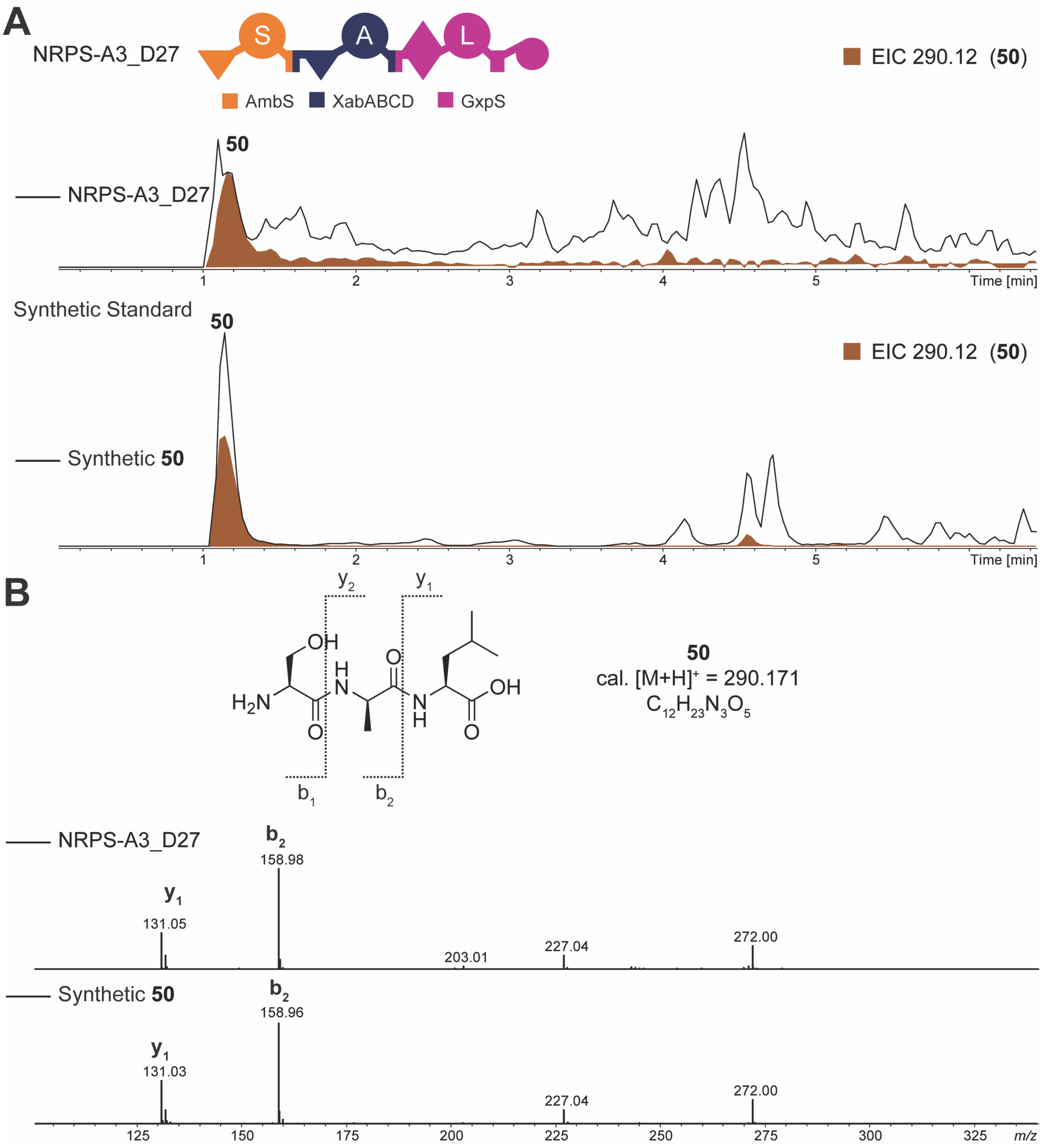

Figure S13. Comparison of the HPLC/MS analysis between compound 50 produced by NRPS-A3_D27 and the synthetic 50. A) Schematic representation of NRPS-A3_D27 with the colour code of the original NRPS. The A domain specificities are indicated with S = serine, A = alanine and L = leucine. HPLC/MS data of compounds **50** and produced in *E. coli* DH10B::*mtaA* expressing *NRPS-A3_D27* and the synthetic standard **50**. Base peak chromatograms (BPC, black lines) and the extracted ion chromatogram (EIC, below with colours according to the depicted legend) of **50** (*m/z* $[M+H]^+$ = 290.12). **B**) Comparison of MS^2 spectra. Compound **50** fragmentation was compared between the NRPS-produced one and the synthetic standard. Fragments are indicated in the MS^2 spectra and the chemical structure.

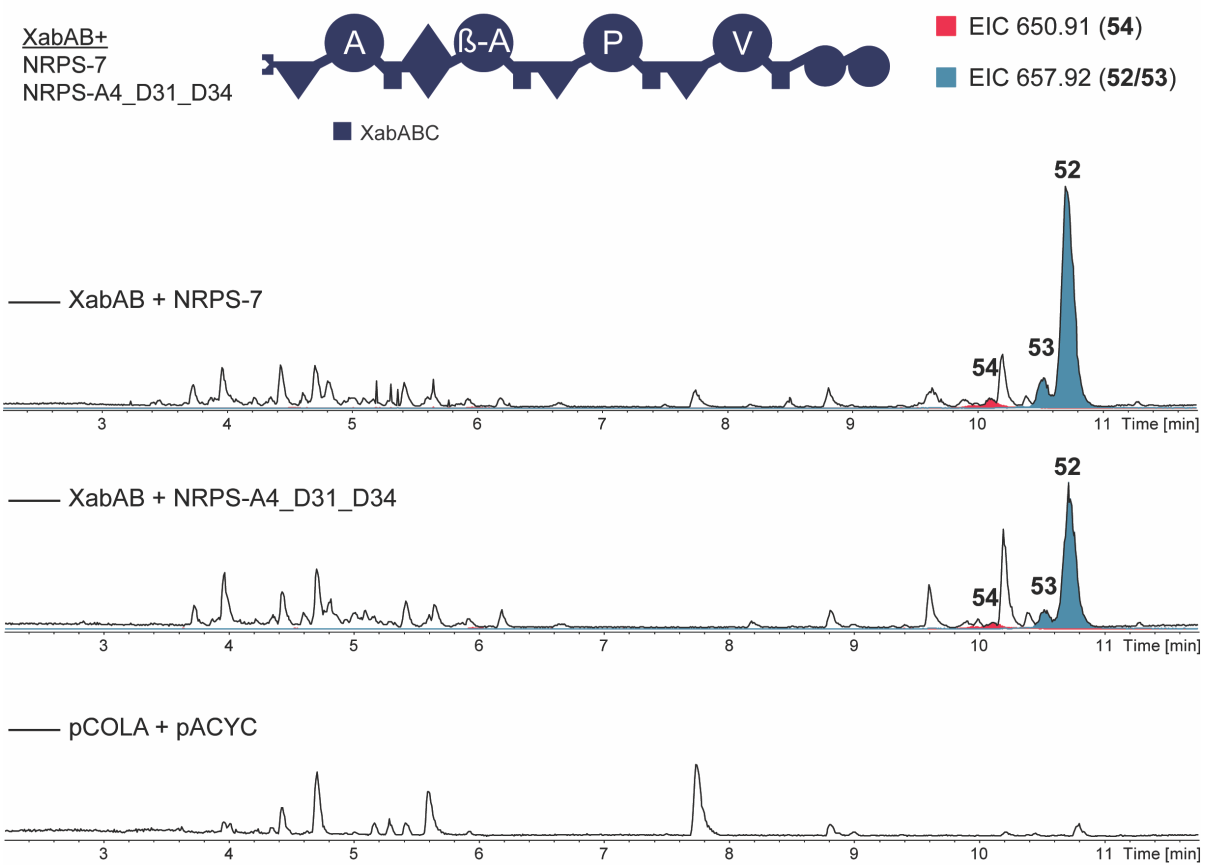

Figure S14. HPLC/MS analysis of the produced NRPS-7 and NRPS-A4_31_D34 in *E. coli* DH10B*::mtaA*. Schematic representation of NRPS-7 and NRPS A4_31_D34 with the colour code of the original NRPS. The A domain specificities are indicated with A = alanine, β-A = beta-alanine, P = proline, and V = valine. HPLC/MS data of compounds **52** – **54** (xenoamicin A, B and C) produced in *E. coli* DH10B::*mtaA* co-expressing *xabAB* and *NRPS-7* or *xabAB* and *NRPS-A4_31_D34*. Base peak chromatograms (BPC, black lines) and the extracted ion chromatograms (EIC, below with colours according to the depicted legend) of **52** and **53** (*m/z* $[M+2H]^{2+}$ = 657.92) and **54** (*m/z* $[M+2H]^{2+}$ = 650.91). The pCOLA + pACYC expression was used as a negative control.

A

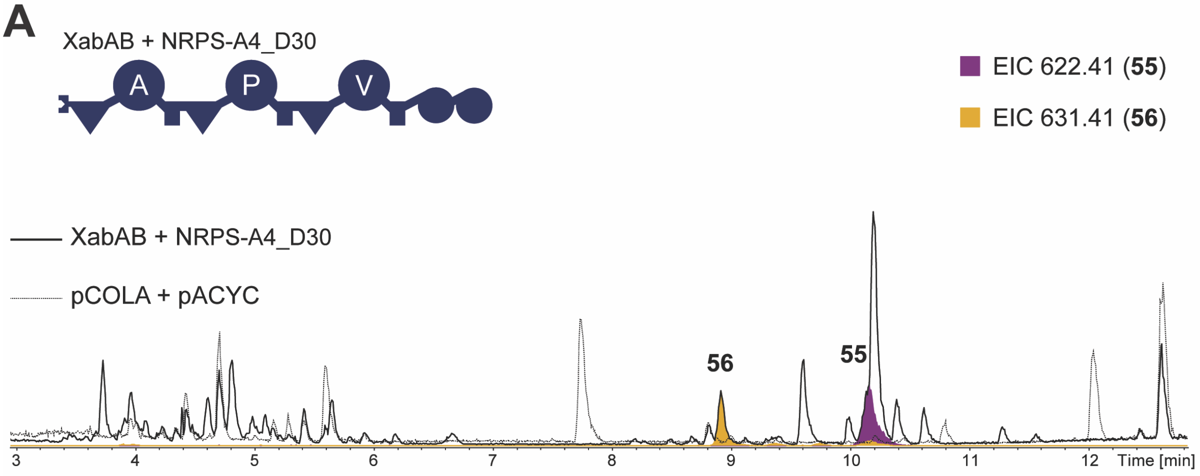

B

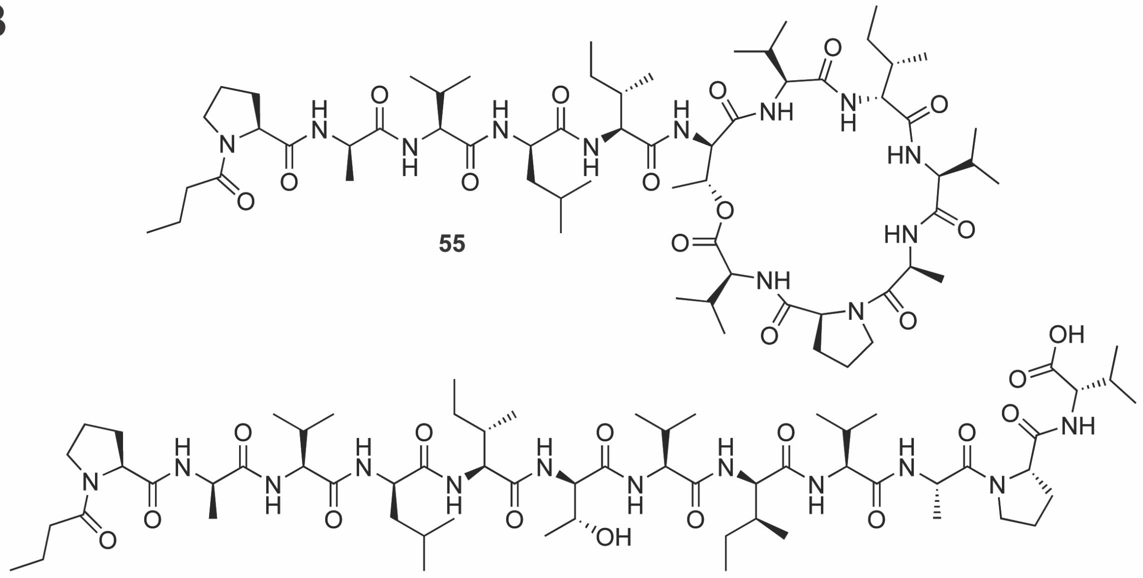

55

56

Figure S15. HPLC/MS analysis of the produced NRPS-A4_D30 in *E. coli* DH10B::*mtaA*. A) Schematic representation of NRPS-A4_D30 with the colour code of the original NRPS. The A domain specificities are indicated with A = alanine, P = proline, and V = valine. HPLC/MS data of compounds **55** (cyclic) and **56** (linear) produced in *E. coli* DH10B::*mtaA* expressing *xabAB* and *NRPS-A4_D30*. Base peak chromatogram (BPC, black line) and the extracted ion chromatograms (EIC, below with colours according to the depicted legend) of **55** (m/z $[M+2H]^{2+}$ = 622.41) and **56** (m/z $[M+2H]^{2+}$ = 631.41). The pCOLA + pACYC expression was used as a negative control (dotted line). **B**) Chemical structure of the peptides **55** and **56**.

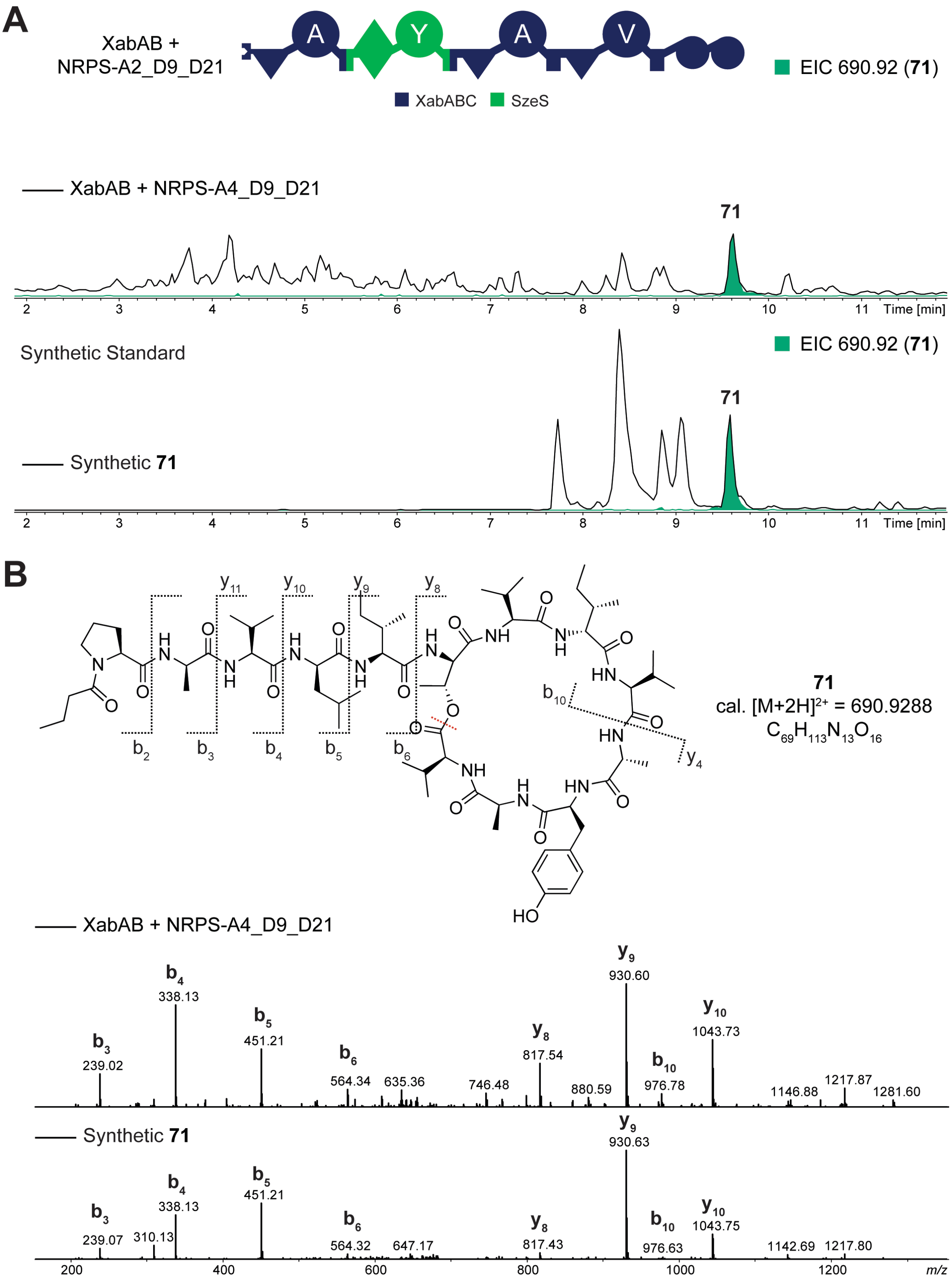

Figure S16. Comparison of the HPLC/MS analysis between compound 71 produced by NRPS-A4_D9_D21 and the synthetic 71. A) Schematic representation of NRPS-A4_D9_D21 with the colour code of the original NRPS. The A domain

specificities are indicated with A = alanine, Y = tyrosine, and V = valine. HPLC/MS data of compounds **71** and produced in *E. coli* DH10B::*mtaA* expressing *xabAB* and *NRPS-A4_D9_D21* and the synthetic standard of **71**. Base peak chromatograms (BPC, black lines) and the extracted ion chromatogram (EIC, below with colours according to the depicted legend) **71** (*m/z* $[M+H]^+$ = 690.92). **B**) Comparison of MS^2 spectra. The compound **71** fragmentation was compared between the NRPS-produced one and the synthetic standard. Fragments are indicated in the MS^2 spectra and the chemical structure. The red dotted line indicates linearisation for fragment b_{10}.

References

[1] E. Bode, A. K. Heinrich, M. Hirschmann, D. Abebew, Y. Shi, T. D. Vo, F. Wesche, Y. Shi, P. Grün, S. Simonyi, N. Keller, Y. Engel, S. Wenski, R. Bennet, S. Beyer, I. Bischoff, A. Buaya, S. Brandt, I. Cakmak, H. Çimen, S. Eckstein, D. Frank, R. Fürst, M. Gand, G. Geisslinger, S. Hazir, M. Henke, R. Heermann, V. Lecaudey, W. Schäfer, S. Schiffmann, A. Schüffler, R. Schwenk, M. Skaljac, E. Thines, M. Thines, T. Ulshöfer, A. Vilcinskas, T. A. Wichelhaus, H. B. Bode, *Angew. Chem. Int. Ed.* 2019, *58*, 18957–18963.

[2] S. Lear, S. L. Cobb, *J. Comput.-Aided Mol. Des.* 2016, *30*, 271–277.

[3] V. Potapov, J. L. Ong, B. W. Langhorst, K. Bilotti, D. Cahoon, B. Canton, T. F. Knight, T. C. Evans, G. J. S. Lohman, *Nucleic Acids Res.* 2018, *46*, e79–e79.

[4] J. M. Pryor, V. Potapov, R. B. Kucera, K. Bilotti, E. J. Cantor, G. J. S. Lohman, *PLoS ONE* 2020, *15*, e0238592.

[5] A. P. Sikkema, S. K. Tabatabaei, Y. Lee, S. Lund, G. J. S. Lohman, *Curr. Protoc.* 2023, *3*, e882.

[6] K. A. J. Bozhüyük, L. Präve, C. Kegler, L. Schenk, S. Kaiser, C. Schelhas, Y.-N. Shi, W. Kuttenlochner, M. Schreiber, J. Kandler, M. Alanjary, T. M. Mohiuddin, M. Groll, G. K. A. Hochberg, H. B. Bode, *Science* 2024, *383*, eadg4320.

[7] A. Untergasser, I. Cutcutache, T. Koressaar, J. Ye, B. C. Faircloth, M. Remm, S. G. Rozen, *Nucleic Acids Res.* 2012, *40*, e115–e115.

[8] T. A. Lindeboom, M. del C. S. Olmos, K. Schulz, C. K. Brinkmann, A. A. R. Rojas, L. Hochrein, D. Schindler, *ACS Synth. Biol.* 2024, *13*, 1116–1127.

[9] O. Schimming, F. Fleischhacker, F. I. Nollmann, H. B. Bode, *ChemBioChem* 2014, *15*, 1290–1294.

[10] F. I. Nollmann, C. Dauth, G. Mulley, C. Kegler, M. Kaiser, N. R. Waterfield, H. B. Bode, *ChemBioChem* 2015, *16*, 205–208.

[11] F. I. Nollmann, A. Dowling, M. Kaiser, K. Deckmann, S. Grösch, R. ffrench-Constant, H. B. Bode, *Beilstein J. Org. Chem.* 2012, *8*, 528–533.

[12] C. Kegler, F. I. Nollmann, T. Ahrendt, F. Fleischhacker, E. Bode, H. B. Bode, *ChemBioChem* 2014, *15*, 826–828.

[13] Q. Zhou, F. Grundmann, M. Kaiser, M. Schiell, S. Gaudriault, A. Batzer, M. Kurz, H. B. Bode, *Chem. A Eur. J.* 2013, *19*, 16772–16779.

[14] W. Lorenzen, T. Ahrendt, K. A. J. Bozhüyük, H. B. Bode, *Nat. Chem. Biol.* 2014, *10*, 425–427.

[15] K. A. J. Bozhueyuek, J. Watzel, N. Abbood, H. B. Bode, *Angew. Chem. Int. Ed.* 2021, *60*, 17531–17538.

[16] R. Silva-Rocha, E. Martínez-García, B. Calles, M. Chavarría, A. Arce-Rodríguez, A. de las Heras, A. D. Páez-Espino, G. Durante-Rodríguez, J. Kim, P. I. Nikel, R. Platero, V. de Lorenzo, *Nucleic Acids Res.* 2012, *41*, D666–D675.

[17] E. Martínez-García, T. Aparicio, A. Goñi-Moreno, S. Fraile, V. de Lorenzo, *Nucleic Acids Res.* 2015, *43*, D1183–D1189.
